# Effects of genetic ablation and pharmacological inhibition of HuR on gene expression, iron metabolism, and hormone levels

**DOI:** 10.1186/s12915-025-02131-z

**Published:** 2025-01-23

**Authors:** Nathalie Idlin, Sivakumar Krishnamoorthy, Magdalena Wolczyk, Mouad Fakhri, Michal Lechowski, Natalia Stec, Jacek Milek, Pratik Kumar Mandal, Jaroslaw Cendrowski, Christos Spanos, Magdalena Dziembowska, Katarzyna Mleczko-Sanecka, Juri Rappsilber, Gracjan Michlewski

**Affiliations:** 1 International Institute of Molecular and Cell Biology in Warsaw, Warsaw, Poland; 2 Department of Animal Physiology, Faculty of Biology, University of Warsaw, Warsaw, Poland; 3 Maria Sklodowska-Curie National Research Institute of Oncology, Warsaw, Poland; 4 The Wellcome Centre for Cell Biology, University of Edinburgh, Edinburgh, UK; 5 Department of Biotechnology, Technische Universität Berlin, Berlin, Germany

**Keywords:** HuR, ELAVL1, Eltrombopag, Iron metabolism

## Abstract

**Background:**

HuR/ELAV1, a ubiquitous RNA-binding protein, belongs to the RNA-binding protein family and is crucial for stabilizing and regulating the translation of various mRNA targets, influencing gene expression. Elevated HuR levels are associated with multiple disorders, including cancer and neurodegenerative diseases. Despite the identification of small molecule inhibitors targeting HuR, their detailed characterization remains limited. Recently, Eltrombopag, an FDA-approved drug for immune thrombocytopenic purpura and chemotherapy-induced thrombocytopenia, emerged as a potential HuR inhibitor. However, the specific molecular pathways influenced by both HuR and Eltrombopag are not fully understood.

**Results:**

Our study demonstrates that Eltrombopag operates via HuR inhibition, affecting gene expression regulation at the posttranscriptional level. We show that both HuR knockout and Eltrombopag treatment modulate iron metabolism by decreasing ferritin heavy chain (FTH1) and light chain (FTL) synthesis while increasing the expression of iron-regulatory protein 2 (IRP2), a key regulator of ferritin translation. Additionally, HuR inhibition reduces the levels of glycoprotein hormones, alpha polypeptide (CGA), a marker associated with hormone-induced tumors, suggesting a potential use of Eltrombopag in treatment of cancers overexpressing CGA. We observed that the main of control is manifested at the level of translation inhibition, with proteasome-mediated regulation also playing an important role.

**Conclusions:**

These findings uncover novel posttranscriptional mechanisms governed by HuR and its inhibitor, elucidating pathways relevant to HuR-mediated regulation and molecular therapies aimed at targeting this protein.

**Supplementary Information:**

The online version contains supplementary material available at 10.1186/s12915-025-02131-z.

## Background

RNA-binding proteins (RBPs) regulate gene expression through various mechanisms such as RNA splicing, transport, localization, translation, and turnover [1]. All these processes participate in physiology and their misregulation contributes to a plethora of human diseases. Understanding the molecular mechanisms of the RBPs associated with the etiology of these diseases will be crucial for designing novel and effective molecular therapies for heritable and nonheritable disorders [2, 3]. By targeting specific RBPs, it should be possible to correct altered gene expression networks and associated molecular processes, thus providing a more systemic approach to therapy.

Human antigen R (HuR/ELAVL1) is a ubiquitously expressed protein that belongs to the Hu family of RBPs [4, 5]. It plays a crucial role in posttranscriptional regulation by binding to target mRNAs, influencing their translation efficiency and stability [6]. HuR exerts its function by binding to AU-rich elements (AREs) [7, 8]. Recent research has shed light on the multifaceted functions of HuR in human diseases, including cancer [9], neurodegenerative disorders [10], inflammatory diseases [11], and viral infections [12]. Altered HuR function has been observed in various types of cancer, promoting cell survival, proliferation, angiogenesis, and metastasis [9, 13, 14]. Previous studies have demonstrated high levels of cytoplasmic HuR in oral, colorectal, gastric, lung, breast, ovarian, renal, skin carcinoma, and mesothelioma [14]. Thus, targeting HuR might have therapeutic potential to correct aberrant mRNA metabolism, thereby attenuating the underlying pathological processes.

Small molecules that inhibit HuR have been described but lack thorough characterization [9, 15–17]. These molecules target HuR in three different ways: by inhibiting its cytoplasmic translocation, by blocking its binding to target mRNAs, or by decreasing its expression [6]. Some of the identified inhibitors disrupt the HuR-RNA interaction, and their potency may be modulated by posttranslational HuR modifications [18]. The usage of these inhibitors could have unintended side effects because HuR is expressed ubiquitously, has many functions, and its complete depletion is lethal. Thus, a thorough evaluation of their modes of action is necessary for safe and effective molecular therapies.

**Fig. 1:**
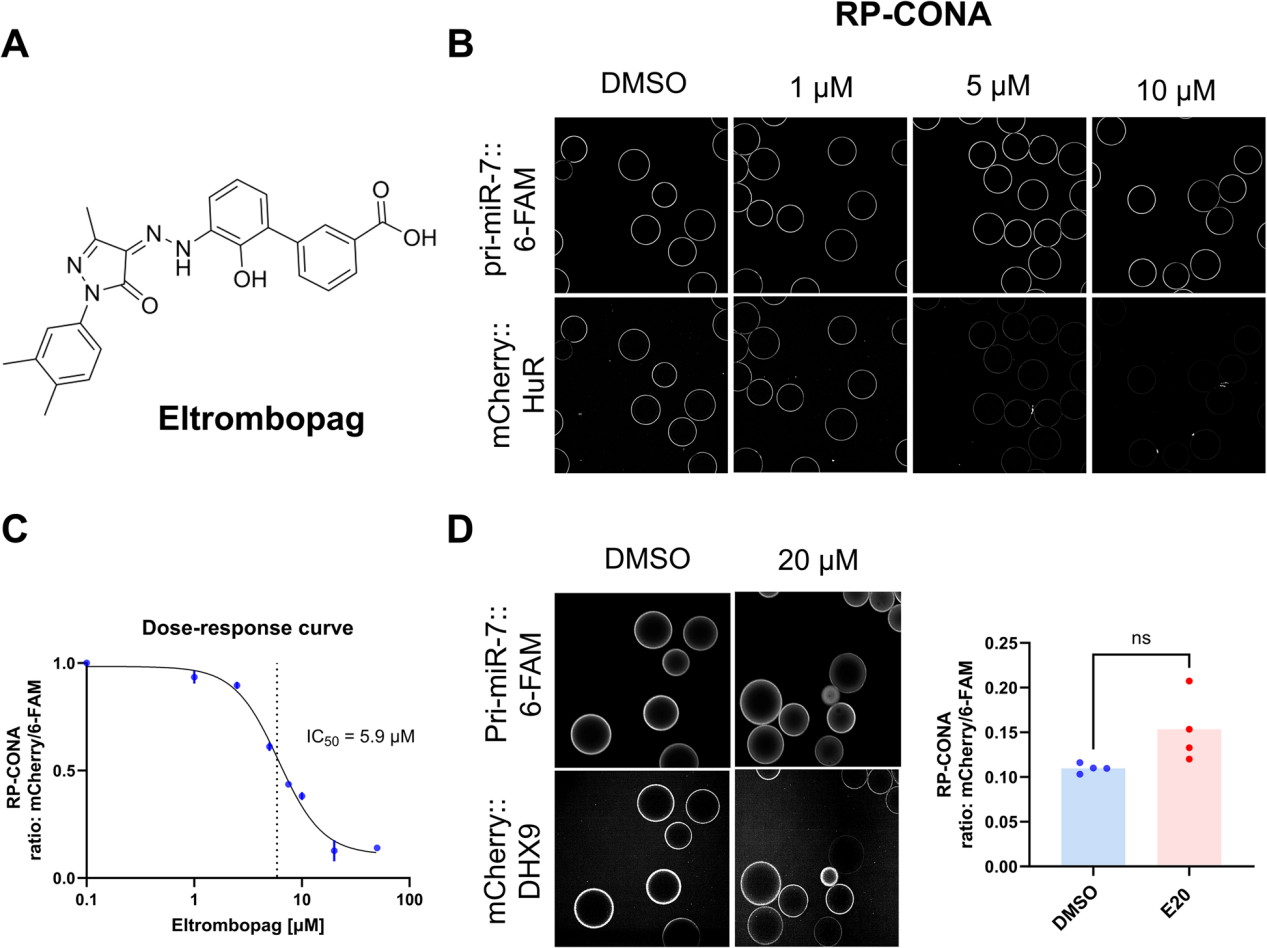
Eltrombopag disrupts HuR/pri-miR-7 interaction. **A** Representation of the chemical structure of Eltrombopag (E20). **B** Evaluation of the mCherry-HuR/pri-miR-7–6-FAM intensity ratio via RP-CONA at varying concentrations (1 µM, 5 µM, 10 µM) of Eltrombopag, in comparison to a mock (DMSO) control. Notably, the intensity ratio significantly decreased with 10 µM of Eltrombopag treatment. **C** Dose–response curve demonstrating the IC_50_ value of Eltrombopag, determined to be 5.9 µM. The IC_50_ was calculated using nonlinear regression from three replicas (4PL, GraphPad Prism Software 10.0.2, *R*_adj_.^2^ = 0.98). **D** Evaluation of RP-CONA with mCherry-DHX9/pri-miR-7–6-FAM treated with DMSO or 20 µM Eltrombopag. Representative image of RP-CONA results is shown in the left panel and quantification of fluorescence signal intensity ratio (mCherry-DHX9/pri-miR-7–6-FAM) from RP-CONA is denoted in the right panel. The data depicted is the mean with ± SEM of four replicates. Statistical significance was determined using a Student *t*-test, with “ns” indicating non-significant differences between treated and nontreated samples. Individual data values are presented in Additional file 2

Eltrombopag is a thrombopoietin receptor agonist (Fig. 1A), a safe and effective orally administered medication to treat chronic immune thrombocytopenic purpura (ITP) and chemotherapy-induced thrombocytopenia (CIP) [19]. Crucially, it has been shown to target HuR and elicit anti-angiogenic effects in breast cancer cells [20]. In a recent study, Eltrombopag has been shown to disrupt the complexes between HuR and the ARE in the 3′ untranslated region (3′UTR) of target mRNAs, such as Snail, Cox-2, and Vegf-c [21]. This resulted in the destabilization of these mRNAs and decreased expression, thereby inhibiting breast cancer metastasis. Together, these findings provide a potential path toward repurposing the clinical application of Eltrombopag.

The molecular effects of targeting HuR-RNA interactions on a transcriptome and proteome-wide scale have yet to be documented. Here, we conducted molecular analyses comparing mRNA and protein levels in wild type (WT) and CRISPR/Cas9-mediated HuR knock out (KO) HeLa cells, along with Eltrombopag treatment. Our study reveals that Eltrombopag predominantly influences gene expression at the level of translation and protein degradation. We identified proteins regulated by both HuR KO and pharmacological inhibition, encompassing ferritins and glycoprotein hormone. Additionally, our study demonstrates elevated labile iron and reactive oxygen species under these conditions. Our findings unveil novel posttranscriptional events governed by HuR and its FDA-approved inhibitor, offering valuable insights into Eltrombopag’s cellular impact and the potential targeting of HuR in molecular therapies.

## Results

### Eltrombopag inhibits HuR-RNA interaction

To explore Eltrombopag’s impact on HuR-RNA interactions, we utilized the RNA pull-down confocal nanoscanning (RP-CONA) method in HeLa cell extracts [22]. This technique involves a pull-down assay conducted in extracts from cultured cells, enabling the detection of modulators affecting RNA–protein complexes. Previously, we have demonstrated that HuR binds to a conserved terminal loop (CTL) of miR-7 primary transcript (pri-miR-7) [17, 22, 23]. By analyzing the fluorescence signal intensity ratio of mCherry-HuR/pri-miR-7–6-FAM during Eltrombopag dose-titration (0–50 μM), we observed a reduction in HuR-RNA binding with an IC50 value of 5.9 μM (Fig. 1B). In subsequent experiments, we selected a concentration of 20 μM for Eltrombopag as it displayed the highest inhibition (80%) of HuR-RNA interaction. Crucially, Eltrombopag did not quench the mCherry control protein signal at any tested concentration (Additional file 1: Fig. S1), nor did it interfere with the binding of the mCherry-DHX9 protein to pri-miR-7–6-FAM in RP-CONA assays (Fig. 1C, D). Likewise, an orthogonal fluorescence anisotropy method with recombinant, purified, label-free HuR showed an IC50 of 19 μM (Additional file 1: Fig. S2), which aligns with the low μM IC50 value from RP-CONA given the distinct experimental conditions. These results support Eltrombopag’s potential as a reliable HuR inhibitor.

### Eltrombopag effects on transcriptome are HuR independent

**Fig. 2:**
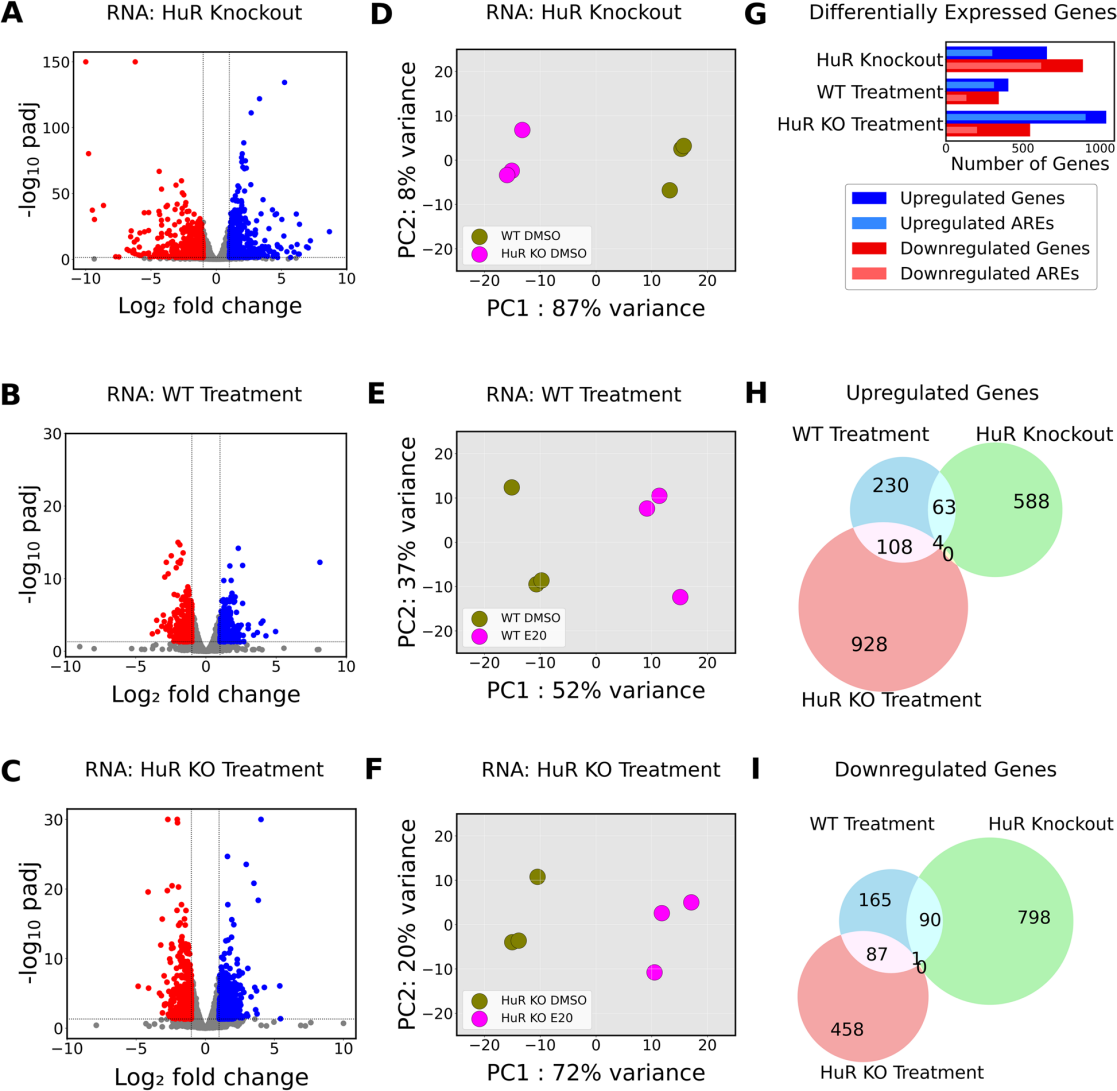
Eltrombopag’s impact on the transcriptome is independent of HuR. Volcano plots illustrating differentially expressed (DE) genes (padj < 0.05 and fold change ± 2) in **A** HuR KO, **B** WT treatment with Eltrombopag, and **C** HuR KO treatment with Eltrombopag, accompanied by corresponding principal components plots (**D**, **E**, **F**). Upregulated genes are denoted in blue, while downregulated genes are indicated in red. **G** Up and downregulated genes across the three comparisons using Euler diagrams along with respective AREs. Upregulated AREs are indicated in light blue and downregulated AREs are denoted in light red. The overlap between **H** upregulated mRNAs and **I** downregulated mRNAs among the three comparisons

Next, we assessed overall changes in mRNA levels in HeLa cells treated with Eltrombopag and subjected to HuR KO. While treatment up to 24 h did not affect cell viability, a statistically significant decrease was observed after 48 h in WT cells but not in KO cells (Additional file 1: Fig. S3). Therefore, we selected the 48-h time point for our analyses. Using RNAseq on samples obtained 48 h post treatment, a total of 16,387 mRNAs were detected in the control and experimental groups. We have initially performed two biological replicates and then added one more to have more robust results of the RNAseq. We compared the influence of Eltrombopag treatment on both WT and HuR KO cells, as well as the effects of HuR KO alone (Fig. 2A–F). The mRNA expression patterns in the Eltrombopag treatment groups (WT and HuR KO) showed small but similar levels of dysregulation (Fig. 2A–C), whereas dysregulation in HuR KO cells compared to WT cells was more substantial (Fig. 2A–C). Evaluation of the RNA-seq results through principal component analysis (PC1) showed that more than 52% variance is observed in all three comparisons (Fig. 2D–F). Combined PC analysis revealed distinct separation of WT and HuR KO samples along PC1 (61% variance), indicating batch specific transcriptional variation (Additional file 1: Fig. 4). The mRNA expression profiles clearly separated the sample groups, highlighting the effects mediated by both HuR KO and Eltrombopag treatment. Among the three comparisons, the number of differentially expressed (DE) genes was the most pronounced due to the treatment with Eltrombopag in HuR KO cells (1586) and in HuR KO cell (1544) surpassing the response to Eltrombopag in WT cells (748) (Fig. 2G).

Comparison of DE genes across the three experimental conditions showed a partial overlap in both upregulated and downregulated mRNAs (Fig. 2H, I). Eltrombopag treatment had similar effects whether HuR was present or absent (Fig. 2B, C), indicating that Eltrombopag drives transcriptional changes independently of HuR. Specifically, only 67 upregulated and 91 downregulated genes overlapped between WT Eltrombopag treatment and HuR KO. Such small overlap could be the result of HuR KO cells adaptation or Eltrombopag regulation of other pathways. One hundred twelve upregulated and 88 downregulated genes were shared between WT and HuR KO under Eltrombopag treatment, respectively (Fig. 2H, I). Notably, in HuR KO cells, 46% of upregulated and 70% of downregulated mRNAs contained ARE motifs (Fig. 2G), consistent with HuR’s known role in stabilizing ARE-containing mRNAs. In Eltrombopag-treated cells, ARE motifs accounted for 77% of upregulated and 38% of downregulated mRNAs in WT cells, and 87% of upregulated and 37% of downregulated mRNAs in HuR KO cells. Functional enrichment analysis of the DE genes due to Eltrombopag treatment showed enrichment for GO terms related to “steroid biosynthesis,” “metabolic pathways,” and “pathways in cancer” (Additional file 1: Fig. S5). In summary, RNAseq analyses identified alterations in gene expression in response to Eltrombopag treatment, with enrichment of metabolic pathways. Comparisons between WT and HuR KO cells indicated that the transcriptional effects of Eltrombopag are largely HuR-independent.

### Eltrombopag effects on proteome are HuR dependent

**Fig. 3:**
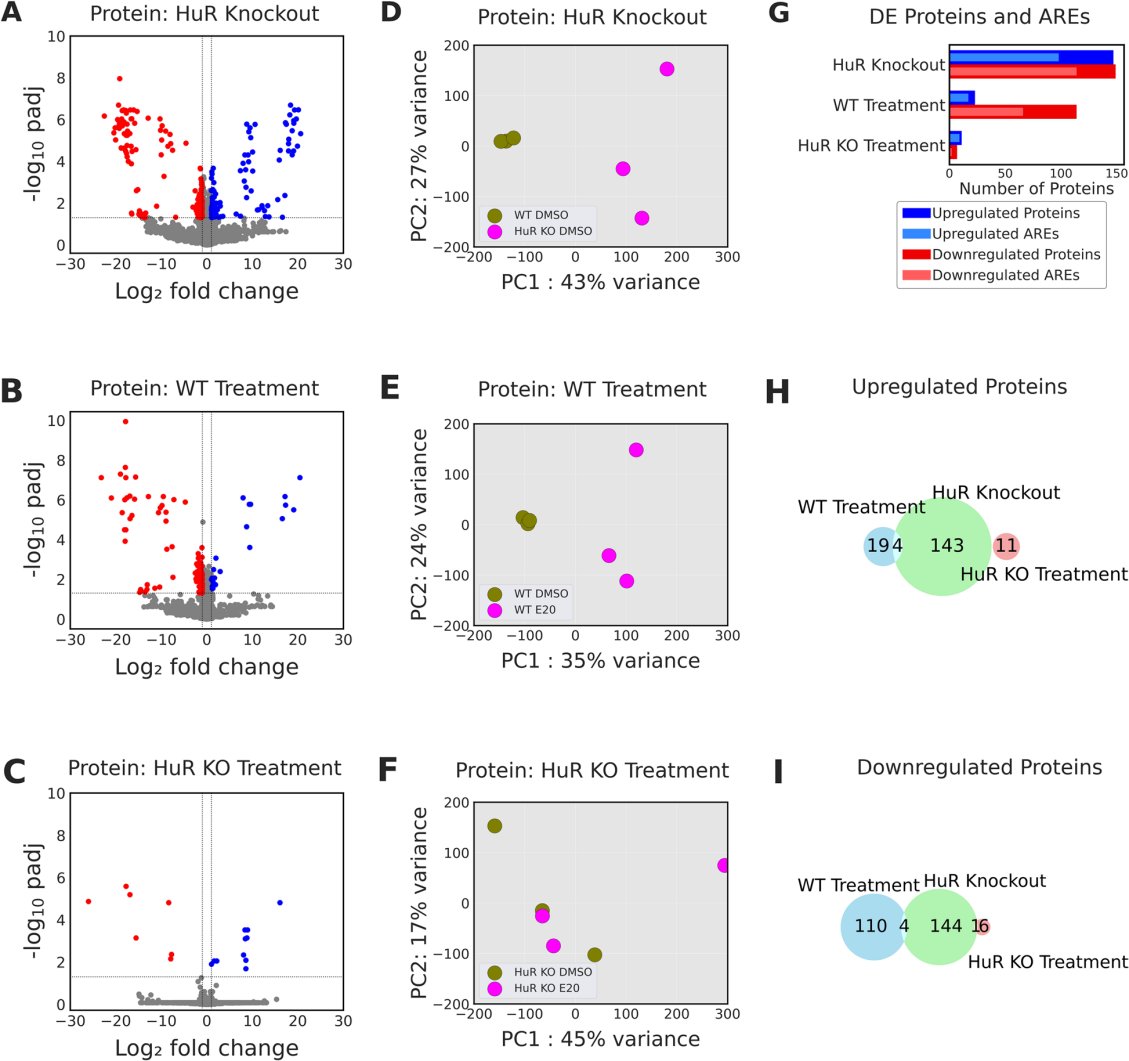
Eltrombopag’s impact on the proteome is HuR-dependent. Volcano plots illustrating differential protein levels (DL) (padj < 0.05 and fold change ± 2) for **A** HuR KO, **B** WT treatment with Eltrombopag, and **C** HuR KO treatment with Eltrombopag, along with corresponding principal components plots (**D**, **E**, **F**). Upregulated proteins are denoted in blue, while downregulated proteins are indicated in red. **G** Number of up and downregulated DL of proteins across the three comparisons and its respective mRNAs containing AREs. Euler diagrams depict the overlap between **H** upregulated proteins and **I** downregulated proteins across the three comparisons

To investigate Eltrombopag’s regulatory role at the protein level, we conducted analyses of differential levels (DL) of proteins using a label-free quantitative mass spectrometry DEqMS approach. The experimental parameters mirrored those employed in our RNASeq data analysis. A total of 8913 proteins were detected across the sample groups. The patterns of protein levels differed between the three studied comparisons (Fig. 3A, B, C). Notably, principal component 1 (PC1) illustrated that the variance accounted for more than 35% in all three comparisons (Fig. 3D, E, F). Combined PCA reveals that clear separation of WT and HuR KO groups along PC1, while PC2 distinguished treatment effect in WT, highlighting distinct proteomic changes driven by genotype and Eltrombopag’s treatment (Additional file 1: Fig. S6).

We noticed more DL of proteins in HuR KO (296) than in Eltrombopag treatment of either WT (137) or HuR KO cells (18) (Fig. 3A–G). Eltrombopag-treated and untreated cells revealed 23 upregulated and 114 downregulated proteins in WT cells. In contrast, only 11 proteins were upregulated and 7 were downregulated in response to Eltrombopag in HuR KO cells (Fig. 3H, I). Furthermore, in HuR KO cells, 66% of upregulated and 76% of downregulated proteins were encoded by mRNAs containing ARE motifs (Fig. 3G). In Eltrombopag-treated WT cells, ARE motifs were present in mRNAs encoding 74% of upregulated proteins and 58% of downregulated proteins. By contrast, in Eltrombopag-treated HuR KO cells, ARE motifs were found in mRNAs encoding 81% of upregulated and 28% of downregulated proteins. These results indicate that Eltrombopag regulates a larger proportion of downregulated proteins in WT cells compared to HuR KO cells, suggesting that the impact of Eltrombopag on the proteome relies at least partially on HuR presence.

Interestingly, only 4 proteins were downregulated (actin alpha 2 (ACTN2), basic leucine zipper nuclear factor 1 (BLZF1), glycoprotein hormones, alpha polypeptide (CGA), and dihydropyrimidine dehydrogenase (DYPD)), while 4 were upregulated (acyl-CoA synthetase short chain family member 1 (ACSS1), enhancer of zeste 1 polycomb repressive complex 2 subunit (EZH1), iron responsive element binding protein 2 (IREB2), and tumor protein P53 inducible nuclear protein 1 (TP53INP2)) in both HuR KO and WT treated cells (Fig. 3H, I). This limited overlap suggests potential compensatory mechanisms within HuR KO cells that shape the proteome. Alternatively, the action of Eltrombopag may induce further remodeling of gene expression, which is not reliant on HuR. Functional enrichment analysis reveals that GO terms related to “biosynthetic process” and “metabolic process” were enriched in HuR KO vs WT (Additional file 1: Fig. S7). In WT cells exposed to Eltrombopag, apart from “biosynthetic process,” KEGG pathways such as “non-fatty liver disease,” “Huntington disease,” and “carcinogenesis” were enriched. This indicates general disease-associated changes. In summary, quantitative proteomics analysis revealed that Eltrombopag shows HuR-dependent alteration of the proteome.

### Transcriptome-proteome comparison of HuR regulation

**Fig. 4:**
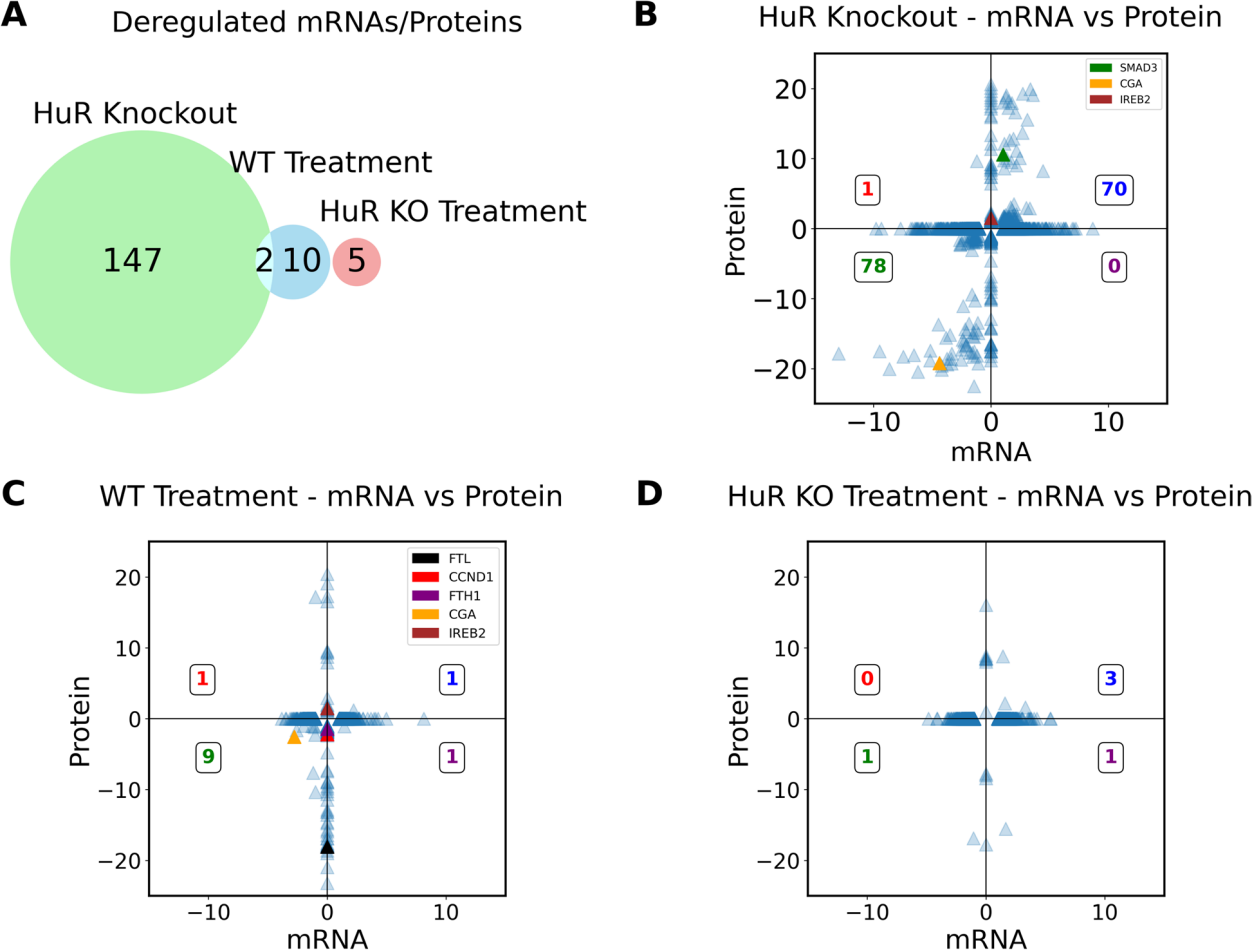
DE mRNAs and DL of proteins upon Eltrombopag treatment do not correlate. **A** Euler diagram illustrating the intersection of dysregulated mRNAs and proteins. Significant fold change values (padj < 0.05 and fold change ± 2) derived from both MS and RNAseq. The effects of **B** HuR Knockout, **C** WT Eltrombopag treatment, and **D** HuR KO Eltrombopag treatment. Six targets (SMAD3, FTL, IREB2, CGA, FTH1, and CCND1) highlighted in **B** and **C** become insignificant in **D**

Cross-comparison analysis performed between mRNA and protein levels revealed that few genes are dysregulated at both mRNA and protein levels due to the HuR KO (149) (Fig. 4A). Among them, 70 genes display enhanced mRNA and protein levels, while 78 genes show the opposite trend (Fig. 4B). On the contrary, the abundance of the majority of mRNA and coded protein pairs was not correlated in WT of HuR KO Eltrombopag treatment (Fig. 4C, D). This strongly suggests that HuR-mediated regulation by Eltrombopag predominantly regulates targets at the posttranscriptional level.

Subsequently, we analyzed individual mRNA-protein pairs. The expression of CGA exhibited downregulation at both protein and mRNA levels in both HuR KO and WT treatments, with no change observed in the HuR KO treatment alone (Fig. 4B–D and Additional file 1: Table S1). This underscores the HuR-dependent regulation of CGA by Eltrombopag. Importantly, ferritin light chain (FTL), ferritin heavy chain (FTH1), and cyclin D1 (CCND1) displayed significant downregulation solely at the protein level upon treatment of WT cells, but not in HuR KO cells (Fig. 4C and Additional file 1: Table S1). Additionally, IREB2, which codes for IRP2, exhibited upregulation both in HuR KO (Fig. 4B) and WT Eltrombopag treatment (Fig. 4C). This highlights the HuR-dependent alteration in expression of genes involved in iron metabolism induced by Eltrombopag. Of note, Smad family member 3 (SMAD3), which is also involved in iron homeostasis [24, 25], was significantly increased in HuR KO cells but remained unaltered in cells exposed to Eltrombopag (Fig. 4B and Additional file 1: Table S1). Together, these results indicate that Eltrombopag alters the expression of critical iron regulatory proteins and hormones in an HuR-dependent manner at the posttranscriptional level.

### Mechanisms of Eltrombopag’s activity

**Fig. 5:**
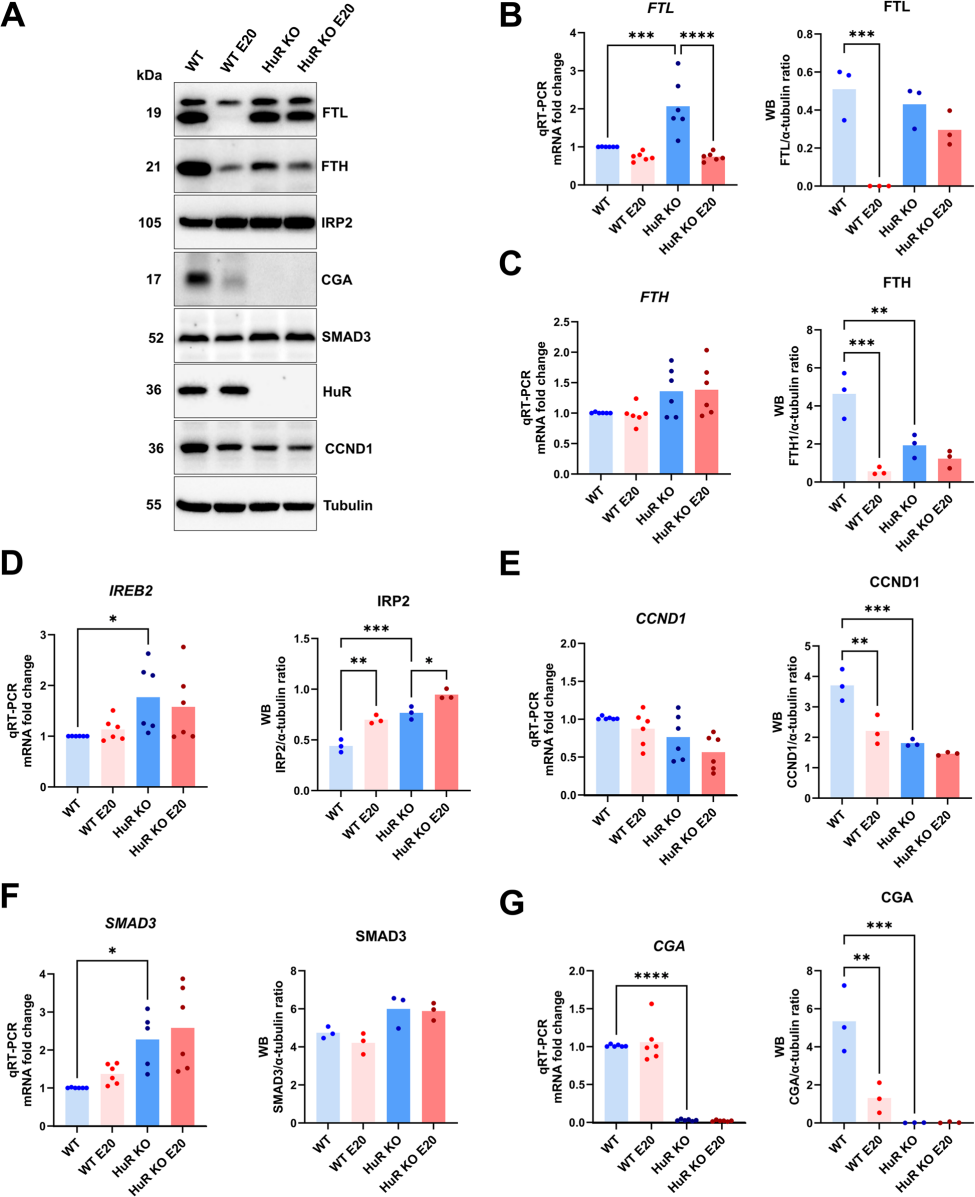
Selected targets are validated by qRT-PCR and western blot analyses. **A** Western blot of DL protein in WT, WT Eltrombopag (E20) treated, HuR KO, and HuR KO Eltrombopag treated. **B**–**G** Quantification of the results from western blot and qRT-PCR. **B** FTL, **C** FTH1, **D** IREB2 mRNA/IRP2 protein, **E** CCND1, **F** SMAD3, and **G** CGA. The data are represented as mean ± S.D., with *n* ≥ 3 per group per treatment for all studies. α-Tubulin served as an internal control for western blot method. Geometric means of GAPDH and ACTNB were utilized as normalized controls for qRT-PCR, with the results from WT cell set to 1. Statistical significance was determined using one-way ANOVA with Šidak’s multiple comparison adjustment with * denoting *p* < 0.05, ** denoting *p* < 0.01, *** denoting *p* < 0.001, and **** denoting *p* < 0.0001, indicating significant differences between conditions. Individual data values are presented in Additional file 2

**Fig. 6:**
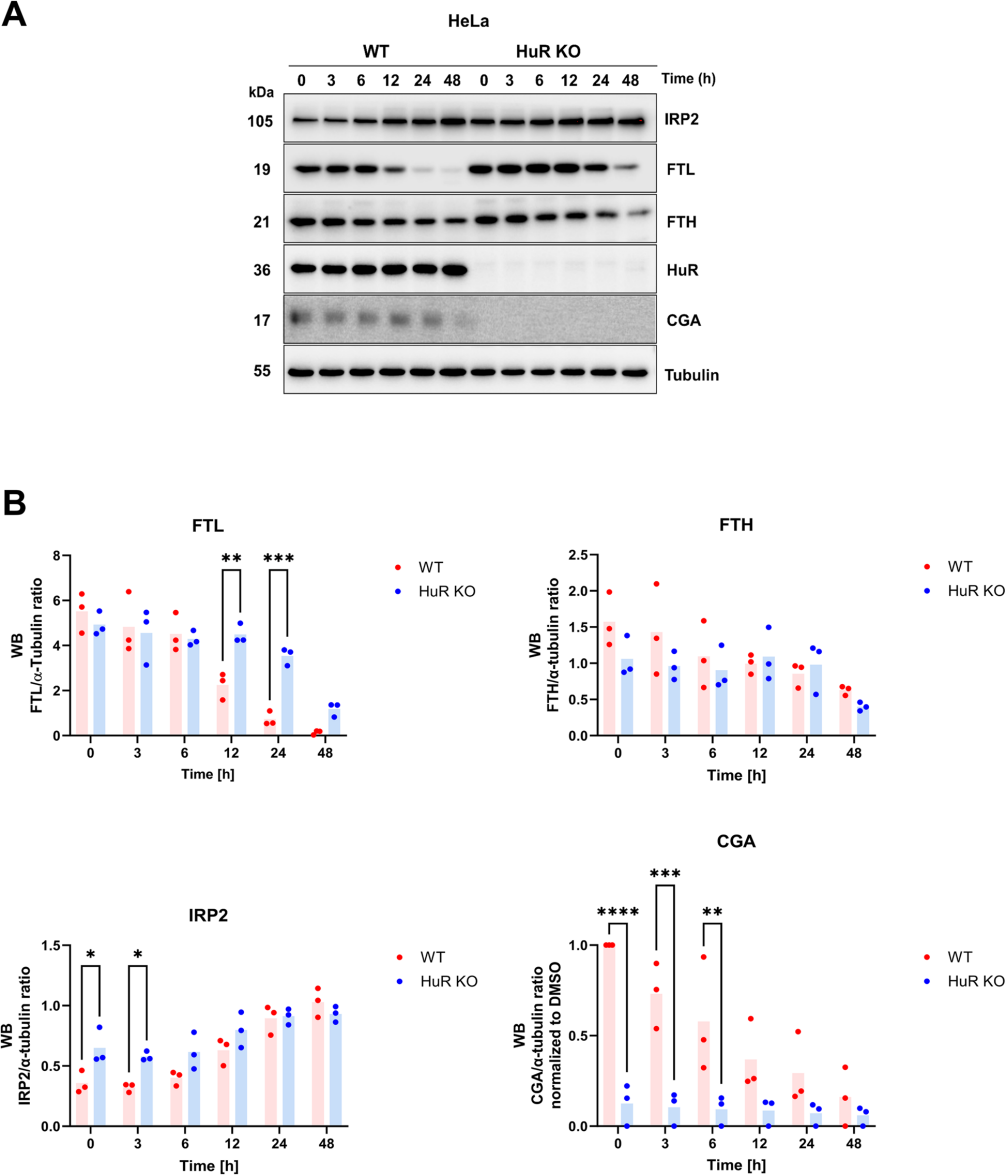
Time course of Eltrombopag treatment in WT and HuR KO cells reveals early changes in IRP2 levels. **A** Western blot depicting the time-dependent (0, 3, 6, 12, 24, and 48 h) expression of selected targets (IRP2, FTL, FTH1, CGA) following Eltrombopag treatment in WT and HuR KO cells. **B** Quantification of western blot results. The data are presented as mean ± S.D., with *n* = 3 per group per treatment for all studies. Statistical significance was determined using one-way ANOVA with Šidak’s multiple comparison adjustment, with * denoting *p* < 0.05, ** denoting *p* < 0.01, *** denoting *p* < 0.001, and **** denoting *p* < 0.0001, indicating significant differences between WT Eltrombopag treated and HuR KO Eltrombopag treated cells. Bar chart was indicated as mean ± SEM. α-Tubulin served as an internal control for western blotting. For CGA, the values were normalized to 1 based on the WT control, given the significant difference observed between WT and KO cells. Individual data values are presented in Additional file 2

Next, we validated the results by western blot analysis (Fig. 5A). The protein abundance of FTL, FTH1, CGA, and CCND1 was significantly decreased due to Eltrombopag treatment in WT cells (Fig. 5A–G). This effect was enhanced for CGA and CCND1, slightly mitigated for FTH1, and completely reversed for FTL in HuR KO untreated and Eltrombopag-treated cells (Fig. 5A–G). This suggests that FTL response to Eltrombopag is largely dependent on HuR. Additionally, IRP2 was significantly increased in HuR KO compared to WT cells (Fig. 5A, D). Time course treatment experiments revealed a reverse correlation between IRP2 and FTL levels in WT cells (Fig. 6A, B). This is consistent with the well-established negative regulation of FTL translation by IRP2 [26] and implies that Eltrombopag may indirectly regulate FTL via HuR-dependent effects on IRP2. To check for any effects at the RNA levels, the mRNA expression of selected transcripts was also validated by qRT-PCR. The results show an increase in SMAD3, FTL, and IREB2 and a decrease in CGA mRNAs in HuR KO cells (Fig. 5B–G). In summary, we reveal that Eltrombopag modulates the levels of iron regulatory proteins (FTL, FTH1, and IRP2), along with the CGA, in a HuR-dependent manner at the posttranscriptional level. The comparative analysis of mRNA and protein levels further supports the notion that HuR and Eltrombopag jointly regulate specific gene expression processes.

We next examined the effects of Eltrombopag in HuR knockdown (KD) cells, achieved through transient silencing of HuR using RNAi (Additional file 1: Fig. S8A, E). RNAi reduced HuR levels to approximately 25% of those observed in the DMSO-treated and scramble siRNA-treated controls. Notably, the steady-state levels of FTL and FTH1 proteins were significantly decreased in HuR KD cells without changes to their mRNA levels (Additional file 1: Fig. S8A, B, C). However, unlike in HuR KO cells treated with Eltrombopag (Fig. 5), we observed continued effective downregulation of FTL and FTH1 proteins upon treatment. Conversely, Eltrombopag treatment led to a significant upregulation of IRP2 protein levels only in DMSO-treated and scramble siRNA-treated cells, not in HuR KD cells, which exhibited higher basal levels of IRP2 (Additional file 1: Fig. S8A, D). Additionally, the downregulation of CCND1 protein upon Eltrombopag treatment was less pronounced in HuR KD cells compared to DMSO-treated or scramble siRNA-treated control cells (Additional file 1: Fig. S8A, F). However, no differences were in SMAD3 and CGA protein levels following Eltrombopag treatment between DMSO-treated, scramble siRNA-treated, or HuR KD cells. The partial overlap between KD and KO results may be attributed to incomplete depletion of HuR by siRNAs and potential adaptive mechanisms arising during the generation of HuR KO cells. Nevertheless, these findings reinforce the role of HuR in regulating selected proteins and underscore its interaction with Eltrombopag in these processes.

To further assess whether HuR is directly involved in the posttranscriptional regulation of the studied mRNAs, we performed HuR ribonucleoprotein immunoprecipitation (RIP) analysis. Endogenous HuR-RNA complexes were immunoprecipitated from HeLa WT and Eltrombopag-treated cells using an anti-HuR antibody. As a control for non-specific mRNA binding, a mouse normal IgG1 isotype antibody was employed. HuR was precipitated with comparable efficiency from both WT and Eltrombopag-treated cells (Additional file 1: Fig. S9A). In the HuR RIP assay, a notable enrichment of mRNAs immunoprecipitated by the HuR antibody, relative to the isotype IgG1 control, validated HuR’s binding to all tested mRNAs, including ACTB mRNA, a known HuR target [27]. Furthermore, Eltrombopag treatment significantly abolished HuR binding to CGA and IRP2, affected CCND1 binding without reaching statistical significance, and had no visible effect on HuR binding to FTH1, FTL, or mRNAs (Additional file 1: Fig. S9B). These findings suggest that not all HuR-mRNA interactions are equally influenced by Eltrombopag. This variability may result from differences in secondary and tertiary mRNA structures or the presence of other RNA-binding proteins that form specific interaction networks. Finally, our results indicate that some observed changes in protein levels, such as those for FTL and FTH1, may arise from secondary regulation by upstream effectors like IRP2.

**Fig. 7:**
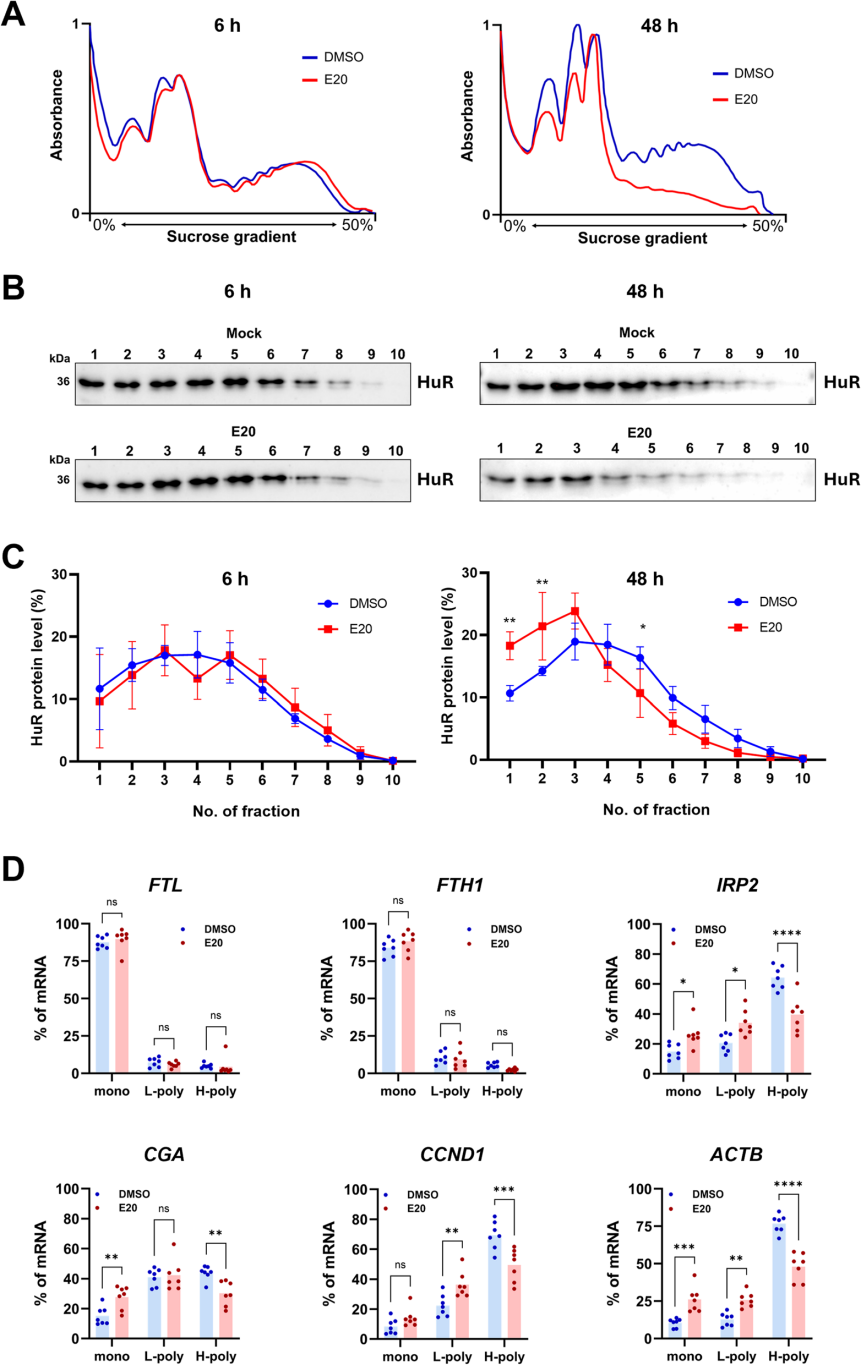
Polysome profiling reveals Eltrombopag-induced inhibition of global translation, reduced HuR protein accumulation in the polysomes. HeLa WT cells were subjected to DMSO or Eltrombopag (E20) treatment for 6 or 48 h, then sucrose gradient polysome fractionation was performed. **A** Polysome profile of HeLa WT cells treated either with DMSO or Eltrombopag for 6 h (left panel) or 48 h (right panel). HuR protein distribution in polysome fractions obtained from HeLa WT cells treated either with DMSO or Eltrombopag for 6 h (left panel) or 48 h (right panel) assessed by **B** western blot analysis and **C** densitometry quantification. **D** Percentage distributions of FTL, FTH1, IREB2, CGA, and CCND1 mRNAs in polysome fractions. Fractions were separated into three categories based on absorbance profile: monosome (mono), light polysome (L-poly), heavy polysome (H-poly). Data are presented as mean values with SEM with *n* = 7 per group per treatment for all samples for 48 h. Statistical significance was determined using two-way ANOVA with Šídák’s multiple comparisons test to find the treatment effect, with * denoting *p* < 0.05, ** denoting *p* < 0.01, *** denoting *p* < 0.001, **** for *p* < 0.0001, and ns denoting non-significant. TATAA Universal RNA Spike was utilized as normalization control for qRT-PCR. Individual data values are presented in Additional file 2

To expand on the previous findings and assess the impact of Eltrombopag treatment on the translation efficiency of all tested mRNAs, we performed polysome profiling analysis. HeLa cells were treated with either DMSO or Eltrombopag for 6 or 48 h. While Eltrombopag treatment did not induce any noticeable changes in the polysome profile at 6 h, a significant reduction in heavy polysome levels was observed after 48 h, indicating a direct inhibitory effect of Eltrombopag on global translation in HeLa cells (Fig. 7A). Western blot analysis of polysome fractions after 48 h of Eltrombopag treatment using an anti-HuR antibody, coupled with densitometric analysis of HuR distribution across the fractions, demonstrated that Eltrombopag treatment results in a significant accumulation of HuR in the monosome fractions (Fig. 7B, C). qRT-PCR analysis of mRNA levels in various polysome fractions after 48 h of Eltrombopag treatment revealed a decrease in translation efficiency for CCND1, CGA, and ACTB consistent with the observed reduction in protein levels (Fig. 5A, E). Interestingly, despite an upregulation of steady-state protein levels, translation of IRP2 was also inhibited (Fig. 6A, B), suggesting the involvement of a compensatory mechanism that regulates IRP2 levels. No clear effects on translation efficiency were observed for other mRNAs, nor for any mRNAs at 6 h of Eltrombopag treatment (Fig. 7 and Additional file 1: Fig. S10B). These results suggest that prolonged Eltrombopag treatment leads to global translation repression and a shift of HuR toward lighter polysomes (Fig. 7). However, the impact on translation of individual mRNAs appeared to be more specific, showing a degree of selectivity.

We next investigated whether Eltrombopag influences proteasomal degradation of specific proteins. WT HeLa cells treated with the proteasomal inhibitor MG132 showed elevated levels of FTL and FTH1 proteins, while IRP2 and CGA levels were significantly downregulated (Additional file 1: Fig. S11A, B). These findings confirm that FTL and FTH1 undergo canonical proteasomal degradation, whereas IRP2 and CGA may be subject to downregulation through secondary effects on overall cell fitness, like those observed for CDK2, Bcl-2, or IκBα [28–30]. Importantly, co-treatment with Eltrombopag and MG132 resulted in a less pronounced reduction in FTL and FTH1 levels compared to Eltrombopag treatment alone, indicating that the proteasomal pathway plays a role in Eltrombopag’s mechanism of action (Additional file 1: Fig. S11A, B). This effect was not observed for IRP2 and CGA, suggesting that Eltrombopag employs diverse targeting mechanisms.

Overall, these results highlight the multifaceted activity of Eltrombopag in regulating translation and protein stability, with at least part of its effects mediated by HuR.

### Genetic ablation and pharmacological inhibition of HuR regulate iron, lipid peroxidataion, and ROS levels

**Fig. 8:**
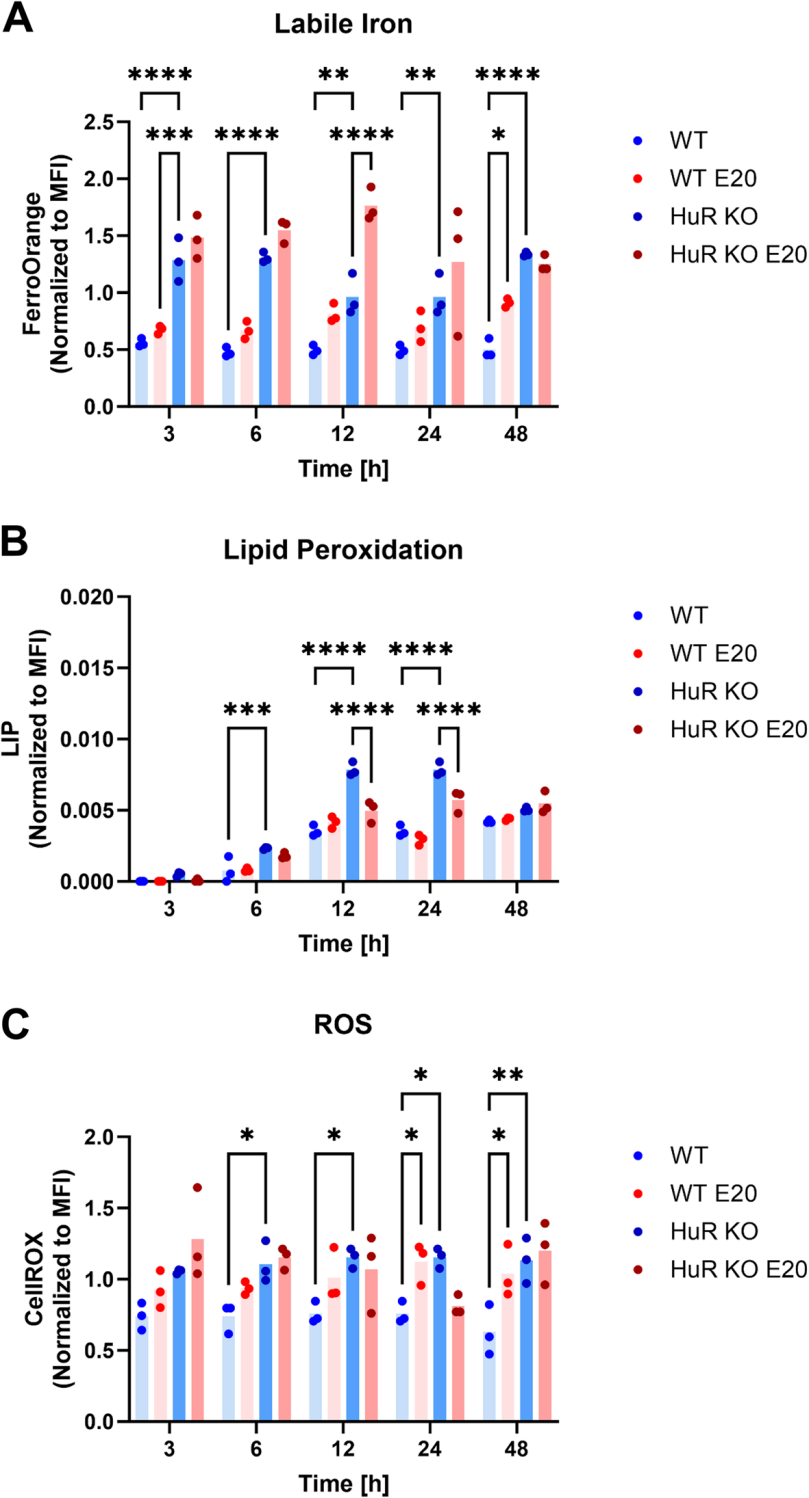
The treatment with Eltrombopag shows alterations in the iron and lipid peroxidation levels. WT and HuR KO cells were subjected to Eltrombopag (E20) treatment or DMSO as a control and analyzed at 3, 6, 12, 24, and 48 h. **A** Cytosolic ferrous iron (Fe.^2+^) levels were quantified using FerroOrange with flow cytometry. **B** Lipid peroxidation was assessed using the Lipid Peroxidation Assay Kit with flow cytometry. **C** Cytosolic ROS levels were evaluated by measuring CellROX Deep Red fluorescence intensity with flow cytometry. Data are presented as the change in the mean fluorescence intensity (MFI) ± standard deviation (SD) (*n* = 3). Statistical significance is denoted by * for *p* < 0.05, ** for *p* < 0.01, *** for *p* < 0.001, and **** for *p* < 0.0001. Two-way ANOVA was employed, and statistical significance was measured with Šidak adjustment for multiple comparisons. Individual data values are presented in Additional file 2

Next, we aimed to assess the impact of Eltrombopag and HuR KO on cellular iron balance and its consequences. We measured intracellular labile iron levels (by FerroOrange probe), oxidative stress (by CellROX assay), and lipid peroxidation over 48 h of time-course treatment with Eltrombopag in WT and HuR KO cells. Across all time points, increased labile iron was evident in HuR KO cells compared to WT cells, indicating iron accumulation in the absence of HuR (Fig. 8A). Treatment with Eltrombopag further augmented FerroOrange intensity at 12 h in both WT and HuR KO cells, implying exacerbation of iron overload. However, at some time points (e.g., 3, 24, and 48 h), the effects of Eltrombopag were not observed in HuR KO cells. Thus, while HuR KO is linked to heightened levels of labile iron, the influence of Eltrombopag treatment on iron levels extends beyond HuR regulation. This implies that Eltrombopag affects additional targets independent of HuR, which influence iron metabolism. Lipid peroxidation measurements similarly revealed more pronounced oxidative damage in HuR KO cells compared to WT cells (Fig. 8B). Notably, Eltrombopag specifically decreased lipid peroxidation levels at 12–24 h in HuR KO cells. In contrast, Eltrombopag had no impact on lipid peroxidation in WT cells (Fig. 8B). This suggests that when HuR is absent, the cellular landscape allows for Eltrombopag-induced remodeling of lipid metabolism. Finally, CellROX staining analysis revealed higher baseline ROS levels in HuR KO cells compared to WT cells (Fig. 8C), along with significant changes in ROS levels after 24 h of treatment. Interestingly, at this point, Eltrombopag treatment increased ROS levels in WT cells while decreasing them in HuR KO cells.

In summary, our findings suggest that HuR KO and Eltrombopag treatment led to increased labile iron levels and heightened oxidative damage. Further studies are needed to elucidate the exact mechanisms linking HuR regulation, Eltrombopag, and cellular metabolism.

## Discussion

Eltrombopag is an oral, small molecule thrombopoietin receptor agonist that promotes megakaryocyte growth and platelet production. It is clinically utilized for thrombocytopenia in disorders such as ITP and aplastic anemia [31]. A growing body of evidence demonstrates that Eltrombopag displays many effects ranging from native thrombopoietin agonism to immunomodulation, anti-inflammatory, and metabolic properties [20, 32, 33]. Recently, the RNA-binding protein HuR has emerged as a key mediator of Eltrombopag’s mechanism of action [20, 21]. Moreover, the transcription factor EB (TFEB) has been detected as an Eltrombopag target in starvation-induced conditions [34]. Our study analyzed the impact of Eltrombopag on RNA–protein interactions, the cellular transcriptome, and the proteome, with a specific focus on HuR, offering a comprehensive understanding of its influence on cellular processes through a multifaceted analysis.

The dysregulation of HuR has been linked to numerous disorders, highlighting its potential as a therapeutic target. Consequently, small molecules that disrupt HuR-RNA interactions could prove invaluable in modulating HuR functions in human diseases. For instance, cryptotanshinone inhibits HuR’s binding to tumor necrosis factor alpha (TNF-α) mRNA and blocks its nuclear-cytoplasmic translocation in macrophages [35]. Similarly, compounds like pyrvinium pamoate [36] and N-benzylcantharidinamide [37] prevent HuR activation through signaling pathways affecting its subcellular localization. Additionally, MPT0B098, a microtubule inhibitor, significantly reduces HuR translocation from the nucleus to the cytoplasm in various tumor cells, leading to decreased hypoxia-inducible factor 1-α (HIF-1-α) protein expression [38]. Inhibiting HuR can enhance apoptosis and anti-inflammatory responses in chronic lymphocytic leukemia (CLL) cells, rendering them more sensitive to chemotherapy and other treatments [39]. Our findings suggest that Eltrombopag holds promise as an anti-HuR agent, offering a direct avenue for repurposing in diverse diseases.

Our investigation aimed to reveal the molecular effects of genetic ablation and pharmacological inhibition of HuR. Using a sensitive RP-CONA assay [20], Eltrombopag was found to directly inhibit HuR binding to pri-miR-7 transcript, exhibiting low μM IC_50_ values. Previous reports indicated that HuR inhibits miR-7 levels by binding to pri-miR-7 RNA [23, 40] and that targeting HuR with naturally occurring flavonoid—quercetin increases miR-7 levels [22]. Surprisingly, we have detected downregulation of miR-7 after Eltrombopag treatment (Additional file 1: Fig. S12). This may be attributed to the actions of other RNA-binding proteins that regulate miR-7 biogenesis [41] or other HuR-unrelated events, triggered by Eltrombopag, that shape miR-7 levels. Using HuR KO cells and Eltrombopag treatment, we assessed the transcriptome and the proteome in human cultured cells. Our RNAseq analysis revealed 748 differentially expressed genes in wild-type cells treated with Eltrombopag, with enrichment observed in cancer pathways. Comparison with HuR KO cells treated with Eltrombopag showed partial overlap, indicating Eltrombopag broadly alters the transcriptome in an HuR-independent manner. In contrast, quantitative proteomics analysis by MS identified 137 differentially expressed proteins with Eltrombopag treatment in wild-type cells, but only 18 proteins were altered in HuR KO cells. This discrepancy underscores HuR critical role in mediating the downstream proteomic effects of Eltrombopag. Interestingly, we observed global translation inhibition following prolonged treatment with Eltrombopag, which could, over time, lead to more substantial effects on the overall levels of numerous proteins. These findings align with previous research emphasizing HuR role in regulating gene expression, particularly in cancer progression, positioning it as an attractive target for therapeutic interventions [42].

Integrated analysis of multi-omics datasets showed a subset of iron regulatory proteins modulated by Eltrombopag in a HuR-dependent manner at the posttranscriptional level, disrupting iron homeostasis and causing oxidative stress [43]. This network includes key players such as FTH1 and FTL, proteins critical for cellular iron homeostasis which store iron in redox-inert form and on-demand undergo a process termed ferritinophagy to release iron in the cytoplasm [44]. In our experiments, Eltrombopag treatment led to significant reductions in the levels of FTH1 and FTL while concurrently upregulating IRP2, a vital regulator of cellular iron metabolism. IRP2 is known to bind to hairpin-like structures called IREs in 5′UTR of target mRNAs such as FTH1 and FTL [24, 45] thereby potentially regulating their translation. Indeed, we did not observe a significant change in the translation efficiency of FTH1 and FTL upon Eltrombopag treatment, indicating the involvement of secondary effects. Notably, HuR has been shown to bind to the 3′UTRs of certain mRNAs, such as c-myc, repressing their expression by recruiting let-7/RISC [46]. A similar mechanism is likely at play for the IRP2 transcript. Our RIP analysis confirmed that HuR binds to IREB2 mRNA (which codes for IRP2) and demonstrated that Eltrombopag inhibits this interaction. We also found that treatment with MG132 reduced the levels of IRP2. Previous studies have demonstrated that IRP2 levels decline in response to H_2_O_2_ administration in SH-SY5Y cells [47]. Given that MG132 is known to induce the production of ROS, including H_2_O_2_ [48], this observation may suggest a link between MG132-induced ROS generation and the reduction in IRP2 levels. In conclusion, we propose that Eltrombopag disrupts iron homeostasis and induces oxidative stress by modulating key iron regulatory proteins, including FTH1, FTL, and IRP2, through HuR-dependent posttranscriptional mechanisms.

Recently, HuR was shown to play a crucial role in the systematic regulation of iron homeostasis by binding to 3′UTR of hepcidin mRNA in the liver, thereby stabilizing its transcript and increasing hepcidin expression [49, 50]. This could subsequently lead to the suppression of ferroportin-mediated iron export and hence cellular iron retention, which in turn would require enhanced ferritin expression [51]. Such a scenario would be consistent with the dual effect of HuR on iron regulatory proteins at both systemic and cellular levels and suggest a complex interplay orchestrated by Eltrombopag, highlighting its multifaceted impact on cellular iron handling pathways [52]. Finally, since the induction of rapid iron accumulation and ferroptosis could be exploited in anti-cancer treatment, our findings may inspire the growing spectrum of potential therapeutic effects of Eltrombopag.

Our study revealed that Eltrombopag decreased levels of CCND1 in WT but not in HuR KO cells, potentially influencing cell cycle progression [53, 54]. This was reflected in our findings, which demonstrated a reduction in cell proliferation following Eltrombopag treatment. CCND1 is known to play a critical role in cell cycle regulation and has been implicated in influencing sensitivity to ferroptosis, a form of regulated cell death associated with iron-dependent oxidative stress [54]. By modulating CCND1 levels, Eltrombopag may additionally exert indirect effects on ferroptosis sensitivity, further underscoring its potential role in influencing cell fate decisions. Similarly, Eltrombopag treatment reduced levels of glycoprotein hormone CGA in WT cells but not in HuR KO at both mRNA and protein levels [55]. The levels of CGA mRNA and protein were reduced dramatically in HuR KO cells. CGA has been linked to various hormone-related pathways implicated in cancer progression, including those involving EGFR and other oncogenic signaling cascades such as ERK 1/2 and Akt signaling [55]. Crucially, RIP analysis revealed that both CCND1 and CGA mRNAs interact with HuR. Notably, Eltrombopag completely abolished the binding between CGA mRNA and HuR, whereas its effect on the strong interaction between CCND1 mRNA and HuR was evident but did not reach statistical significance. These findings suggest that Eltrombopag may play a role in regulating cell cycle and hormone-related pathways linked to cancer progression, primarily through HuR-dependent mechanisms.

The primary limitation of this study is its focus on a single type of cell line. Future research should aim to expand these experiments to include other established and primary cell lines and even whole animal models. Moreover, it would be valuable to compare the effects of Eltrombopag with various other HuR-targeting compounds, both currently available and those that may be developed in the future. Finally, to fully explore the therapeutic potential of targeting HuR, it will be crucial to investigate the long-term effects on cellular fitness, as well as the transcriptomic and proteomic profiles of cells treated with anti-HuR compounds.

## Conclusions

This study sheds light on the complex effects of both genetic ablation and pharmacological inhibition of the RNA-binding protein HuR. Eltrombopag, a thrombopoietin receptor agonist, was shown to influence RNA–protein interactions, the transcriptome, and the proteome, with a significant focus on HuR. The findings reveal that Eltrombopag disrupts HuR binding to specific RNA targets, thereby altering posttranscriptional regulation of proteins critical for iron metabolism, cell cycle control, and hormone signaling. Notably, while many of Eltrombopag’s effects were found to be HuR-dependent, others occurred independently, pointing to various mechanisms of action. These results emphasize the therapeutic promise of Eltrombopag as an anti-HuR agent and underscore its potential for targeting HuR-regulated pathways in cancer and other diseases.

## Methods

### Cell culture and Eltrombopag treatment

HuR knockout (KO) HeLa cell lines were previously generated using CRISPR-Cas9 as described [22]. Wild-type and HuR KO HeLa cells were cultured in Dulbecco’s Modified Eagle’s Medium (DMEM) + GlutaMAX with 10% fetal bovine serum (FBS) (Gibco). Recombinant HuR with mCherry protein was expressed as reported previously [22]. Eltrombopag was purchased from Selleckchem (catalogue number S4502). The viability of HeLa wild-type (WT) and HuR knockout (KO) cells treated with 20 µM of Eltrombopag or DMSO was assessed using CellTiterGlo at various time points (0 h, 3 h, 6 h, 12 h, 24 h, and 48 h) using CellTiter-Glo 3D Viability Assay, following the manufacturer’s protocol.

### RNA pull-down confocal nanoscanning (RP-CONA)

The interaction between HuR and DHX9 protein and Eltrombopag was evaluated by RNA pull-down confocal nanoscanning (RP-CONA) assay as described before [22]. Briefly, a 5′ 6-FAM- and 3′ biotin-labeled RNA pri-miR-7 conserved terminal loop (CTL) transcript (pri-miR-7–6-FAM) (IDT) was immobilized on streptavidin-coated Ni–NTA agarose beads (ProteoGenix). Extracts from HuR KO HeLa cells expressing mCherry-HuR were incubated with RNA-bound beads and various concentrations of Eltrombopag (0, 10, 20, 30, 40, and 50 μM). After incubation, beads were imaged by confocal microscopy (Opera Phoenix) to quantify mCherry-HuR recruitment to fluorescently labeled RNA. The RP-CONA between mCherry-DHX9 from HeLa extracts and pri-miR-7–6-FAM with DMSO or Eltrombopag (20 μM) was used as a negative control. Data were obtained from three technical replicates and mean was calculated. For fluorescence anisotropy, the IC_50_ was calculated using nonlinear regression (4PL, GraphPad Prism Software 10.0.2, *R*_adj_^2^ = 0.98).

### RNA extraction and quality control

Cells were seeded in 6-well plates and treated with DMSO (control) and Eltrombopag (20 μM). After 48 h, total RNA was extracted from two replicates per treatment using TRIzol reagent (Life Technologies, CA, USA). RNA concentration was quantified using Qubit 2.0 Fluorometer (Invitrogen, Carlsbad, CA, USA) and RNA integrity was checked using Agilent 5200 Fragment Analyzer (Agilent Technologies, Palo Alto, CA, USA). The samples with RNA integrity number (RIN) ≥ 9 were subjected to the subsequent analysis. Initially we prepared two set of samples for RNAseq analysis (batch 1) followed by an additional sample set (batch 2).

### RNA sequencing and data analysis

A total of 2 μg of total RNA per sample was used as input material for the RNA library. Sequencing libraries were generated using standard library for IlluminaR (NEB, USA) following the manufacturer’s recommendations.

Samples were sequenced on Illumina NovaSeq 6000 to generate 150 nucleotide reads with paired end. Raw sequence data was converted into fastq files and de-multiplexed using bcl2fastq 2.20, allowing for one mismatch in index sequence identification. Quality control of raw reads was performed using fastqc, and reads were trimmed using BBDuk. Cleaned paired-end reads were then mapped to the human reference genome using the STAR aligner v.2.5.2b [56]. Gene quantification was performed on the resulting BAM files using Salmon with the help of genome index build based on GENCODE release 42 to obtain unique gene counts. Differential gene expression analysis was performed between treatment groups using DESeq2 [57]. Genes with log_2_FC ± 1 and adjusted *p* values < 0.05 were considered as statistically significant for each comparison. Gene-set enrichment analysis on differently expressed genes were performed using ShinyGO 0.80 [58]; the data visualization was done using ggplot2 package. Volcano plots, principal component analysis (PCA), sample-to-sample distances, and Euler diagrams were performed to demonstrate changes and patterns within each comparison among all samples using in-house R and Python scripts. AREs were downloaded from the ARED-plus and compared with ENSEMBL ID of DE genes [59].

### Mass spectrometry

Cells were seeded in 6-well plates and incubated overnight to adhere. The next day, they were treated with DMSO (control) and Eltrombopag (20 µM). After 48 h, protein from 3 replicates have been collected. Protein was extracted using Roeder D (no glycerol), regular sonication cycle. Protein samples from all biological replicates were processed at the same time and with using the same digestion protocol without any deviations. They were subjected for MS analysis under the same conditions. Protein and peptide lists generated using the same software and the same parameters. Specifically, 5 µg of total protein from each sample were digested using the Filter Aided Sample Preparation (FASP) protocol as described by Wisniewski et al. with minor modifications [60]. In brief, each protein sample was added on the top of a 30 kDa MWCO filter units (Vivacon, UK) along with 150 µl of denaturation buffer (8 M urea in 50 mM ammonium bicarbonate (ABC) (Sigma Aldrich)) and spun at 14,000 × g for 20 min, while another wash with 200 µl of denaturation buffer was performed under the same conditions. The protein samples were then reduced by the addition of 100 µl of 10 mM dithiothreitol (Sigma Aldrich, UK) in denaturation buffer for 30 min at ambient temperature, and alkylated by adding 100 µl of 55 mM iodoacetamide (Sigma Aldrich, UK) in denaturation buffer for 20 min at ambient temperature in the dark. Two washes with 100 µl of denaturation buffer and two with digestion buffer (50 mM ABC) were performed under the same conditions described above before the addition of trypsin (Pierce, UK). The protease:protein ratio was 1:50 and proteins were digested overnight at 37 °C. Following digestion, samples were spun at 14,000 × g for 20 min and the flow-through containing digested peptides was collected. Filters were then washed one more time with 100 µl of digestion buffer and the flow-through was collected again. The eluates from the filter units were acidified using 20 µl of 10% trifluoroacetic acid (TFA) (Sigma Aldrich) and spun onto StageTips as described before [61]. Peptides were eluted in 40 μl of 80% acetonitrile in 0.1% TFA and concentrated down to 1 μl by vacuum centrifugation (Concentrator 5301, Eppendorf, UK). The peptide sample was then prepared for LC–MS/MS analysis by diluting it to 5 μl by 0.1% TFA.

LC–MS analyses were performed on an Orbitrap Exploris™ 480 Mass Spectrometer (Thermo Fisher Scientific, UK) coupled on-line to an Ultimate 3000 HPLC (Dionex, Thermo Fisher Scientific, UK). Peptides were separated on a 50 cm (2 µm particle size) EASY-Spray column (Thermo Scientific, UK), which was assembled on an EASY-Spray source (Thermo Scientific, UK) and operated constantly at 50 °C. Mobile phase A consisted of 0.1% formic acid in LC–MS grade water and mobile phase B consisted of 80% acetonitrile and 0.1% formic acid. Peptides were loaded onto the column at a flow rate of 0.3 μl min^−1^ and eluted at a flow rate of 0.25 μl min^−1^ according to the following gradient: 2 to 40% mobile phase B in 180 min and then to 95% in 11 min. Mobile phase B was retained at 95% for 5 min and returned back to 2% a minute after until the end of the run (220 min).

Survey scans were recorded at 120,000 resolution (scan range 350–1650 m/z) with an ion target of 5.0e6 and injection time of 20 ms. MS2 Data Independent Acquisition (DIA) was performed in the Orbitrap at 30,000 resolutions with a scan range of 200–2000 m/z, maximum injection time of 55 ms, and AGC target of 3.0E6 ions. We used HCD fragmentation [62] with stepped collision energy of 25.5, 27, and 30. The inclusion mass list with the correspondent isolation windows are shown in the table below. Data for both survey and MS/MS scans were acquired in profile mode.

MS data were searched against the UniProt human database using MaxQuant v1.6.6 [63]. Label-free quantification was performed using the MaxLFQ algorithm [64] integrated into MaxQuant. Differential gene expression analysis was performed using DEqMS with cut offs (log2FC ± 1 and padj < 0.05) [65]. Insignificant proteins of HuR KO treatment were correlated and cross compared with knockout effect and WT treatment effect.

### qRT-PCR

Total RNA was extracted from HeLa cells (wild type, WT-E20, HuR KO, HuR KO E20) using the InviGen Invisorb spin virus RNA mini kit (Invitek) following the manufacturer’s protocol. RNA concentration and purity were measured using a NanoDrop 2000 spectrophotometer (Thermo Scientific). Total RNA was reverse transcribed into cDNA using the Promega 1 Step qRT-PCR kit (Promega) per the manufacturer’s instructions. Twenty-five-microliter reactions were prepared and qRT-PCR was performed using the QuantStudio 5 Real-Time PCR System (Applied Biosystems). Three biological replicates and three technical replicates were analyzed for each sample. QuantStudio Design and Analysis Software v1.5.2 (Applied Biosystems) was used to determine Cq values. Relative quantification of gene expression was performed using the comparative CT method (ΔΔCT method) in R programming language. GAPDH and ACTNB were used as reference genes for normalization. Throughout our study, statistical significance was determined by Student *t*-test comparing WT and treatment groups using R. *p* < 0.05 was considered statistically significant (*p < 0.05, **p < 0.01, ***p < 0.001). Data are denoted as mean ± SEM. The data are presented as mean ± S.D., with *n* ≥ 3 per group per treatment for all studies. One-way ANOVA was implemented to find statistical significance between scramble Eltrombopag treated against HuR KD Eltrombopag treated cells. The primer sequences are listed in Additional file 1: Table S2.

### miR-7 measurement

HeLa WT cells were treated with DMSO or 20 µM Eltrombopag, and total RNA was collected at multiple time points (0, 3, 6, 12, 24, and 48 h) using the Total RNA Zol-Out D kit. RNA was reverse transcribed into cDNA using the microScript microRNA cDNA Synthesis Kit (Cat# 54,410). Equal amounts of cDNA were subjected to qPCR, using the Promega 1-Step qRT-PCR kit (Promega) per the manufacturer’s instructions. Three biological replicates and three technical replicates were analyzed for each sample. QuantStudio Design and Analysis Software v1.5.2 (Applied Biosystems) was used to determine Cq values. Relative quantification of gene expression was performed using the comparative CT method (ΔΔCT method).

### Western blot analysis

Cultured cells were washed once with PBS and lysed in RIPA buffer (1% Triton X-100, 0.5% sodium deoxycholate, 0.1% SDS, 50 mM Tris pH 7.4, 150 mM NaCl, 0.5 mM EDTA) supplemented with 100 × Protease Inhibitor Cocktail (P8340; Sigma-Aldrich) and Phosphatase Inhibitor Cocktail 2 (P5726; Sigma-Aldrich) and 3 (P0044; Sigma-Aldrich). After centrifugation (13,000 rpm, 15 min, 4 °C), the supernatants were collected. Protein concentration was determined with BCA Protein Assay Kit (23,225; Thermo Fisher Scientific). Thirty micrograms of total protein per sample was mixed with 6 μl of 5 × SB buffer (250 mM Tris–HCl, 10% SDS, 50% glycerol, 250 mM DTT, 0.02% bromophenol blue), loaded on 12% SDS PAGE and transferred onto a nitrocellulose membrane. Membranes were blocked for 1 h with Western Blocking Reagent (Roche), 5% milk or 5% BSA (Sigma) in TBST and incubated overnight at 4 °C with specific primary antibodies (Additional file 1: Table S3). After 1-h incubation with horseradish peroxidase-conjugated secondary antibody (P044801-2; Agilent), the signal was detected with Clarity Western ECL Substrate (179–5061; Bio-Rad) and visualized with ChemiDoc imaging system (Bio-Rad). Densitometric analysis was performed using Image Lab software (Bio-Rad).

### Fluorescence anisotropy

Fluoresce anisotropy experiments were carried out in a buffer containing phosphate buffer pH = 7.5, 25 mM MgCl, 250 mM NaCl, 0.1% Tween 20, and 5 mM DTT. The concentration of pri-miR-7–1–6-FAM CTL (IDT) was 20 nM, Recombinant Human Elav-like protein 1 (ELAVL1/HuR) (CusaBio) was 500 nM, and concentrations of Eltrombopag were 0, 0.625, 1.25, 2.5, 5, 10, 20, 40, 60, 80, and 100 µM. Samples were prepared in triplicates in a black 384-well assay plate with round bottom. Fluorescence anisotropy was measured using Tecan INFINITE M1000 operated by Magellan software. Excitation and emission wavelengths were 495 nm and 520 nm. IC_50_ of Eltrombopag was calculated with a non-linear regression model using GraphPad Prism Software 10.0.2.

### RNAi

HeLa cells were seeded at a density of 300,000 cells per well in 6-well plates, allowing them to reach about 60% confluency. The cells were left overnight to attach under standard culture conditions. The next day, they were gently washed once with fresh growth media and then with Opti-MEM (Thermo Fisher Scientific). Afterward, 1.5 ml of DMEM was added to each well. For transfection, a mixture of lipofectamine and siRNA (anti-HuR or scramble control) was prepared according to the manufacturer’s protocol (RNAiMAX, Invitrogen) and added to the cells in a total volume of 0.5 ml, with a final siRNA concentration of 100 pmol per well. The siRNA was sourced from Horizon Discovery. After 24 h of transfection, the media was removed, and the cells were washed with fresh media. The cells were then treated with 20 µM Eltrombopag (Selleckchem), while DMSO served as the negative control. After 48 h of treatment, the cells were collected for analysis. RNA samples were extracted using TRIzol reagent (Thermo Fisher Scientific) from wells dedicated to qRT-PCR, while protein samples were prepared by lysing cells with RIPA buffer containing protease and phosphatase inhibitors (Thermo Fisher Scientific) for western blot analysis.

### Polysome profiling

Polysome fractionation was performed as described in the protocol [66]. Briefly, 48 h prior to fractionation, cells were treated with either DMSO (control) or 20 μM Eltrombopag. The day before cell lysis, 10–50% sucrose gradients were prepared in GB buffer (10 mM HEPES–KOH pH 7.2, 150 mM KCl, 5 mM MgCl2, Protease Inhibitor Cocktail (Roche), 100 µg/ml cycloheximide—CHX (J66901.03, Thermo Fisher Scientific), 4 U/ml RiboLock (EO0382, Thermo Fisher Scientific), and nuclease-free water). On the day of lysis, cells were treated with 100 µg/ml CHX, and 15 × 10^6 cells were lysed in GB buffer supplemented with 1% NP40 for 10 min on ice. After centrifugation at 14,000 rpm for 5 min at 4 °C, the protein concentration in the supernatants was measured using a NanoDrop spectrophotometer. Equal amounts of proteins were loaded onto the top of the sucrose gradients, and the gradients were subjected to ultracentrifugation at 38,000 rpm for 2 h at 4 °C using an Optima XPN ultracentrifuge (RRID: SCR_018238) and an SW41Ti rotor (Beckman Coulter). Following centrifugation, sucrose fractions were collected using a Density Gradient Fractionation System (Teledyne ISCO) with a Foxy Jr. Fraction Collector. Fractions were stored at − 80 °C for subsequent analysis. During collection, absorbance at 254 nm was monitored and recorded to generate RNA distribution profiles, which were printed on ISCO paper, scanned, and digitized using PlotDigitizer (https://plotdigitizer.com). Graph generation was performed using GraphPad Prism 10.

For HuR protein distribution in the sucrose fractions, 26 µl of each fraction was mixed with 4 µl of 5 × SB buffer, boiled, and subjected to SDS-PAGE. Western blot analysis was then performed, and signal intensities were quantified via densitometry. The total signal intensity across all fractions was normalized to 100%, and the percentage of signal intensity for each fraction was calculated.

For mRNA distribution analysis, 50 µl from each fraction was pooled as depicted in Additional file 1: Fig. S10A, mixed with 800 µl of TRIzol reagent and supplemented with 2 µl of TATAA Universal RNA Spike II (RS25SII, TATAA Biocenter). RNA was extracted according to the manufacturer’s protocol and purified using columns (A&A Biotechnology, 031–100). RNA was eluted with equal volumes of water from each column, and RNA purity was assessed using a NanoDrop spectrophotometer. qRT-PCR analysis was performed to quantify mRNA abundance in each fraction, and the percent distribution of mRNA across the gradients was calculated using the ΔΔCT method, with normalization to TATAA Universal RNA Spike values.

### HuR ribonucleoprotein immunoprecipitation (RIP)

RIP analysis of HuR protein was performed according to the protocol described [67]. Briefly, 25 µl of Protein G Dynabeads (Invitrogen, 10003D) was incubated with 2.5 µg of mouse monoclonal anti-HuR antibody (Santa Cruz Biotechnology, sc-5261) or normal mouse IgG1 isotype antibody (Santa Cruz Biotechnology, sc-3877) for 1 h at room temperature (RT) in NT2 buffer (50 mM Tris–HCl, pH 7.5, 150 mM NaCl, 1 mM MgCl2, 0.05% NP-40). After 48 h of treatment with DMSO (control) or 20 µM Eltrombopag (E20), cells were lysed in PEB buffer (20 mM Tris–HCl, pH 7.5, 100 mM KCl, 5 mM MgCl2, 0.5% NP-40) supplemented with a Protease Inhibitor Cocktail and 40 U/ml of RiboLock. A total of 1000 µg of protein lysate was added to the antibody-coated bead complexes, and the immunoprecipitation (IP) reaction was carried out in NT2 buffer (supplemented with 10 mM DTT, 400 U/ml RiboLock, 15 mM EDTA, and 0.2% DMSO or 20 µM Eltrombopag) for 2 h at 4 °C and 30 min at RT with rotation. The RIP complexes were washed five times with 1 ml of ice-cold NT2 buffer containing 40 U/ml RiboLock. A 100 µl aliquot of the RIP-bead complexes from the final wash was taken for western blot analysis to verify the efficiency of the immunoprecipitation. Following the final wash, the RIP complexes were treated with DNase I (10 U/µl) in 100 µl of NT2 buffer for 10 min at 37 °C to degrade any genomic DNA. The complexes were then washed once more in NT2 buffer. To release HuR-bound RNAs, proteinase K treatment was performed in NT2 buffer supplemented with 0.5 µg/µl proteinase K (Thermo Fisher Scientific, EO0491) and 0.1% SDS. The supernatants containing the released RNAs were collected and mixed with TRIzol reagent for RNA extraction, following the manufacturer’s protocol. RNA was then purified using spin columns. RNA purity was assessed using a NanoDrop spectrophotometer. qRT-PCR was performed to quantify mRNA enrichment in the RIP samples. The results were calculated using the ΔΔCT method, with normalization to GAPDH mRNA.

### Proteasome inhibition

HeLa WT cells were treated with Mg132 [5 µM] (474,790 Sigma) and (or) Eltrombopag [20 µM] for 24 h. The protein levels were determined with western blot technique as previously described. Densitometric analysis was performed using Image Lab software (Bio-Rad).

### Functional assays and flow cytometry

Intracellular reactive oxygen species (ROS) levels were measured using CellROX Deep Red reagent (Invitrogen, C10422) according to manufacturer’s protocol. Cells were analyzed by flow cytometry on a CytoFLEX instrument (Beckman Coulter) using the APC channel. Intracellular ferrous iron (Fe2 +) was measured by FerroOrange staining (Dojindo, F374) and flow cytometry using the PE channel. Lipid peroxidation was assessed with the Lipid Peroxidation Assay Kit (Abcam, ab243377) per kit instructions. Oxidized lipid levels were detected by flow cytometry by comparing FITC and PE channel. In all experiments, cell viability was determined by staining with LIVE/DEAD Fixable Aqua or Violet dyes (Invitrogen, L34966/L34964). Data was acquired on CytoFLEX and analyzed using CytExpert software (Beckman Coulter). Geometric mean fluorescence intensity (MFI) was quantified for each probe. In our experiments, we adopted a comparative approach, using wild-type (WT) cells as the baseline control for the study. Specifically, WT cells were analyzed as controls for HuR KO cells to evaluate the effects of HuR deletion on the specified cellular responses. Additionally, HuR KO cells treated with Eltrombopag served as controls for WT cells exposed to Eltrombopag, enabling us to distinguish between HuR-dependent and HuR-independent effects of the treatment. Statistical significance for the HuR KO and Eltrombopag treatment factors was determined using a two-way ANOVA. For fluorescent probes, we built upon previously established and published methods, which we had successfully optimized and applied for flow cytometric measurements of ROS, lipid peroxides, and labile iron using the respective probes [68].

## Supplementary Information

Additional file 1: Fig. S1 Eltrombopag does not quench the mCherry signal. Fig. S2 Fluorescence anisotropy shows Eltrombopag efficient inhibition of HuR/RNA binding. Fig. S3 Eltrombopag reduces the viability of WT HeLa cells. Fig. S4 Combined PC analysis and sample-to-sample clustering show overlapping results between corresponding samples. Fig. S5 Top 20 enriched GO terms from gene ontology analysisfrom RNAseq. Fig. S6 Combined PC analysis and sample-to-sample clustering show overlapping results between corresponding samples. Fig. S7 GO terms derived from mass spectrometry analysis. Fig. S8 Eltrombopag treatment affects the protein levels after HuR RNAi. Fig. S9 Eltrombopag abolishes HuR binding to IREB2 and CGA mRNAs. Fig. S10 6 h-Eltrombopag treatment has no effect on translation efficiency. Fig. S11 Eltrombopag affects proteasome-dependent degradation. Fig. S12 Eltrombopag decreases the expression of mature miR-7 in a time-dependent manner. Table S1 HuR dependent targets. Table S2 List of primers sequences. Table S3 List with primary antibodies used for the experiments.

Additional file 2: Individual data values from the experiments.

Additional file 3: Uncropped images of the original western blots.

## Data Availability

All data generated or analyzed during this study are included in this published article, its supplementary information files, and publicly available repositories. The data presented in the study are deposited in the Sequence Read Archive repository, BioProject accession number PRJNA1086447. The mass spectrometry proteomics data have been deposited in the ProteomeXchange Consortium via the PRIDE [70] partner repository with the dataset identifier PXD050698. Individual data values are presented in Additional file 2. Images of the original, uncropped blots are presented in Additional file 3.

## Abbreviations

3′UTR: 3′ Untranslated region
ACSS1: Acyl-CoA synthetase short chain family member 1
ACTN2: Actin alpha 2
ARE: AU-rich elements
BLZF1: Basic leucine zipper nuclear factor 1
CCND1: Cyclin D1
CGA: Glycoprotein hormones, alpha polypeptide
CIP: Chemotherapy-induced thrombocytopenia
CLL: Chronic lymphocytic leukemia
CTL: Conserved terminal loop
DE: Differentially expressed
DL: Differential levels
DMEM: Dulbecco’s Modified Eagle’s Medium
DYPD: Dihydropyrimidine dehydrogenase
E20: Eltrombopag
EZH1: Enhancer of zeste 1 polycomb repressive complex 2 subunit
FBS: Fetal bovine serum
HIF-1-α: Hypoxia-inducible factor 1-α
HuR: Human antigen R
IREB2: Iron responsive element binding protein 2
IRP2: Iron-regulatory protein 2
ITP: Immune thrombocytopenic purpura
KD: Knock down
KO: Knock out
FTL: Ferritin light chain
FTH: Ferritin heavy chain
PC: Principal component analysis
RBP: RNA-binding protein
RIP: Ribonucleoprotein immunoprecipitation
ROS: Reactive oxygen species
RP-CONA: RNA pull-down confocal nanoscanning
SMAD3: Smad family member 3
TFEB: Transcription factor EB
TNF-α: Tumor necrosis factor alpha
TP53INP2: Tumor protein P53 inducible nuclear protein 1
WT: Wild type
