# Supplementary material for: Effects of genetic ablation and pharmacological inhibition of HuR on gene expression, iron metabolism, and hormone levels
Source: BMC Biol. 2025 Jan 23;23:24. doi: 10.1186/s12915-025-02131-z (PMC11756078; doi:10.1186/s12915-025-02131-z)
Supplement: Supplementary file 1 — Additional file 1: Fig. S1 Eltrombopag does not quench the mCherry signal. Fig. S2 Fluorescence anisotropy shows Eltrombopag efficient inhibition of HuR/RNA binding. Fig. S3 Eltrombopag reduces the viability of WT HeLa cells. Fig. S4 Combined PC analysis and sample-to-sample clustering show overlapping results between corresponding samples. Fig. S5 Top 20 enriched GO terms from gene ontology analysisfrom RNAseq. Fig. S6 Combined PC analysis and sample-to-sample clustering show overlapping results between corresponding samples. Fig. S7 GO terms derived from mass spectrometry analysis. Fig. S8 Eltrombopag treatment affects the protein levels after HuR RNAi. Fig. S9 Eltrombopag abolishes HuR binding to IREB2 and CGA mRNAs. Fig. S10 6 h-Eltrombopag treatment has no effect on translation efficiency. Fig. S11 Eltrombopag affects proteasome-dependent degradation. Fig. S12 Eltrombopag decreases the expression of mature miR-7 in a time-dependent manner. Table S1 HuR dependent targets. Table S2 List of primers sequences. Table S3 List with primary antibodies used for the experiments. [file 12915_2025_2131_MOESM1_ESM.pdf]

Additional File 1

Effects of genetic ablation and pharmacological inhibition of HuR on gene expression, iron metabolism and hormone levels.

Nathalie Idlin[1], Sivakumar Krishnamoorthy[1*], Magdalena Wolczyk[1], Mouad Fakhri[1], Michal Lechowski[1], Natalia Stec[1], Jacek Milek[2], Pratik Kumar Mandal[1], Jaroslaw Cendrowski[3], Magdalena Dziembowska[2], Katarzyna Mleczko-Sanecka[1], Christos Spanos[4], Juri Rappsilber[5], Gracjan Michlewski[1]*

* - co-first authors

[1] International Institute of Molecular and Cell Biology in Warsaw, Poland

[2] Department of Animal Physiology, Faculty of Biology, University of Warsaw, Warsaw, Poland

[3] Maria Sklodowska-Curie National Research Institute of Oncology in Warsaw, Poland

[4] The Wellcome Centre for Cell Biology, University of Edinburgh, Edinburgh, UK

[5] Department of Biotechnology, Technische Universität Berlin, Berlin, Germany

Correspondence should be addressed to G.M. (gmichlewski@iimcb.gov.pl)

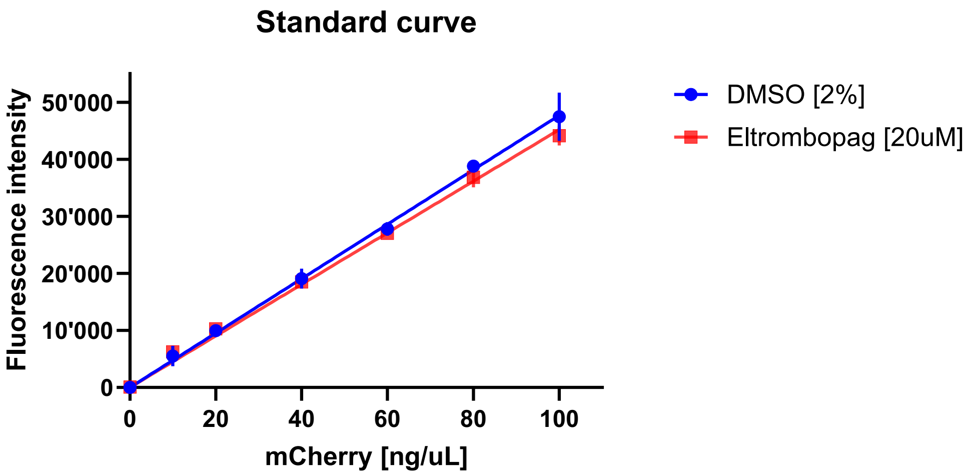

Supplementary Figure 1. Eltrombopag does not quench the mCherry signal. The fluorescent signal from mCherry protein was measured in various concentrations, with addition of DMSO or 20 μM Eltrombopag. The data are represented as three technical replicates (n=3) and shown as mean ± S.D. Individual data values are presented in Additional file 2.

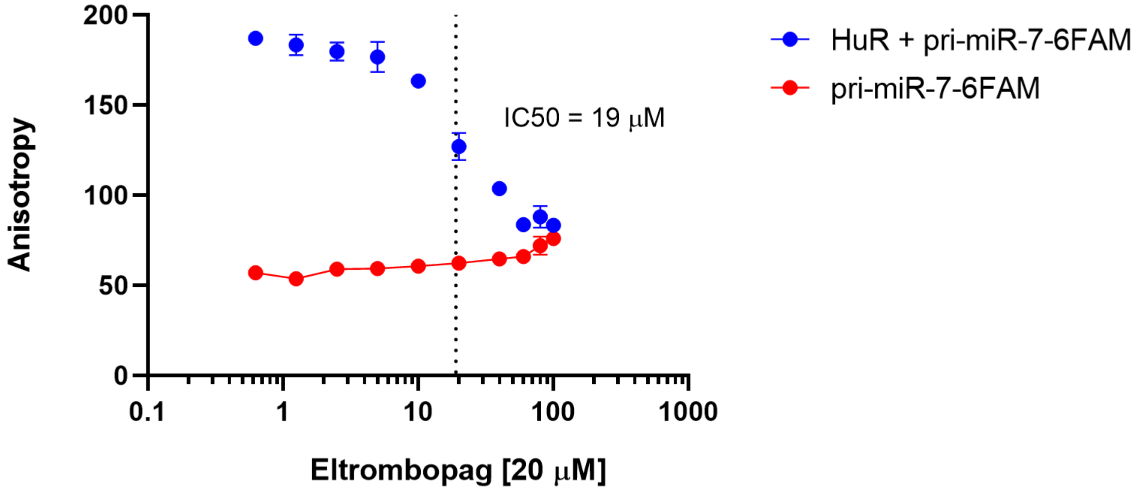

Supplementary Figure 2. Fluorescence anisotropy shows Eltrombopag efficient inhibition of HuR/RNA binding. 500 nM of purified recombinant HuR protein and 20 nM pri-miR-7-6FAM was used to assess the interaction with increasing concentration of Eltrombopag. The data are represented as three technical replicates (n=3) and shown as mean ± S.D. The IC_{50} was calculated using nonlinear regression (4PL, GraphPad Prism Software 10.0.2, R_{adj}^2=0.98). Individual data values are presented in Additional file 2.

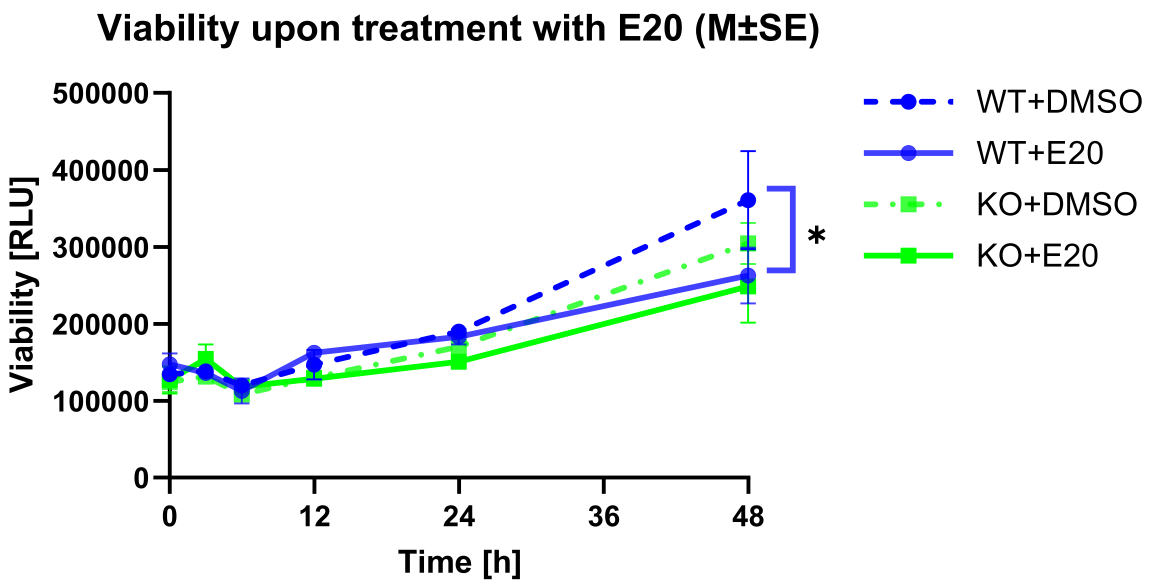

Supplementary Figure 3. Eltrombopag reduces the viability of WT HeLa cells. Viability of HeLa WT and HeLa HuR KO after 20 µM Eltrombopag or DMSO treatment at different timepoints (0-48 hours). The data are represented as mean ± S.E.; with n ≥ 4 per group per treatment for all sets. Statistical significance was determined using one-way ANOVA, with * denoting $p < 0.05$, indicating significant differences between WT DMSO and WT treatment. Individual data values are presented in Additional file 2.

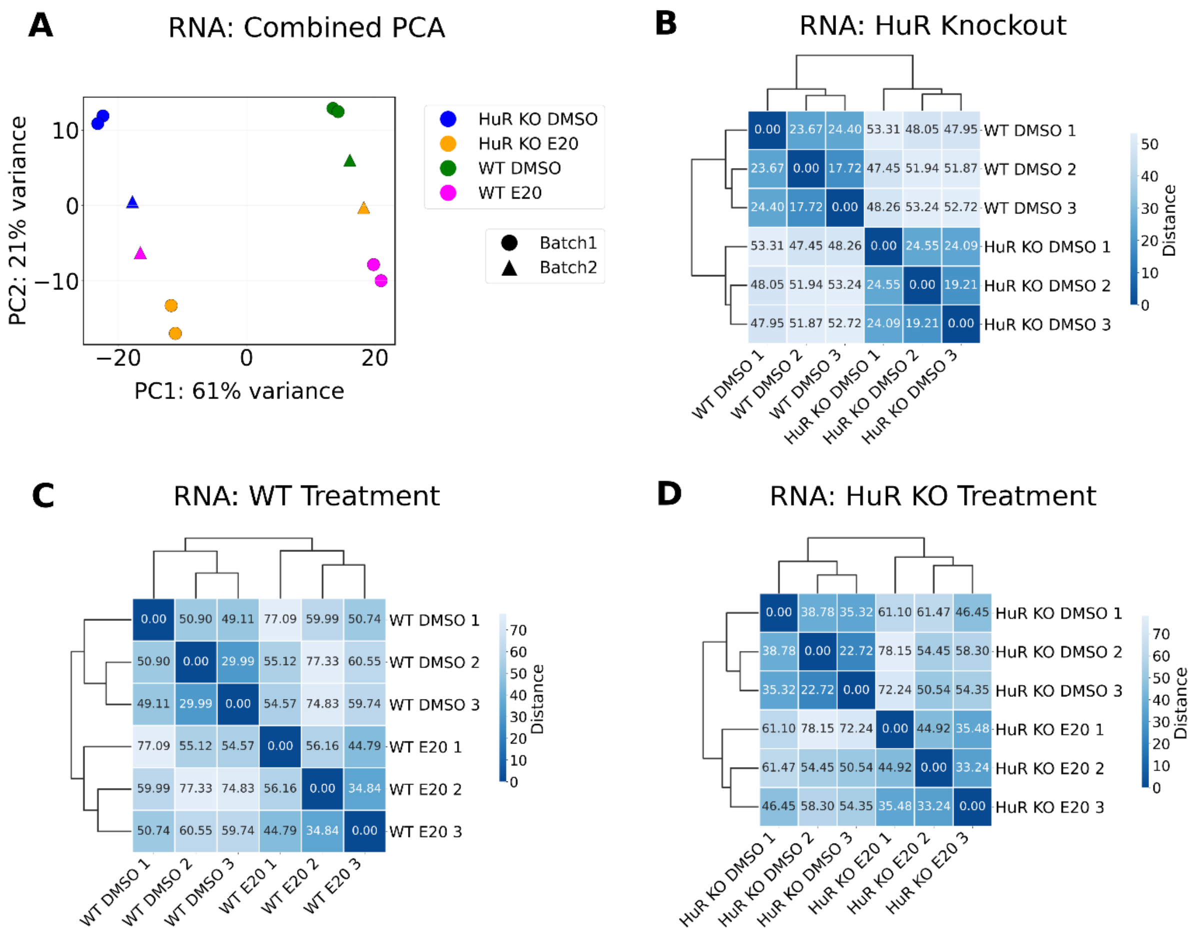

Supplementary Figure 4. Combined PC analysis and sample-to-sample clustering show overlapping results between corresponding samples. (A) Combined PC analysis from RNAseq. Samples 1 and 2 (circles) represent batch 1, and sample 3 (triangles) represent batch 2. (B-D) Sample-to-sample distance matrix clustering with calculated pairwise distances (B) HuR Knockout, (C) WT treatment, (D) HuR KO treatment.

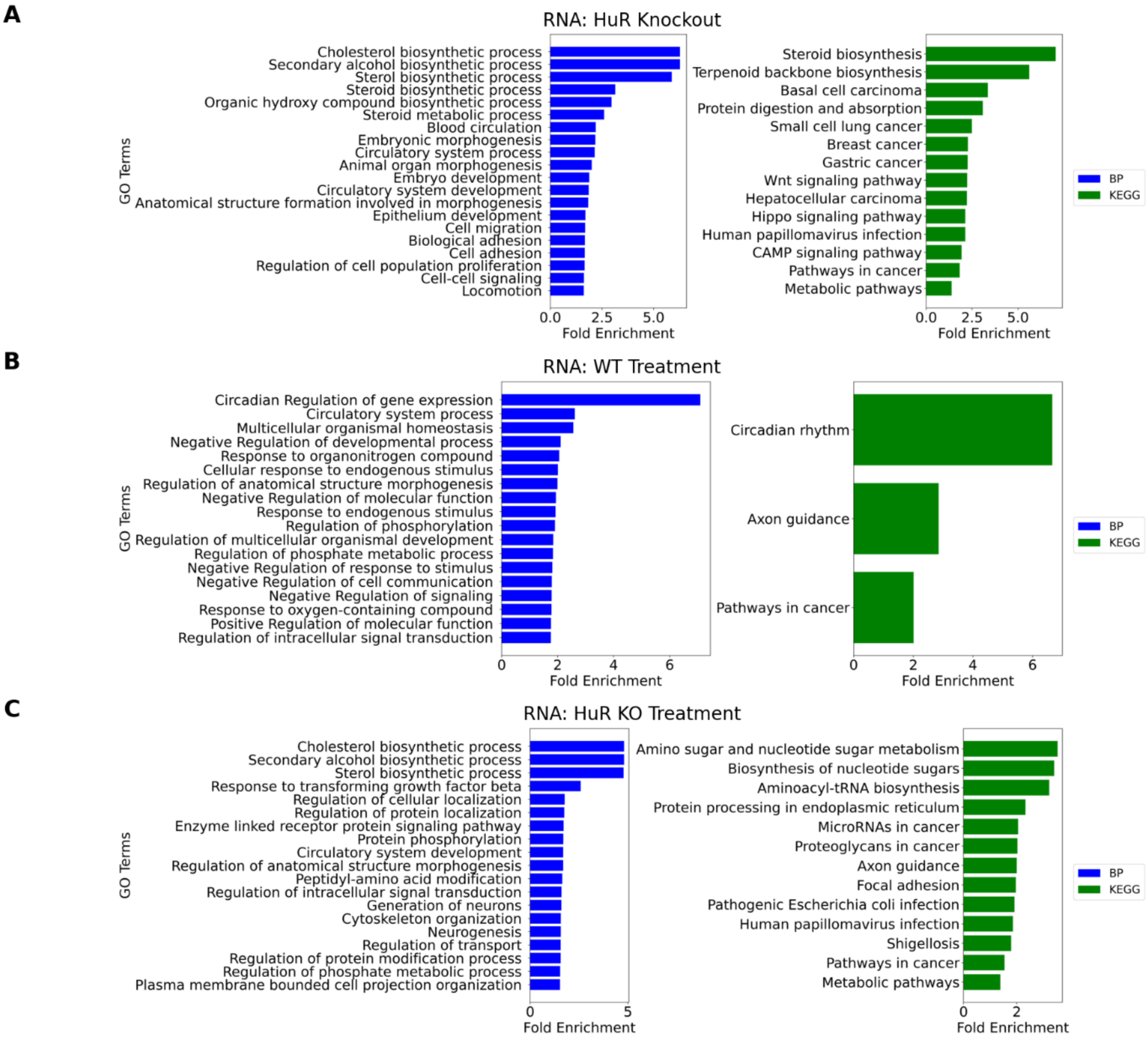

Supplementary Figure 5. Top 20 Enriched GO Terms from Gene Ontology analysis (BP, KEGG) from RNAseq (A) HuR Knockout, (B) WT treatment, (C) HuR KO treatment.

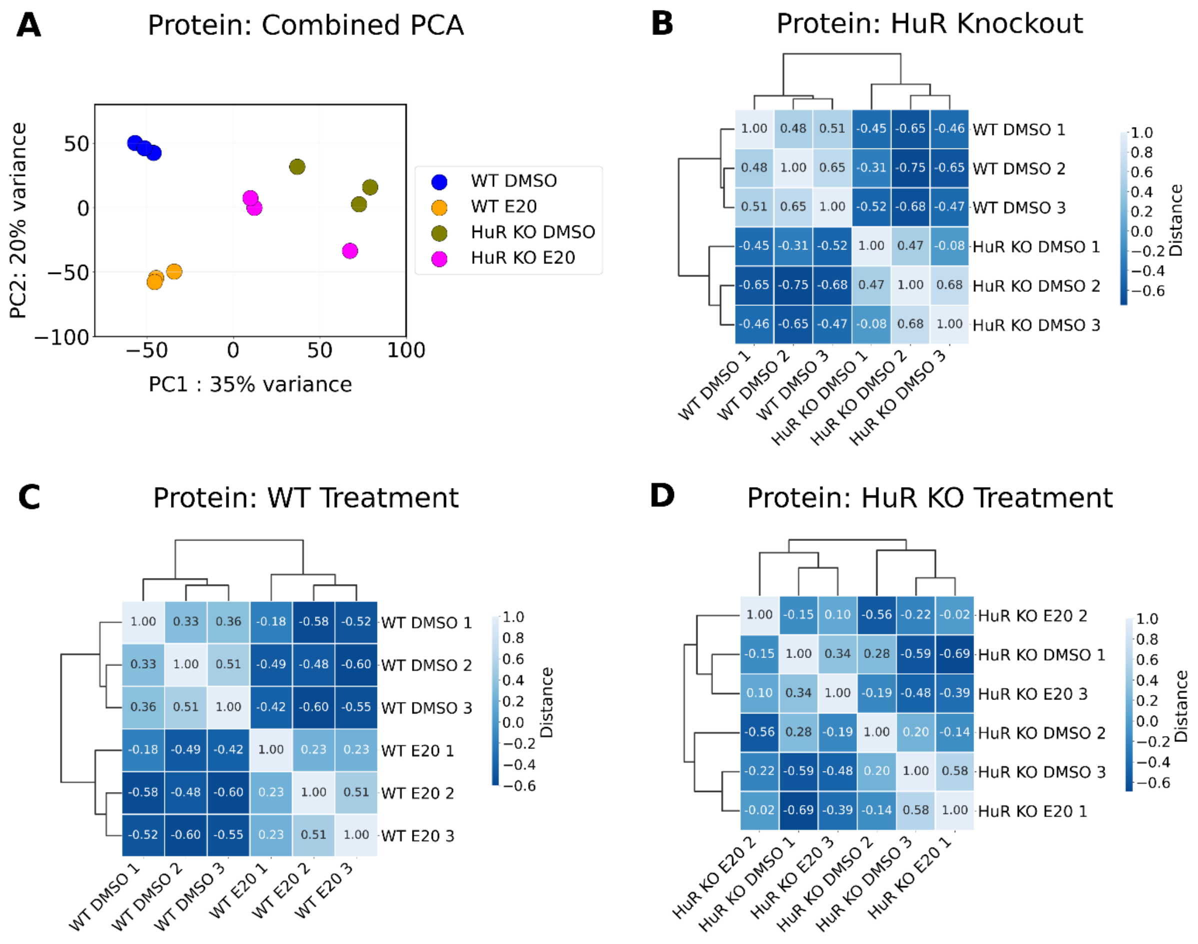

Supplementary Figure 6. Combined PC analysis and sample-to-sample clustering show overlapping results between corresponding samples. (A) Combined PC analysis from mass spectrometry. (B-D) Sample-to-sample distance matrix clustering with calculated pairwise distances (B) HuR Knockout, (C) WT treatment, (D) HuR KO treatment.

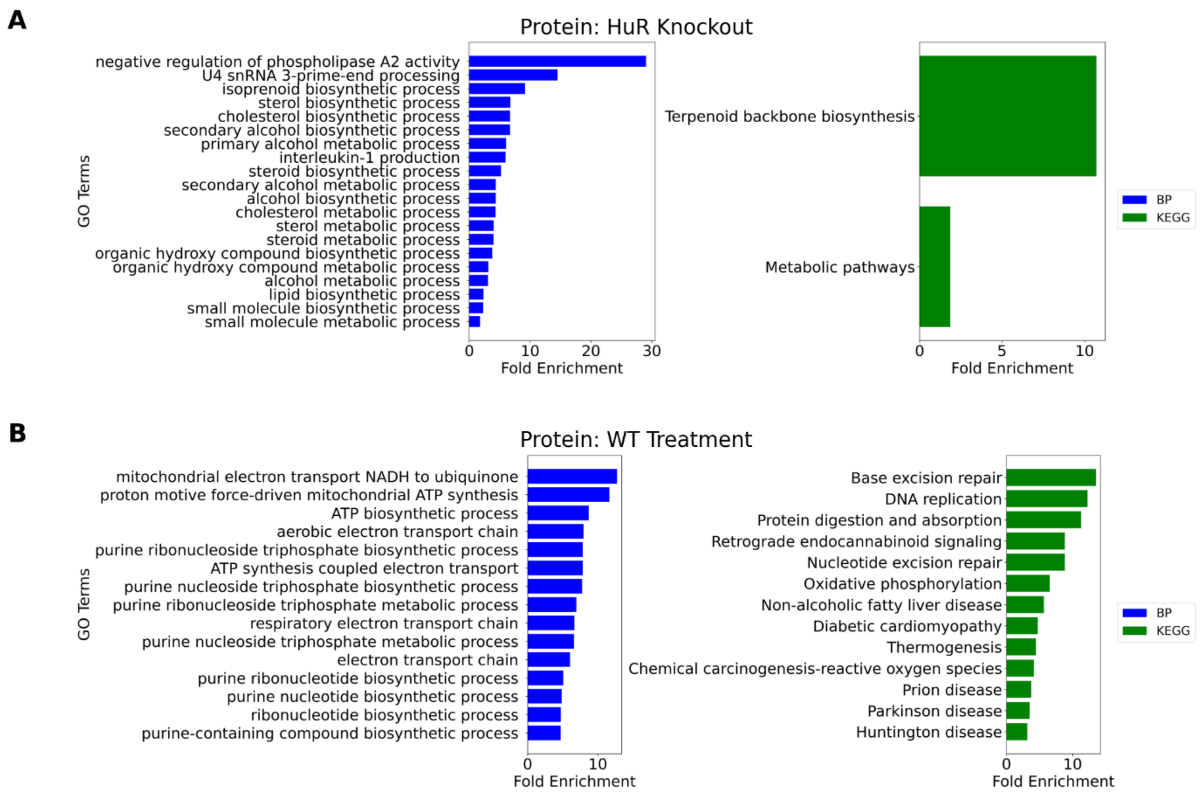

Supplementary Figure 7. GO terms derived from mass spectrometry analysis. Top 20 Enriched GO Terms from Gene Ontology analysis (BP, KEGG) from Mass spectrometry (A). HuR Knockout effect, (B). WT Treatment with Eltrombopag.

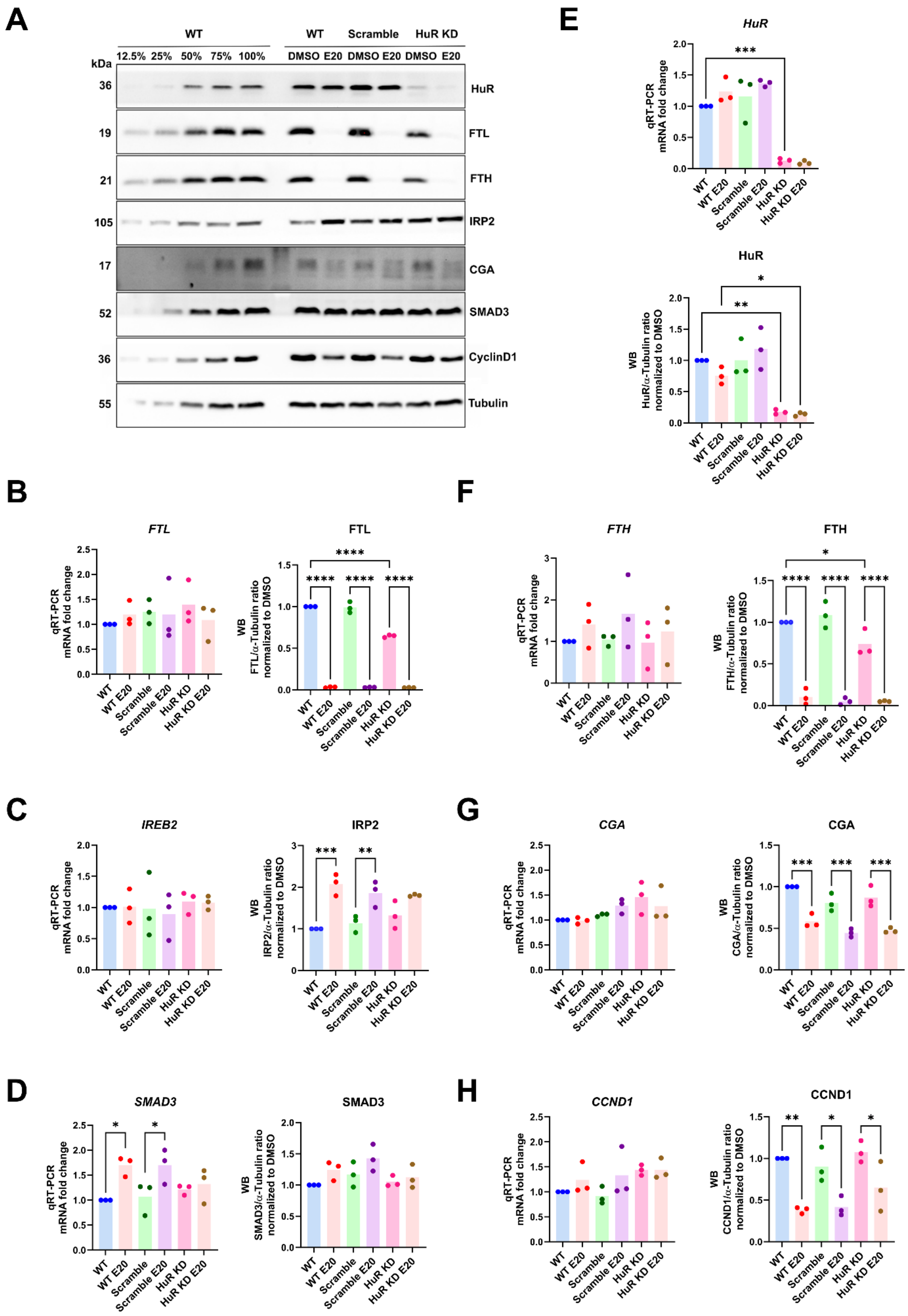

A
WT
12.5% 25% 50% 75% 100%
WT Scramble HuR KD
DMSO E20 DMSO E20 DMSO E20
kDa
36 HuR
19 FTL
21 FTH
105 IRP2
17 CGA
52 SMAD3
36 CyclinD1
55 Tubulin
E
HuR
qRT-PCR mRNA fold change
***
HuR
WB HuR/α-Tubulin ratio normalized to DMSO
**
*
B
FTL
qRT-PCR mRNA fold change
FTL
WB FTL/α-Tubulin ratio normalized to DMSO
****
F
FTH
qRT-PCR mRNA fold change
FTH
WB FTH/α-Tubulin ratio normalized to DMSO
****
*
C
IREB2
qRT-PCR mRNA fold change
IRP2
WB IRP2/α-Tubulin ratio normalized to DMSO
***
**
G
CGA
qRT-PCR mRNA fold change
CGA
WB CGA/α-Tubulin ratio normalized to DMSO
***
D
SMAD3
qRT-PCR mRNA fold change
*
SMAD3
WB SMAD3/α-Tubulin ratio normalized to DMSO
H
CCND1
qRT-PCR mRNA fold change
CCND1
WB CCND1/α-Tubulin ratio normalized to DMSO
**
*
WT
WT E20
Scramble
Scramble E20
HuR KD
HuR KD E20

Supplementary Figure 8. Eltrombopag treatment affects the protein levels after HuR RNAi. (A) Western blot experiment showing protein expression of selected targets (HuR, FTL, FTH, IRP2, CGA, SMAD3, Cyclin D1) after Eltrombopag treatment and transient silencing of HuR with siRNA. The first five lanes (WT 12.5%, WT 25%, WT 50%, WT 75%, WT 100%) represent a gradient of protein levels from untreated WT HeLa cells, serving as a reference to evaluate silencing efficiency. The subsequent lanes depict the following experimental conditions: untreated WT cells, WT cells treated with 20 μM Eltrombopag (WT E20), scramble siRNA transfected cells (Scramble), scramble siRNA transfected cells treated with 20 μM Eltrombopag (Scramble E20), HuR knockdown cells (HuR KD), and HuR knockdown cells treated with 20 μM Eltrombopag (HuR KD E20). α-Tubulin served as an internal control for western blotting. (B - H) Quantification of qRT-PCR and western blot results. Statistical significance was determined using one-way ANOVA with Šidak's multiple comparison adjustment with * denoting $p < 0.05$, ** denoting $p < 0.01$, *** denoting $p < 0.001$ and **** denoting $p < 0.0001$, indicating significant differences between conditions. Individual data values are presented in Additional file 2.

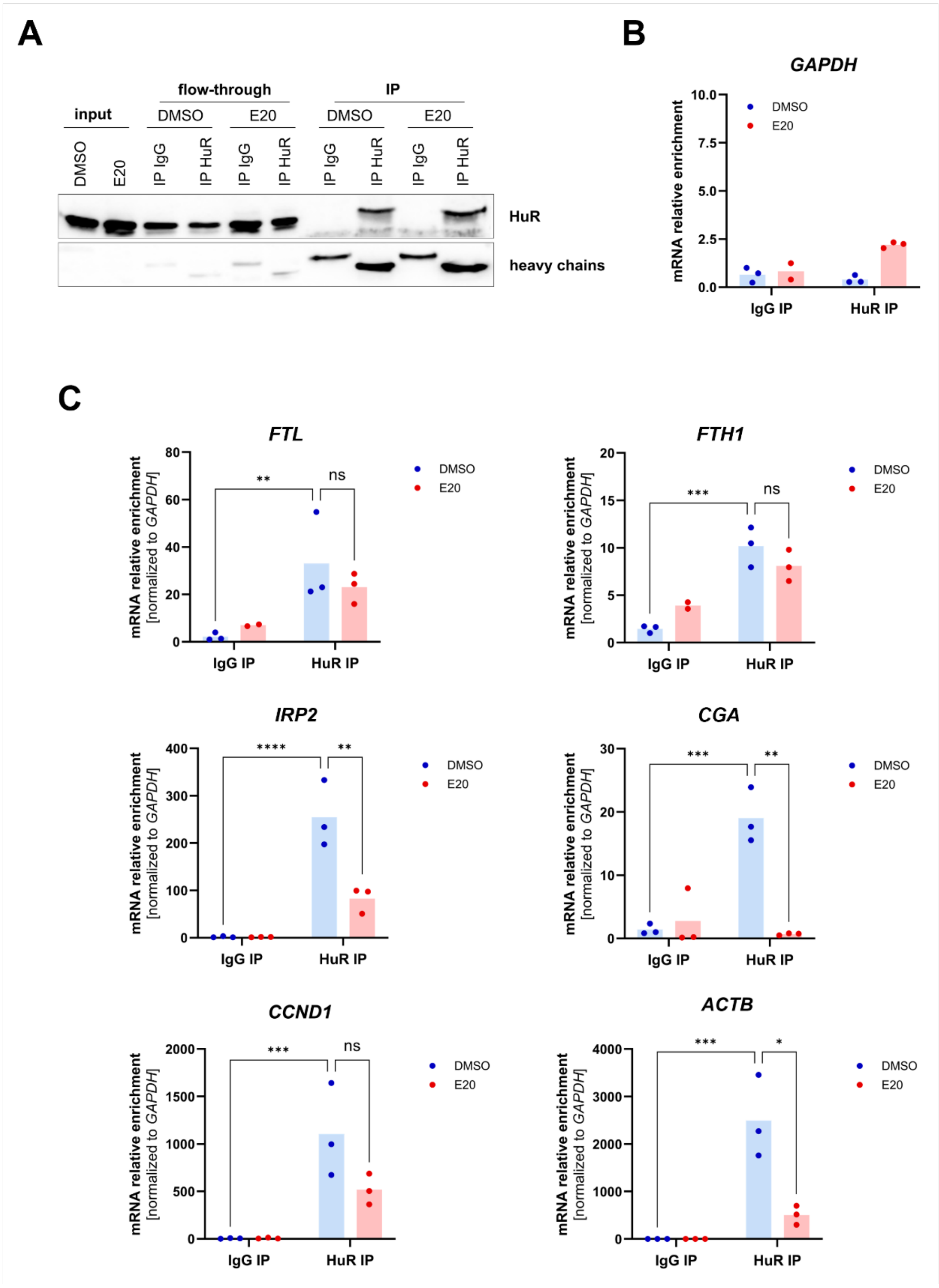

Supplementary Figure 9. Eltrombopag abolishes HuR binding to IREB2 and CGA mRNAs. HeLa WT cells were subjected to DMSO or Eltrombopag treatment for 48 hours then HuR RIP was performed. (A) Western blot analysis of HuR RIP complexes. (B,C) RIP-RT-qPCR (n = 3) of HuR-bound mRNAs: FTL, FTH1, IREB2, CGA, CCND1 and ACTB. The results were normalized to GAPDH mRNA bound in a nonspecific manner. Data were compared using two-way ANOVA with Šídák's multiple comparisons test with * indicating $p < 0.05$, **indicating $p < 0.01$, *** indicating $p < 0.001$ and **** indicating $p < 0.0001$. Individual data values are presented in Additional file 2.

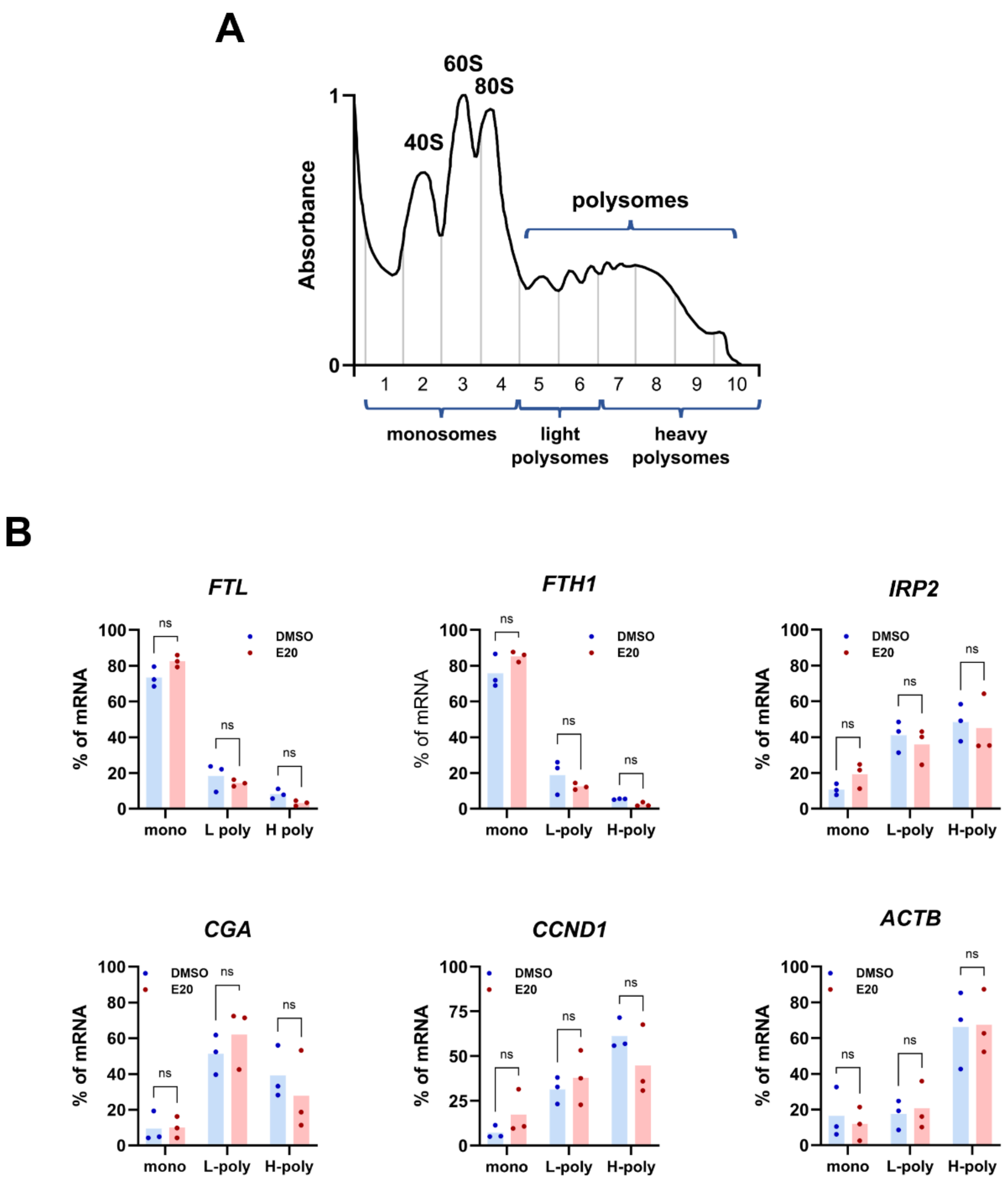

Supplementary Figure 10. 6 hours-Eltrombopag treatment has no effect on translation efficiency. (A) Interpretation of divided fractions from absorbance profile (monosomes (mono), light polysomes (L-poly), heavy polysomes (H-poly)). (B) Polysome profiling of FTL, FTH1, IREB2, CGA, CCND1 and ACTB mRNAs. Graphs represent the mean values with ± SEM from three independent experiments. Statistical significance was determined using two-way ANOVA with Šídák's multiple comparisons test, with "ns" indicating non- significance between treated and nontreated samples. TATAA Universal RNA Spike was utilized as normalization control for qRT-PCR. Individual data values are presented in Additional file 2.

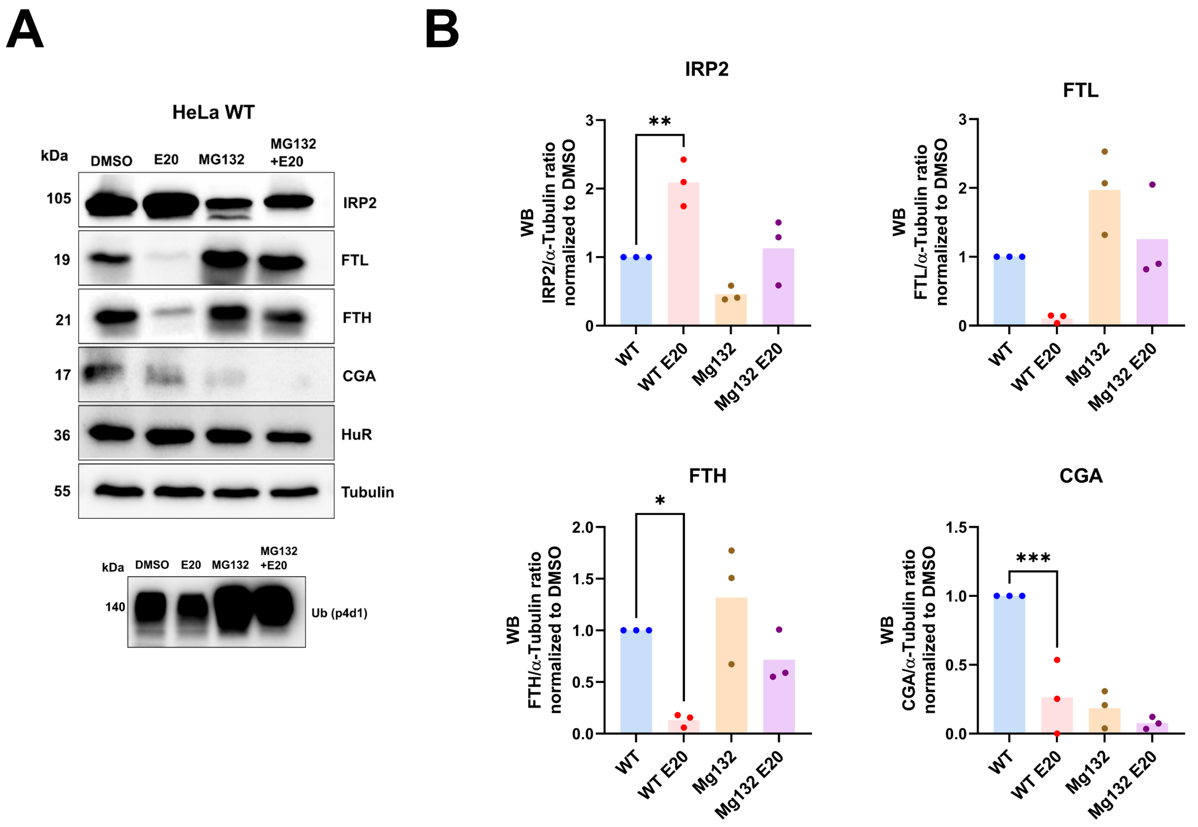

Supplementary Figure 11. Eltrombopag affects proteasome-dependent degradation. (A) Western blot analysis of selected targets (IRP2, FTL, FTH, CGA) following Eltrombopag and (or) MG132 treatment in HeLa WT cells after 24 hours. Ub (p4d1) served as a positive control of the experiment and α-Tubulin served as an internal control for western blotting. (B) Quantification of western blot results. The data were compared using Ordinary one-way Anova with * indicating $p < 0.05$, **indicating $p < 0.01$, *** indicating $p < 0.001$. The data are represented as three biological replicates. Individual data values are presented in Additional file 2.

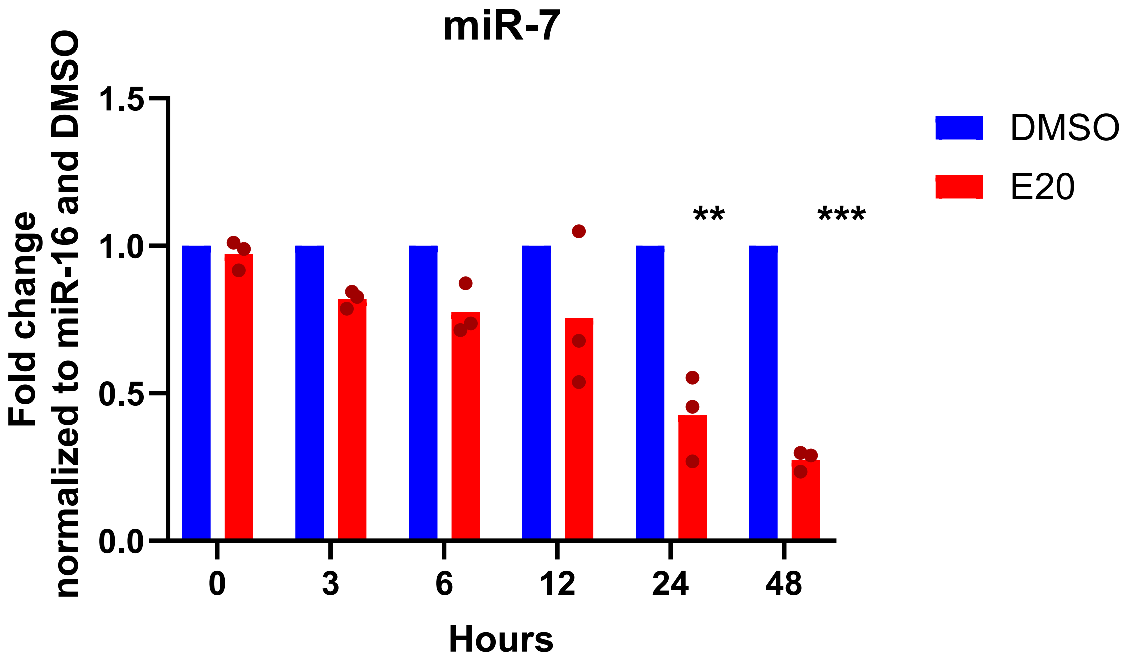

Supplementary Figure 12. Eltrombopag decreases the expression of mature miR-7 in a time-dependent manner. HeLa cells were treated with Eltrombopag for 3, 6, 12, 24 and 48 hours, total RNA was extracted, and qRT-PCR was performed. miR-16 was used as a housekeeping miR control. The data are represented as three biological replicates. Ordinary one-way anova with Dunnett's multiple comparison test was used with * indicating $p < 0.05$, **indicating $p < 0.01$, *** indicating $p < 0.001$. Individual data values are presented in Additional file 2.

Supplementary Table 1. HuR dependent targets

Genes	HuR KO-DMSO vs WT-DMSO				WT-Eltrombopag vs WT-DMSO				HuR KO-Eltrombopag vs HuR KO-DMSO			
	RNAseq		MS		RNAseq		MS		RNAseq		MS	
	log2 FC	padj	log2 FC	padj	log2 FC	padj	log2 FC	padj	log2 FC	padj	log2 FC	padj
**FTL**	0.758	1.01E-13	-3.283	0.363	-0.247	0.484	-18.081	3.00E-05	-0.918	2.58E-05	-3.171	0.811
**FTH1**	0.654	7.92E-05	-0.522	0.181	0.629	0.054	-1.442	0.023	-0.454	0.021	-0.014	0.991
**IREB2**	-0.281	0.387	1.538	0.025	0.472	0.225	1.480	0.003	0.882	0.138	0.517	0.811
**CCND1**	-0.342	0.099	-0.331	0.200	0.396	0.087	-2.187	0.007	0.249	0.281	-0.543	0.811
**SMAD3**	1.029	2.20E-22	10.587	1.42E-06	0.745	0.095	0.239	0.167	-0.091	0.752	0.082	0.901
**CGA**	-4.387	4.25E-37	-19.225	8.88E-07	-2.779	5.36E-04	-2.556	0.004	-0.200	0.949	0.054	0.865

Supplementary Table 2. List of primers sequences.

Gene	Primer variant	Sequence
**FTL**	Forward	5′-CTTGCCAACCAACCATGAGC-3′
	Reverse	5′-CGAAATAGAAGCCCAGAGAGAGG-3′
**CCND1**	Forward	5′-CGTGGCCTCTAAGATGAAGG-3′
	Reverse	5′-CTGGCATTTTGGAGAGGAAG-3′
**SMAD3**	Forward	5′-CATCGAGCCCCAGAGCAATA-3′
	Reverse	5′-GTGGTTCATCTGGTGGTCACT-3′
**FTH1**	Forward	5′-AAGCTGCAGAACCAACGAGG-3′
	Reverse	5′-AGTCACACAAATGGGGGTCATT-3′
**CGA**	Forward	5′-TCCCACTCCACTAAGGTCCAA-3′
	Reverse	5′-CCCCATTACTGTGACCCTGTT-3′
**IREB2**	Forward	5′-CGCCTTTGAGTACCTTATTGAAACA-3′
	Reverse	5′-CGTACAGCAGCTTCCAACAAGA-3′

**GAPDH**	Forward	5′-AATCCCATCACCATCTTCCA-3′
	Reverse	5′-TGGACTCCACGACGTACTCA-3′
**ACTB**	Forward	5′-GCATGGGTCAGAAGGATTCC-3′
	Reverse	5′-CCACACGCAGCTCATTGTAG-3′
**miR-7-5p**	Forward	5′-TGGAAGACTAGTGATTTTGTTGTT-3’
**miR-16**	Forward	5’-TAGCAGCACGTAAATATTGGCG-3’

Supplementary Table 3. List with primary antibodies used for the experiments.

Reagent type	Designation	Source	Identifiers	Dilution
Antibody	Anti-human/mouse Ferritin Heavy Chain (FTH1) (Rabbit polyclonal)	Cell Signaling Technology	Cat. # 3998	1:1000
Antibody	Anti-human/mouse Ferritin Light Chain (FTL) (Rabbit polyclonal)	Abcam	Cat. # ab6909	1:1000
Antibody	Anti-human SMAD3 (C67H9) (Rabbit monoclonal)	Cell Signaling Technology	Cat. # 9523	1:1000
Antibody	Anti-human IRP2 (D6E6W) (Rabbit monoclonal)	Cell Signaling Technology	Cat. # 37135	1:1000
Antibody	Anti-human Cyclin D1 (Rabbit polyclonal)	Cell Signaling Technology	Cat. # 2922	1:1000
Antibody	Anti-human CGA (Rabbit polyclonal)	Thermofisher	Cat. # PA5-88517	1:000
Antibody	Anti-human HuR (ELAV1) (Rabbit polyclonal)	EMD Millipore	Cat. # 07-1735	1:1000
Antibody	Anti-human Alpha Tubulin (Rabbit polyclonal)	Proteintech	Cat. # 11224-1-AP	1:4000
Antibody	Anti-HuR (IgG1 κ mouse monoclonal)	Santa Cruz Biotechnology	Cat. # sc-5261	1:1000
