# Supplementary material for: Effects of genetic ablation and pharmacological inhibition of HuR on gene expression, iron metabolism, and hormone levels
Source: BMC Biol. 2025 Jan 23;23:24. doi: 10.1186/s12915-025-02131-z (PMC11756078; doi:10.1186/s12915-025-02131-z)
Supplement: Supplementary file 3 — Additional file 3: Uncropped images of the original western blots. [file 12915_2025_2131_MOESM3_ESM.pdf]

Uncropped images of the original western blots. Blots used in the figures are highlighted.

Figure 5A

FTL

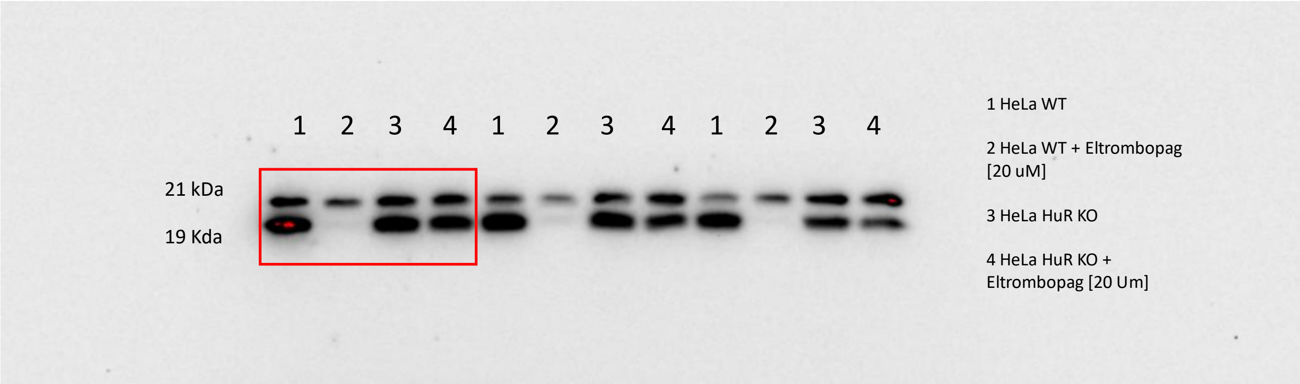

FTH

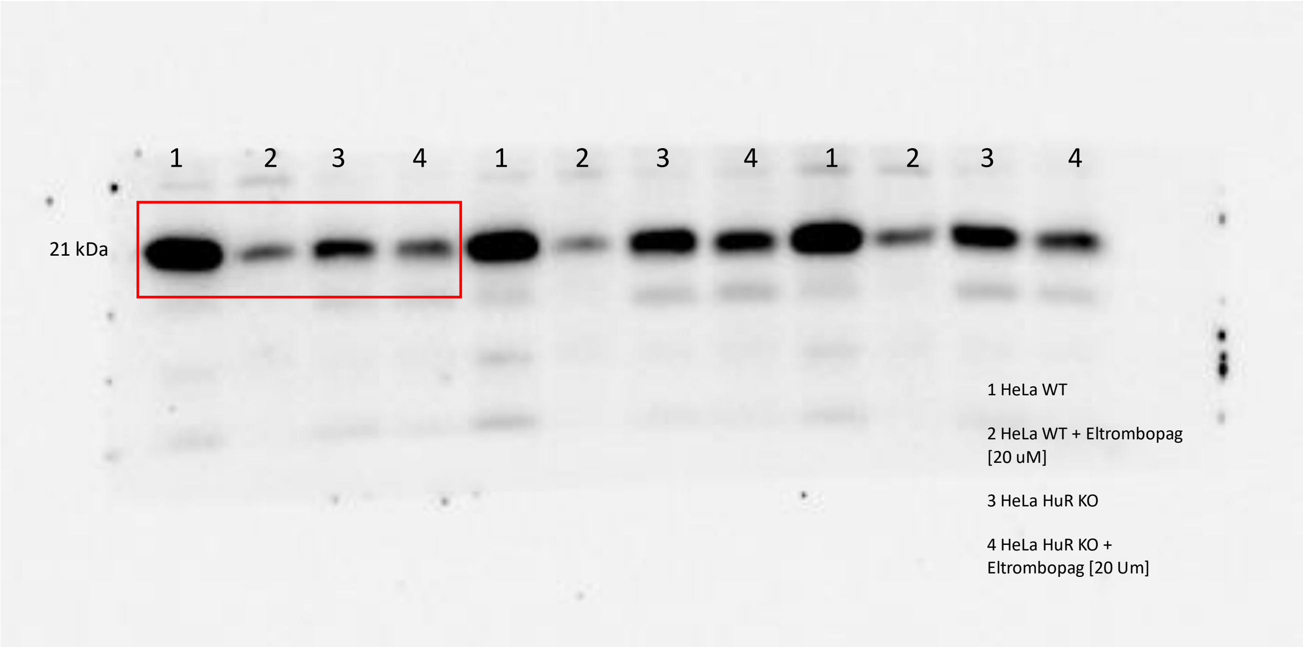

IRP2

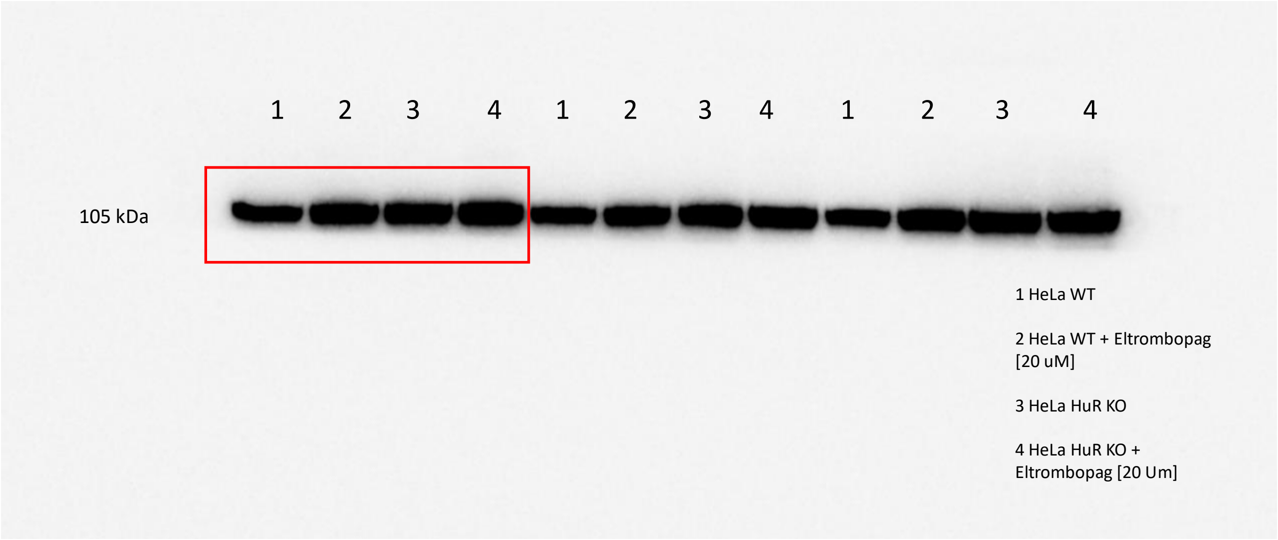
1 2 3 4 1 2 3 4 1 2 3 4
105 kDa
1 HeLa WT
2 HeLa WT + Eltrombopag [20 uM]
3 HeLa HuR KO
4 HeLa HuR KO + Eltrombopag [20 Um]

CGA

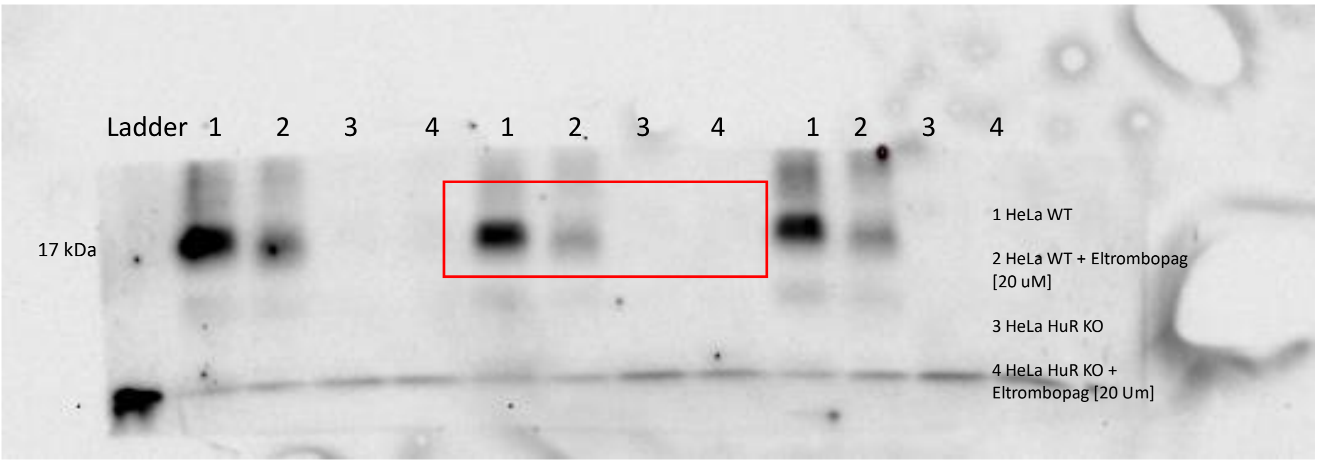
Ladder 1 2 3 4 1 2 3 4 1 2 3 4
17 kDa
1 HeLa WT
2 HeLa WT + Eltrombopag [20 uM]
3 HeLa HuR KO
4 HeLa HuR KO + Eltrombopag [20 Um]

SMAD3

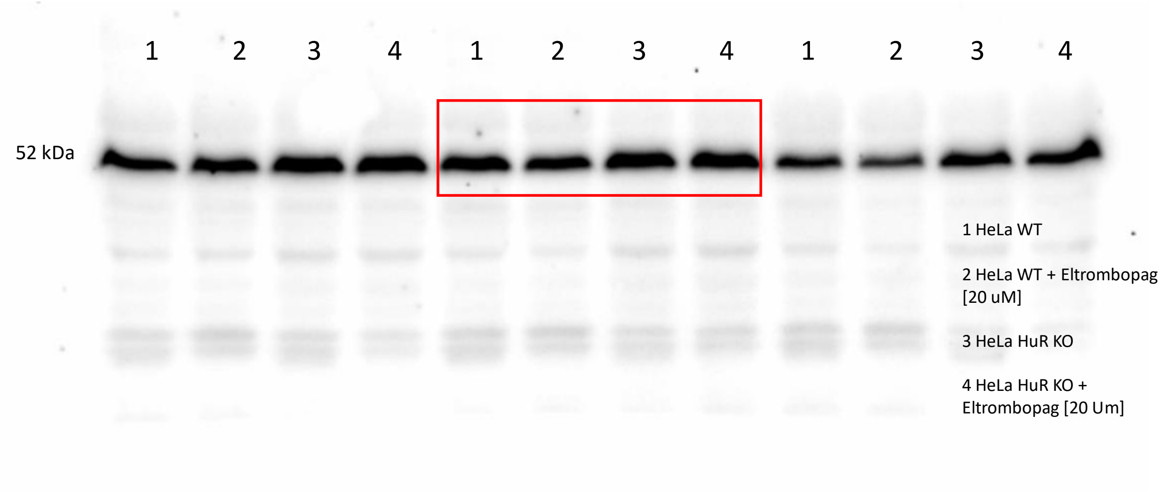
1 2 3 4 1 2 3 4 1 2 3 4
52 kDa
1 HeLa WT
2 HeLa WT + Eltrombopag [20 uM]
3 HeLa HuR KO
4 HeLa HuR KO + Eltrombopag [20 Um]

HuR

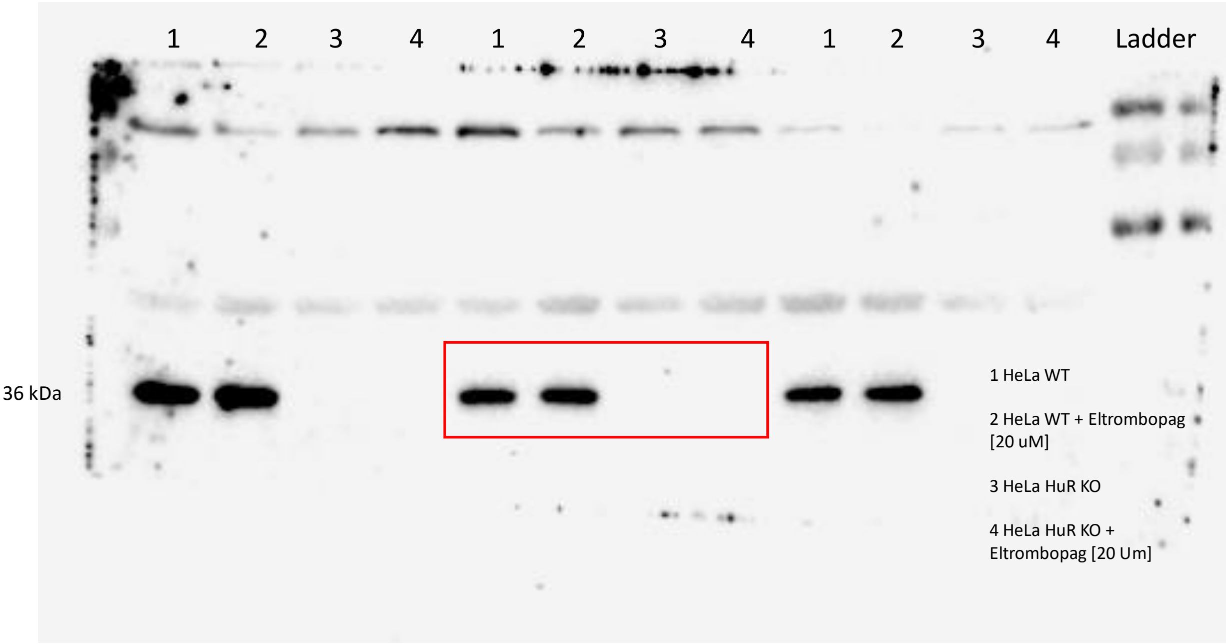
1 2 3 4 1 2 3 4 1 2 3 4 Ladder
36 kDa
1 HeLa WT
2 HeLa WT + Eltrombopag [20 uM]
3 HeLa HuR KO
4 HeLa HuR KO + Eltrombopag [20 Um]

CCND1

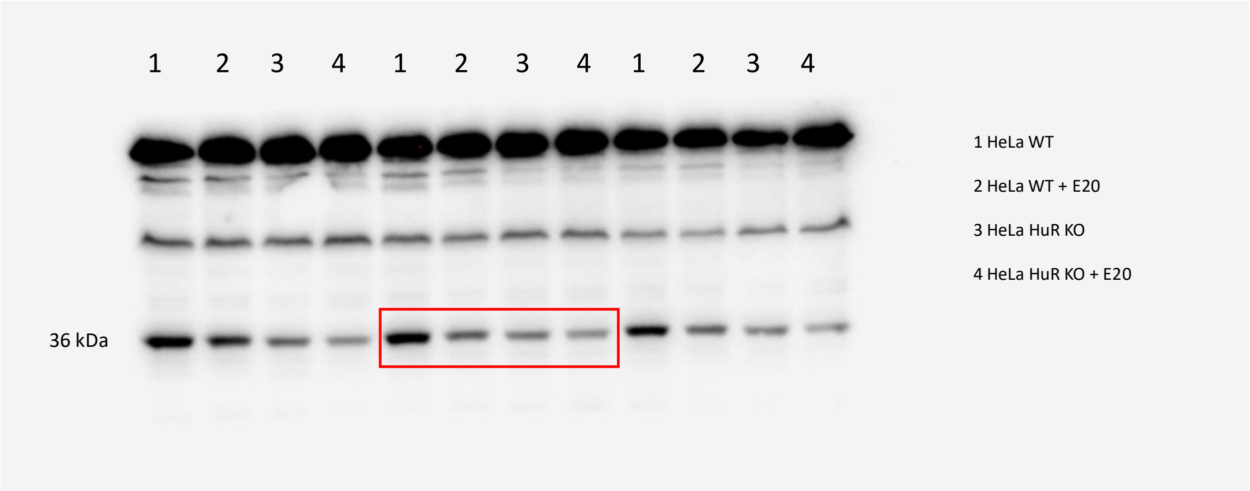

Tubulin

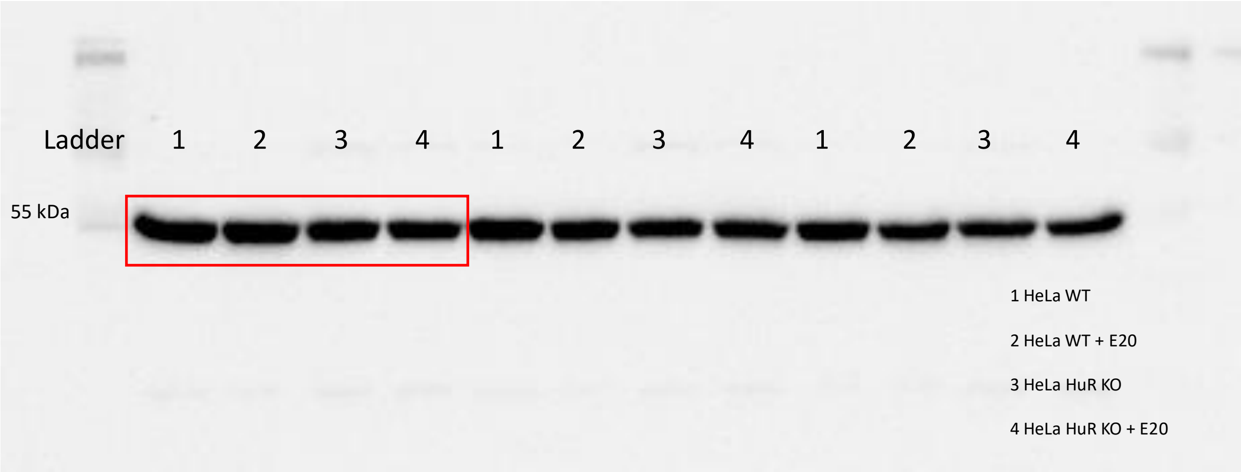

Figure 6A

IRP2

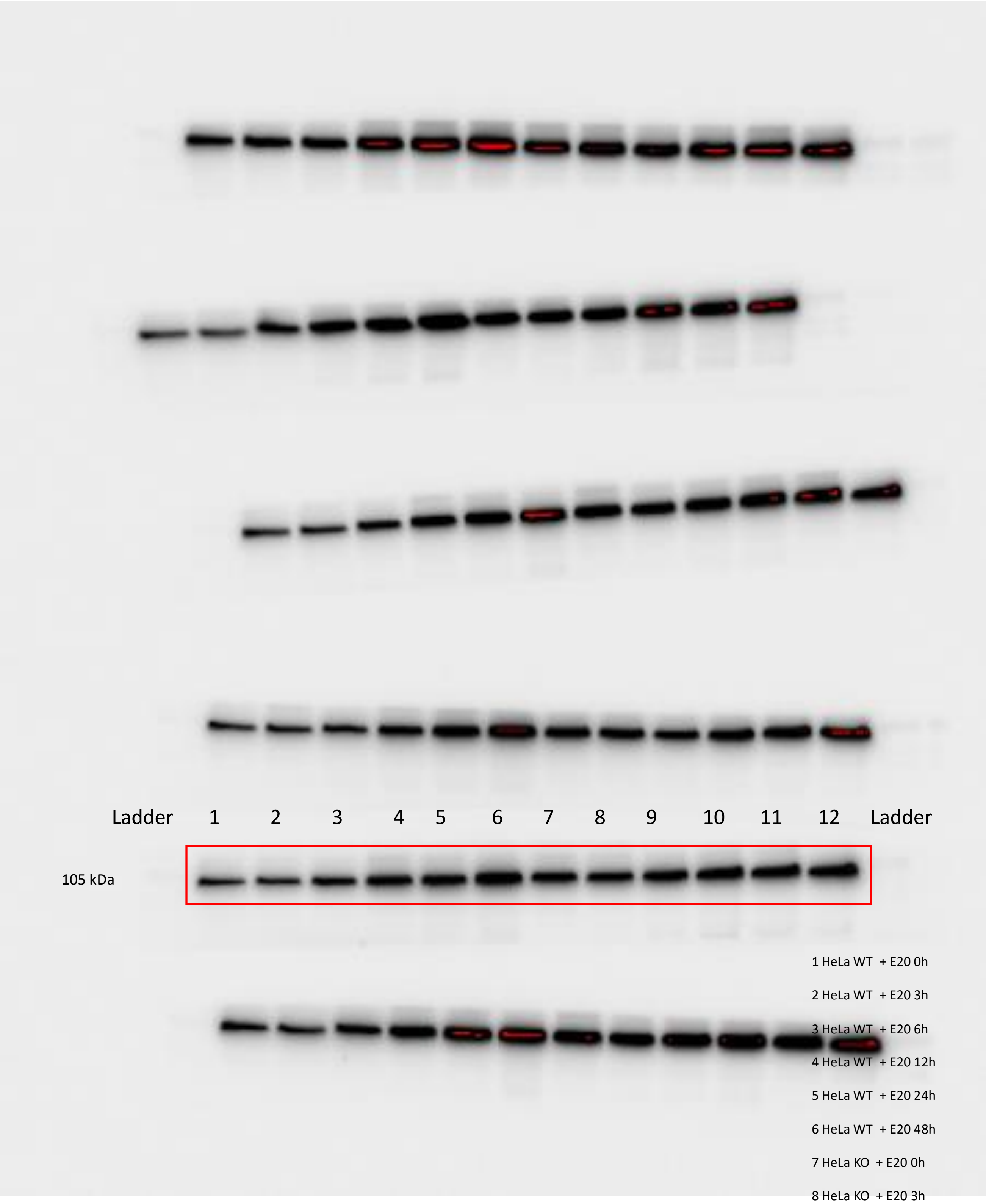

1 HeLa WT + E20 0h

2 HeLa WT + E20 3h

3 HeLa WT + E20 6h

4 HeLa WT + E20 12h

5 HeLa WT + E20 24h

6 HeLa WT + E20 48h

7 HeLa KO + E20 0h

8 HeLa KO + E20 3h

9 HeLa KO + E20 6h

10 HeLa KO + E20 12h

11 HeLa KO + E20 24h

12 HeLa KO + E20 48h

FTL

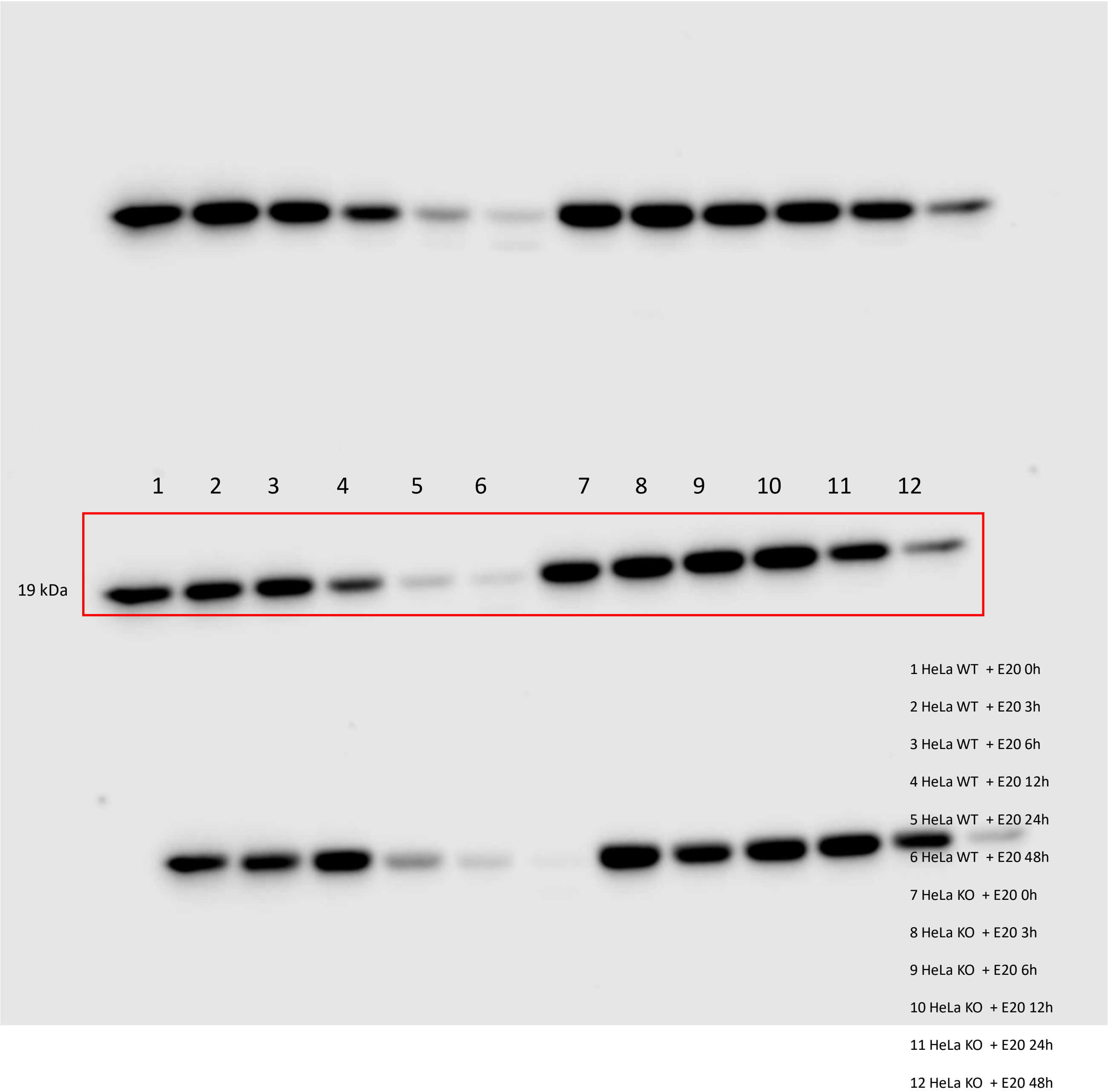
1 2 3 4 5 6 7 8 9 10 11 12
19 kDa
1 HeLa WT + E20 0h
2 HeLa WT + E20 3h
3 HeLa WT + E20 6h
4 HeLa WT + E20 12h
5 HeLa WT + E20 24h
6 HeLa WT + E20 48h
7 HeLa KO + E20 0h
8 HeLa KO + E20 3h
9 HeLa KO + E20 6h
10 HeLa KO + E20 12h
11 HeLa KO + E20 24h
12 HeLa KO + E20 48h

FTH

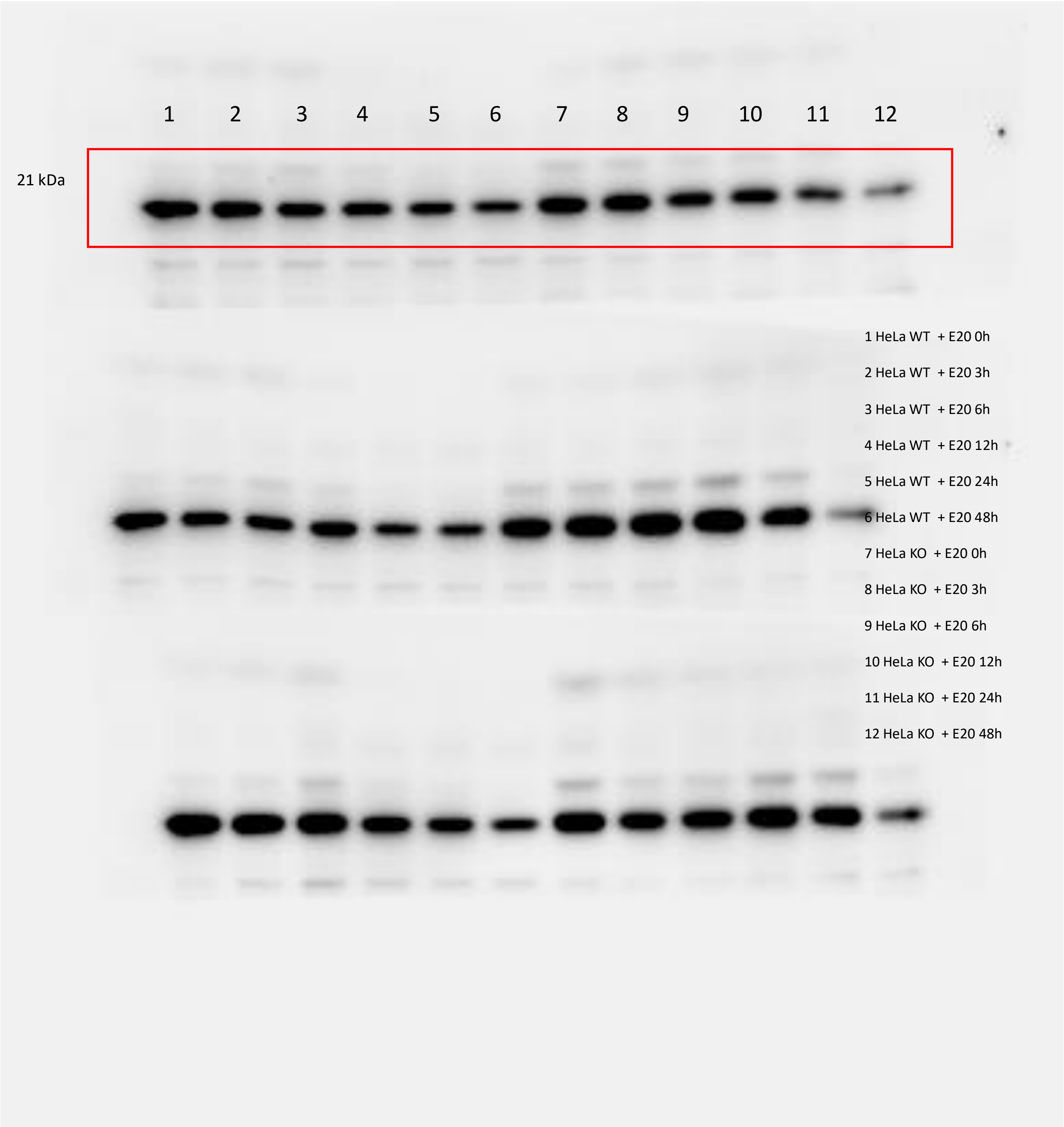

1 2 3 4 5 6 7 8 9 10 11 12
21 kDa
1 HeLa WT + E20 0h
2 HeLa WT + E20 3h
3 HeLa WT + E20 6h
4 HeLa WT + E20 12h
5 HeLa WT + E20 24h
6 HeLa WT + E20 48h
7 HeLa KO + E20 0h
8 HeLa KO + E20 3h
9 HeLa KO + E20 6h
10 HeLa KO + E20 12h
11 HeLa KO + E20 24h
12 HeLa KO + E20 48h

HuR

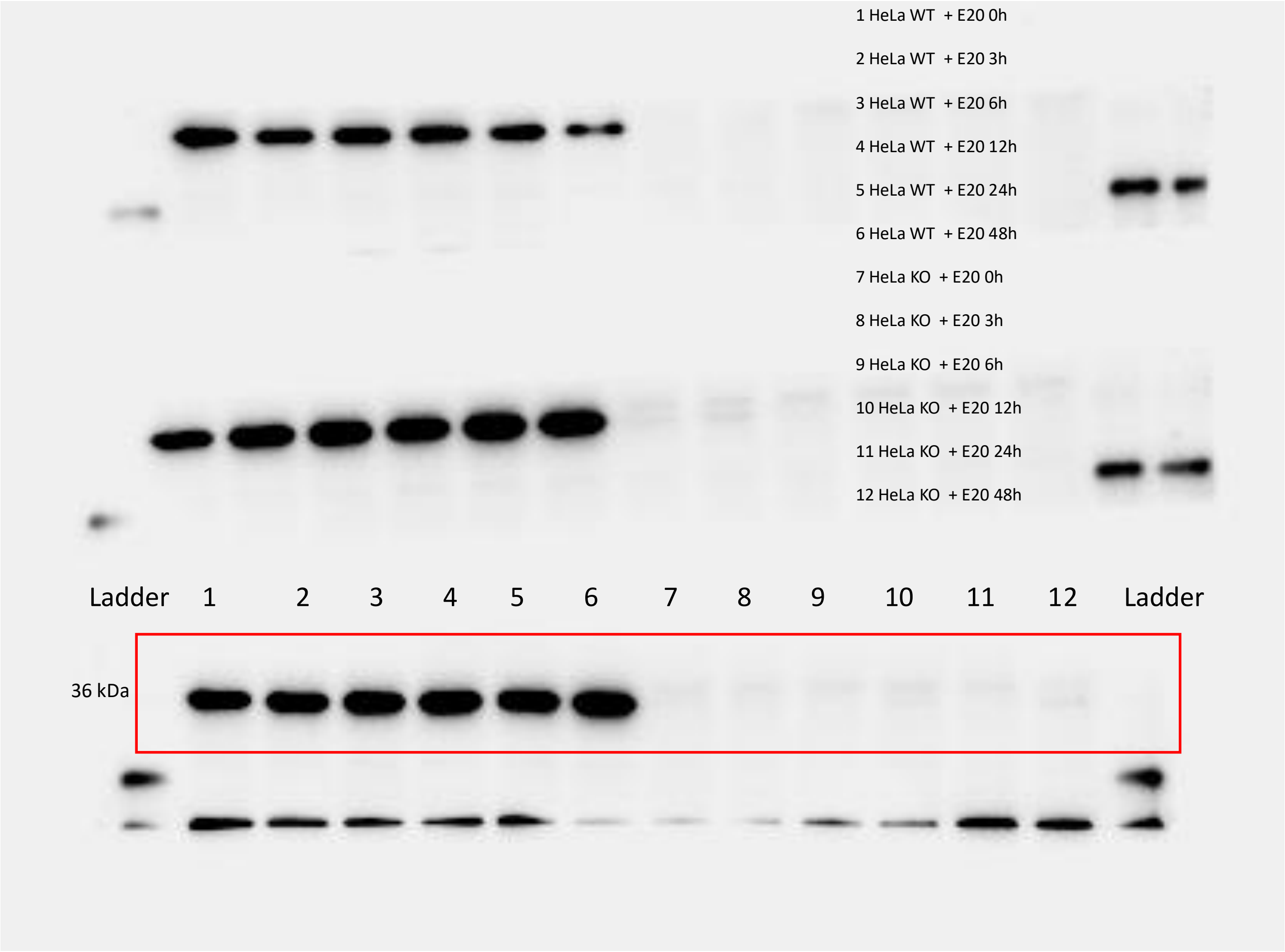
1 HeLa WT + E20 0h
2 HeLa WT + E20 3h
3 HeLa WT + E20 6h
4 HeLa WT + E20 12h
5 HeLa WT + E20 24h
6 HeLa WT + E20 48h
7 HeLa KO + E20 0h
8 HeLa KO + E20 3h
9 HeLa KO + E20 6h
10 HeLa KO + E20 12h
11 HeLa KO + E20 24h
12 HeLa KO + E20 48h
Ladder 1 2 3 4 5 6 7 8 9 10 11 12 Ladder
36 kDa

CGA

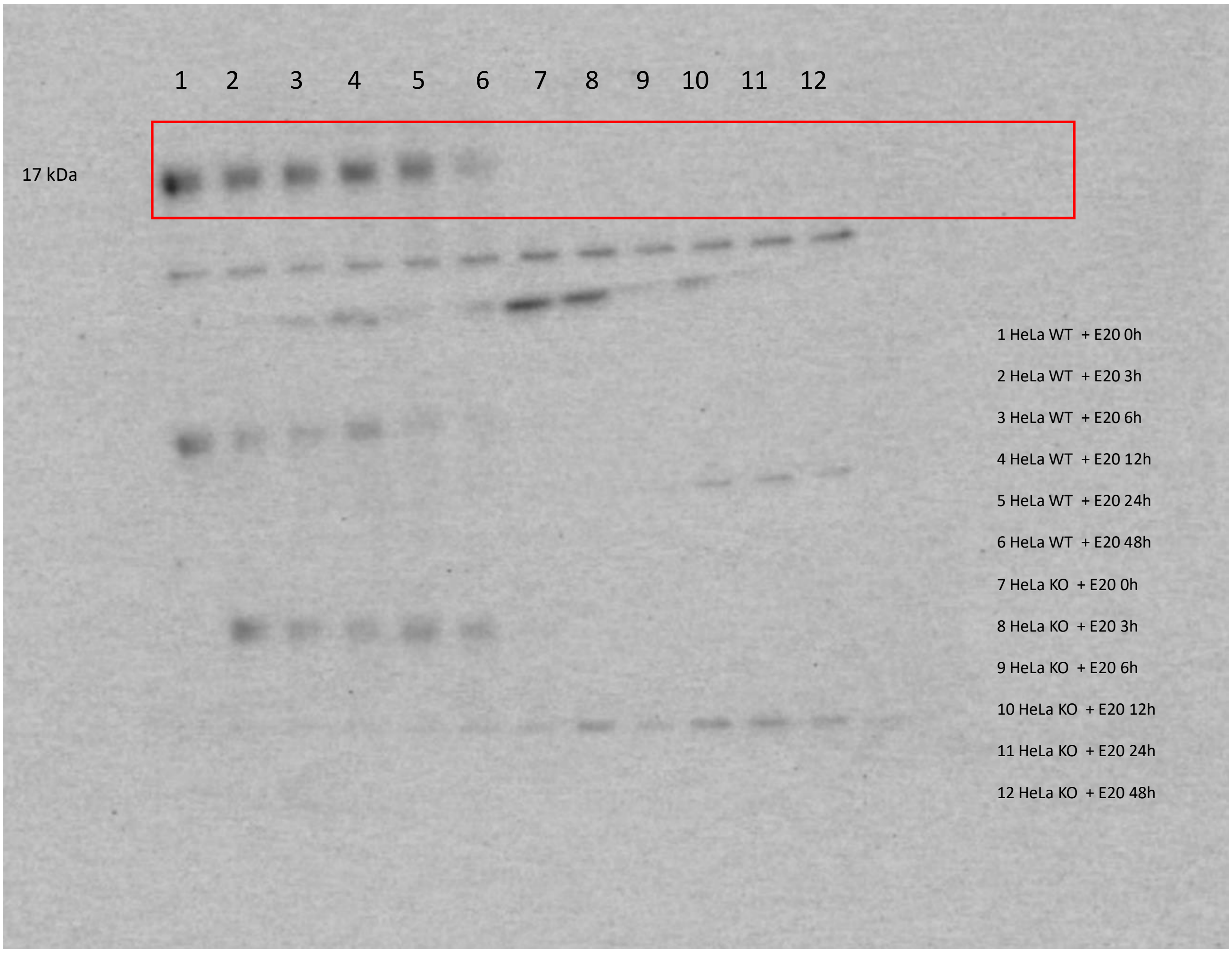

1 2 3 4 5 6 7 8 9 10 11 12
17 kDa
1 HeLa WT + E20 0h
2 HeLa WT + E20 3h
3 HeLa WT + E20 6h
4 HeLa WT + E20 12h
5 HeLa WT + E20 24h
6 HeLa WT + E20 48h
7 HeLa KO + E20 0h
8 HeLa KO + E20 3h
9 HeLa KO + E20 6h
10 HeLa KO + E20 12h
11 HeLa KO + E20 24h
12 HeLa KO + E20 48h

Tubulin

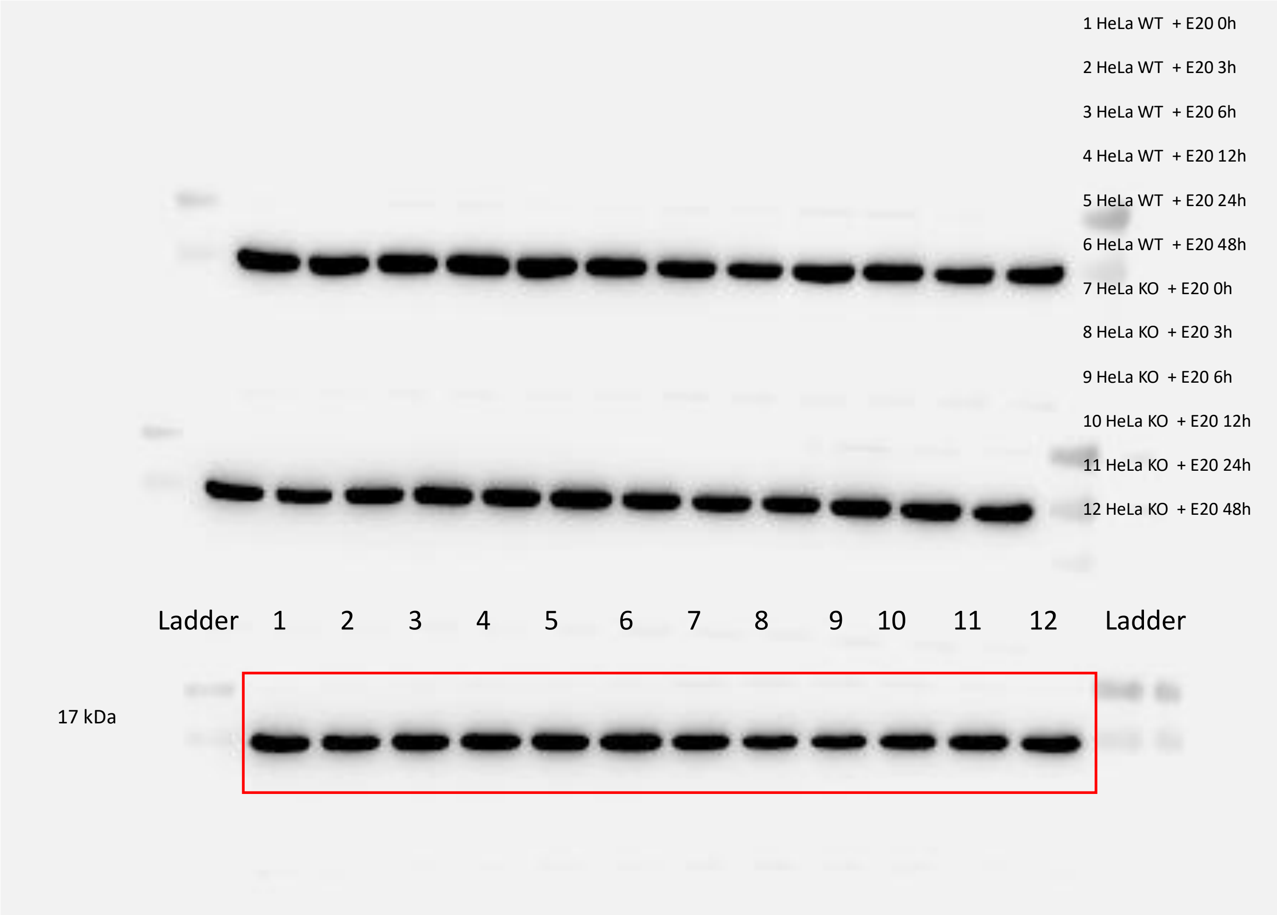
1 HeLa WT + E20 0h
2 HeLa WT + E20 3h
3 HeLa WT + E20 6h
4 HeLa WT + E20 12h
5 HeLa WT + E20 24h
6 HeLa WT + E20 48h
7 HeLa KO + E20 0h
8 HeLa KO + E20 3h
9 HeLa KO + E20 6h
10 HeLa KO + E20 12h
11 HeLa KO + E20 24h
12 HeLa KO + E20 48h
Ladder 1 2 3 4 5 6 7 8 9 10 11 12 Ladder
17 kDa

Figure 7B

HuR 6h Mock (upper panel) 6h E20 (lower panel)

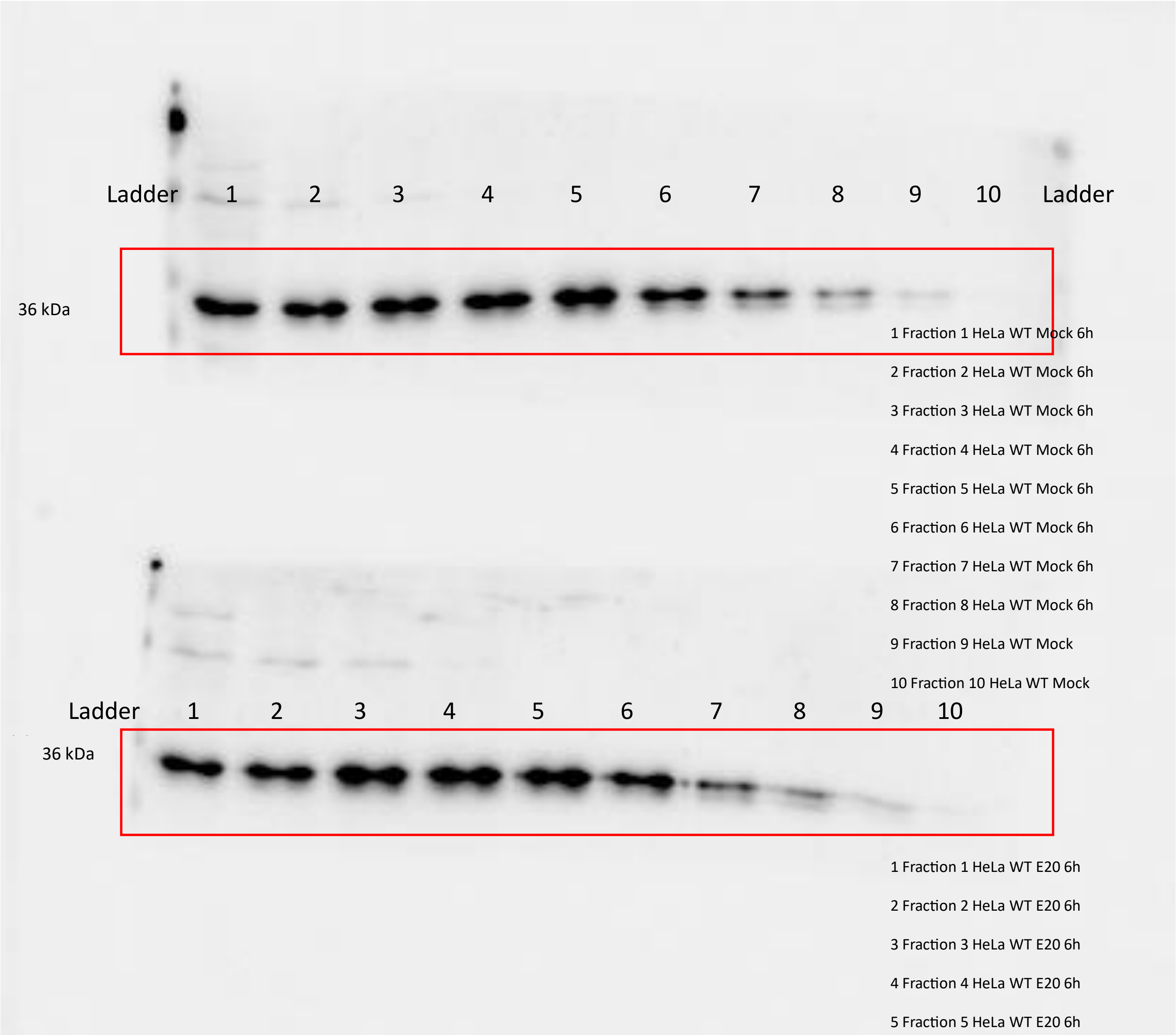

6 Fraction 6 HeLa WT E20 6h

7 Fraction 7 HeLa WT E20 6h

8 Fraction 8 HeLa WT E20 6h

9 Fraction 9 HeLa WT E20 6h

10 Fraction 10 HeLa WT E20 6h

HuR 48h Mock (upper panel) 48h E20 (lower panel)

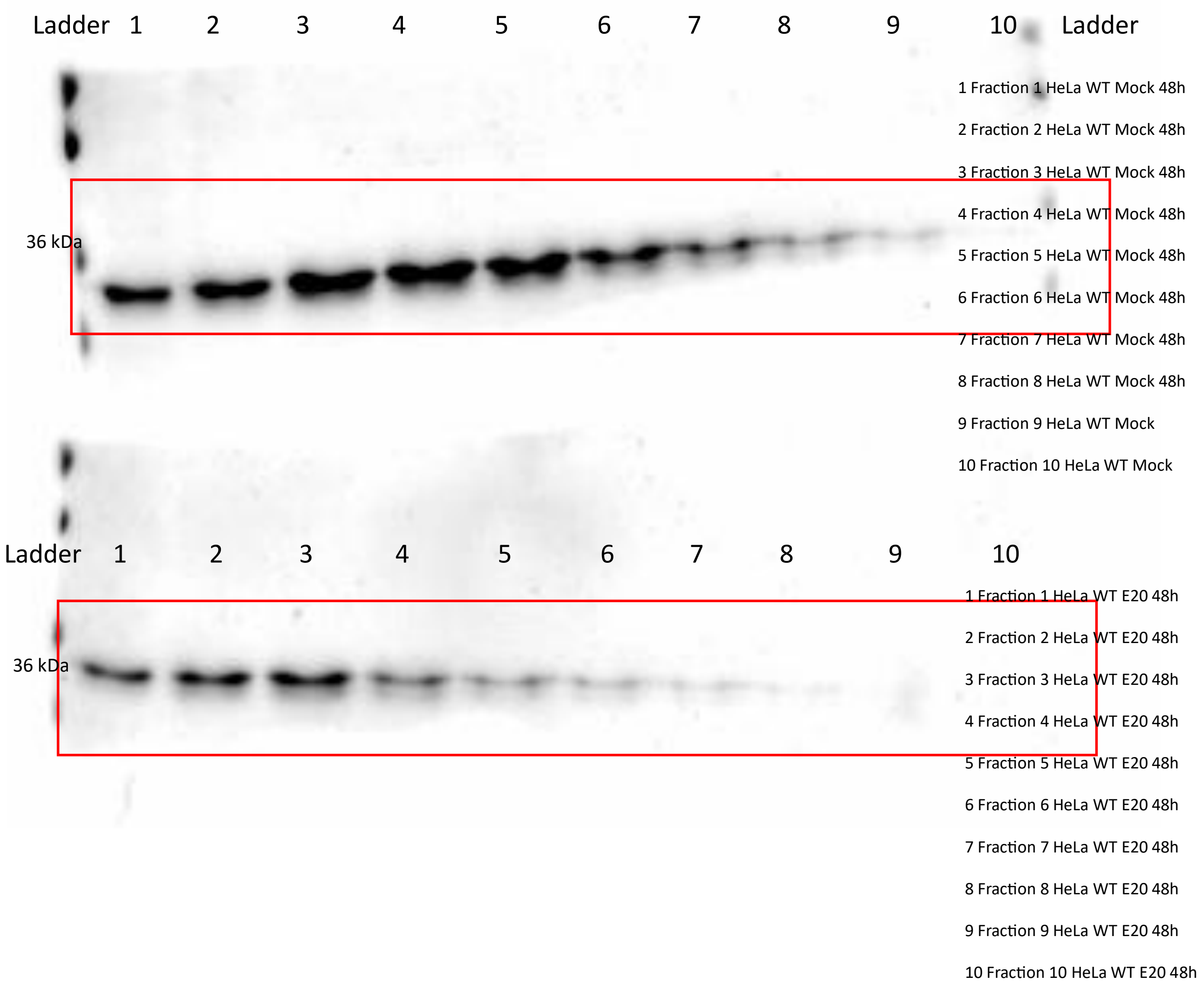

Additional file 1: Fig. 8A

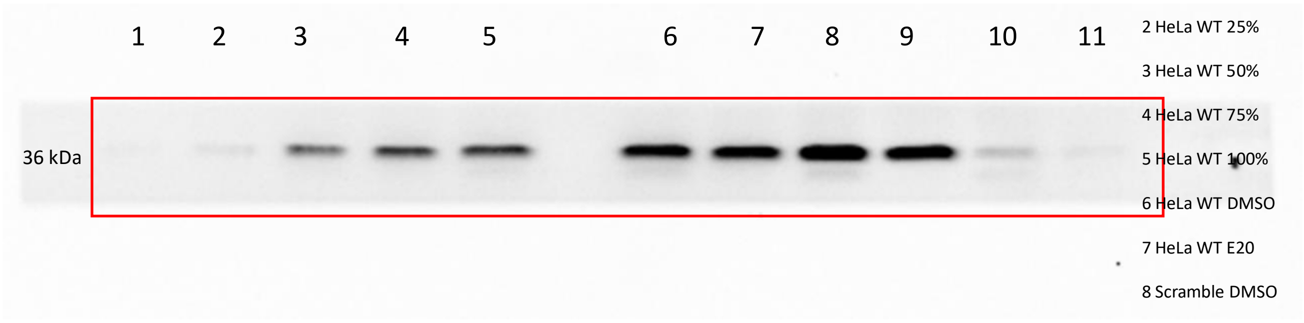

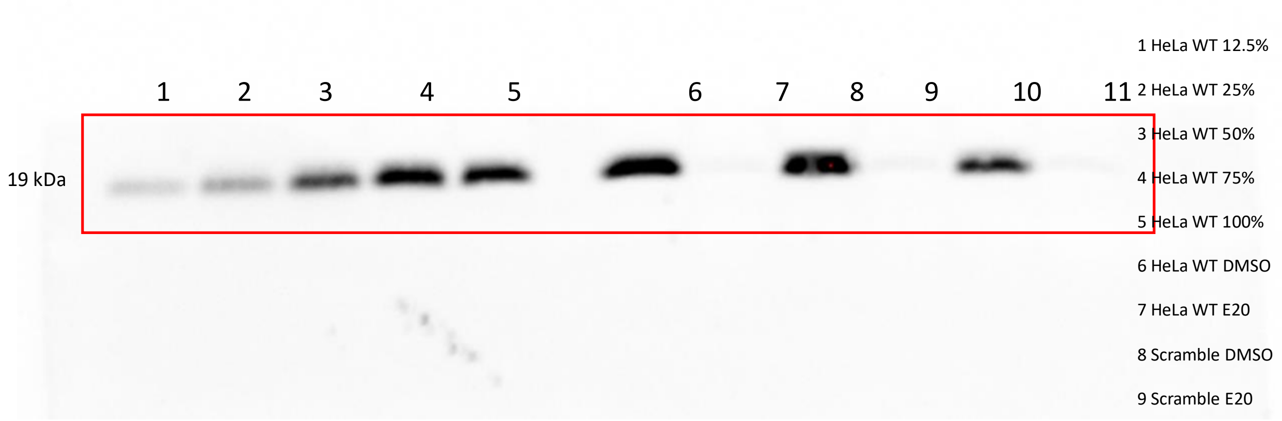

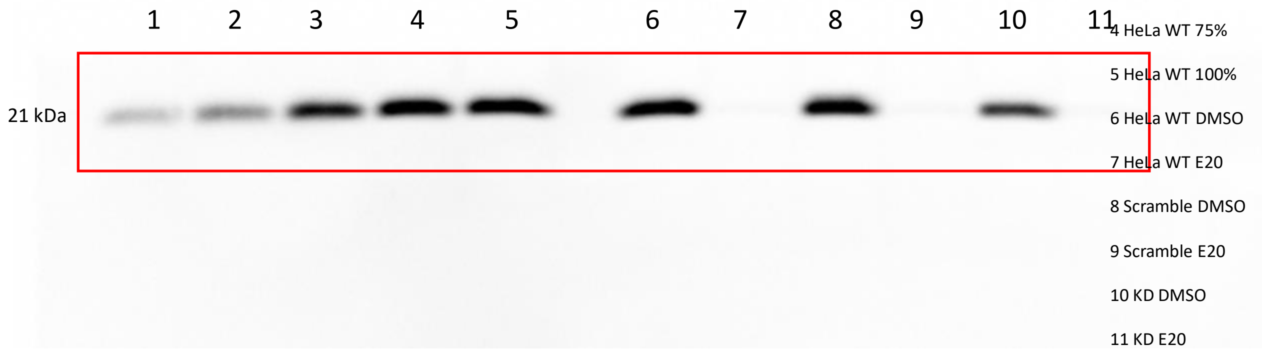

IRP2

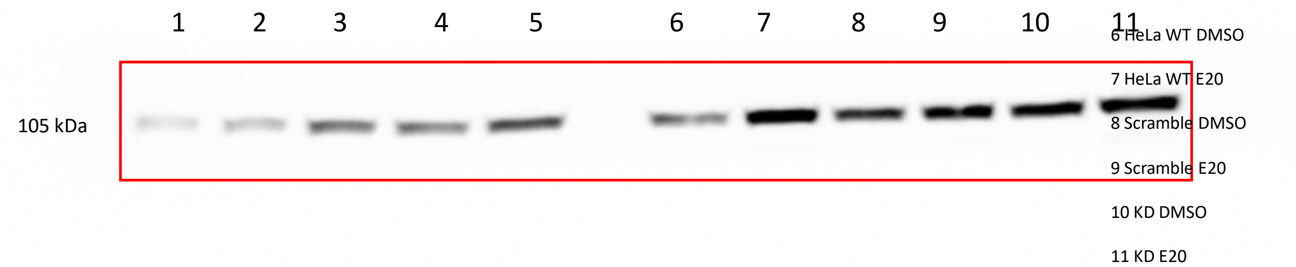

1 HeLa WT 12.5%
2 HeLa WT 25%
3 HeLa WT 50%
4 HeLa WT 75%
5 HeLa WT 100%
6 HeLa WT DMSO
7 HeLa WT E20
8 Scramble DMSO
9 Scramble E20
10 KD DMSO
11 KD E20

CGA

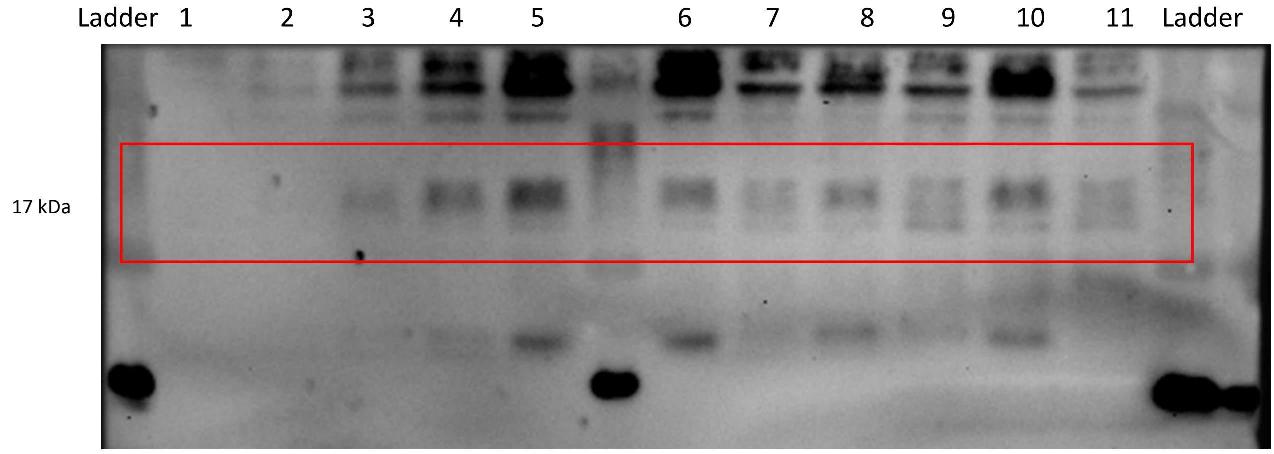

1 HeLa WT 12.5% 2 HeLa WT 25% 3 HeLa WT 50% 4 HeLa WT 75% 5 HeLa WT 100% 6 HeLa WT DMSO 7 HeLa WT E20 8 Scramble DMSO 9 Scramble E20 10 KD DMSO 11 KD E20

SMAD3

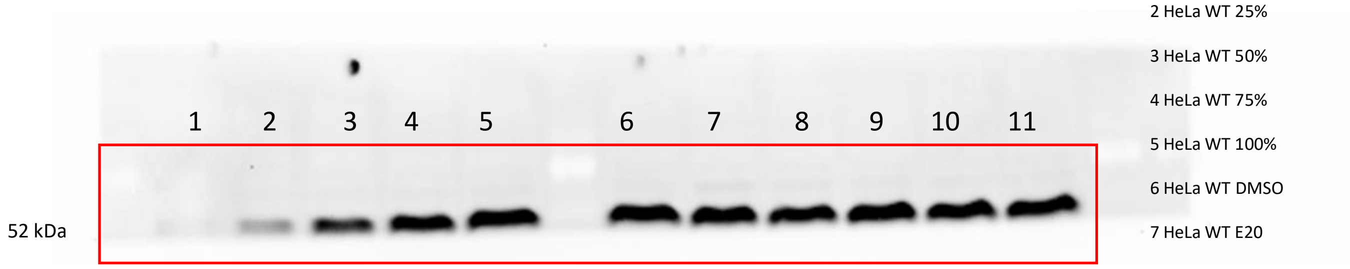

1 HeLa WT 12.5%
2 HeLa WT 25%
3 HeLa WT 50%
4 HeLa WT 75%
5 HeLa WT 100%
6 HeLa WT DMSO
7 HeLa WT E20
8 Scramble DMSO
9 Scramble E20
10 KD DMSO
11 KD E20

Cyclin D1

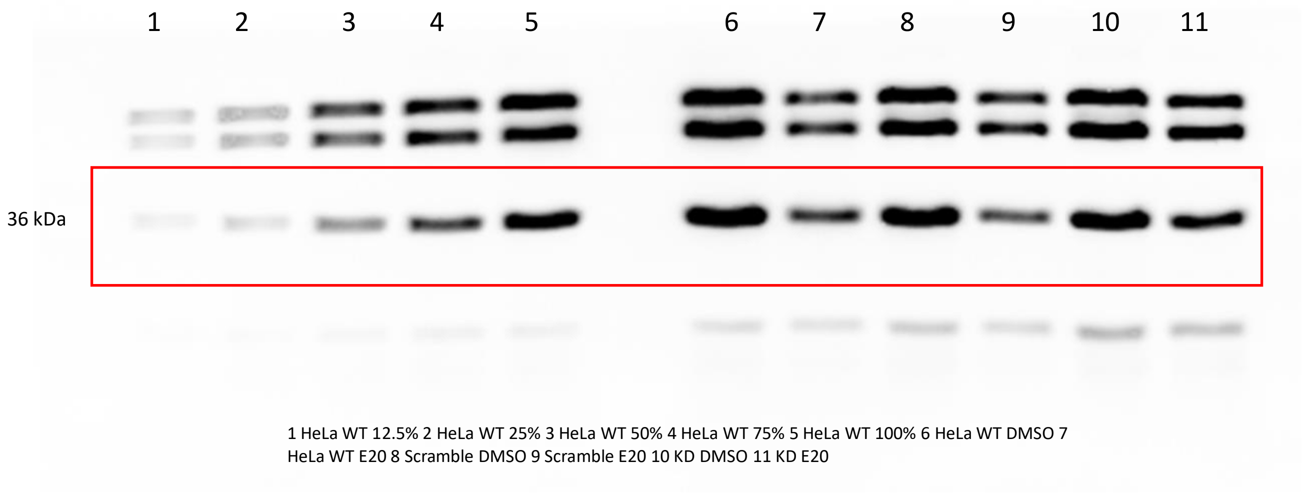

Tubulin

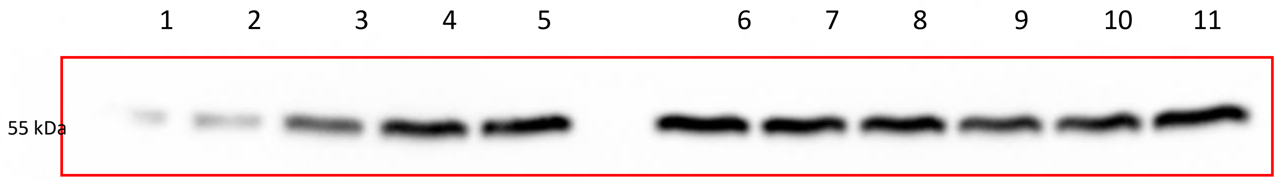

1 HeLa WT 12.5%

2 HeLa WT 25%

3 HeLa WT 50%

4 HeLa WT 75%

5 HeLa WT 100%

6 HeLa WT DMSO

7 HeLa WT E20

8 Scramble DMSO

9 Scramble E20

10 KD DMSO

11 KD E20

Additional file 1: Fig. 9A

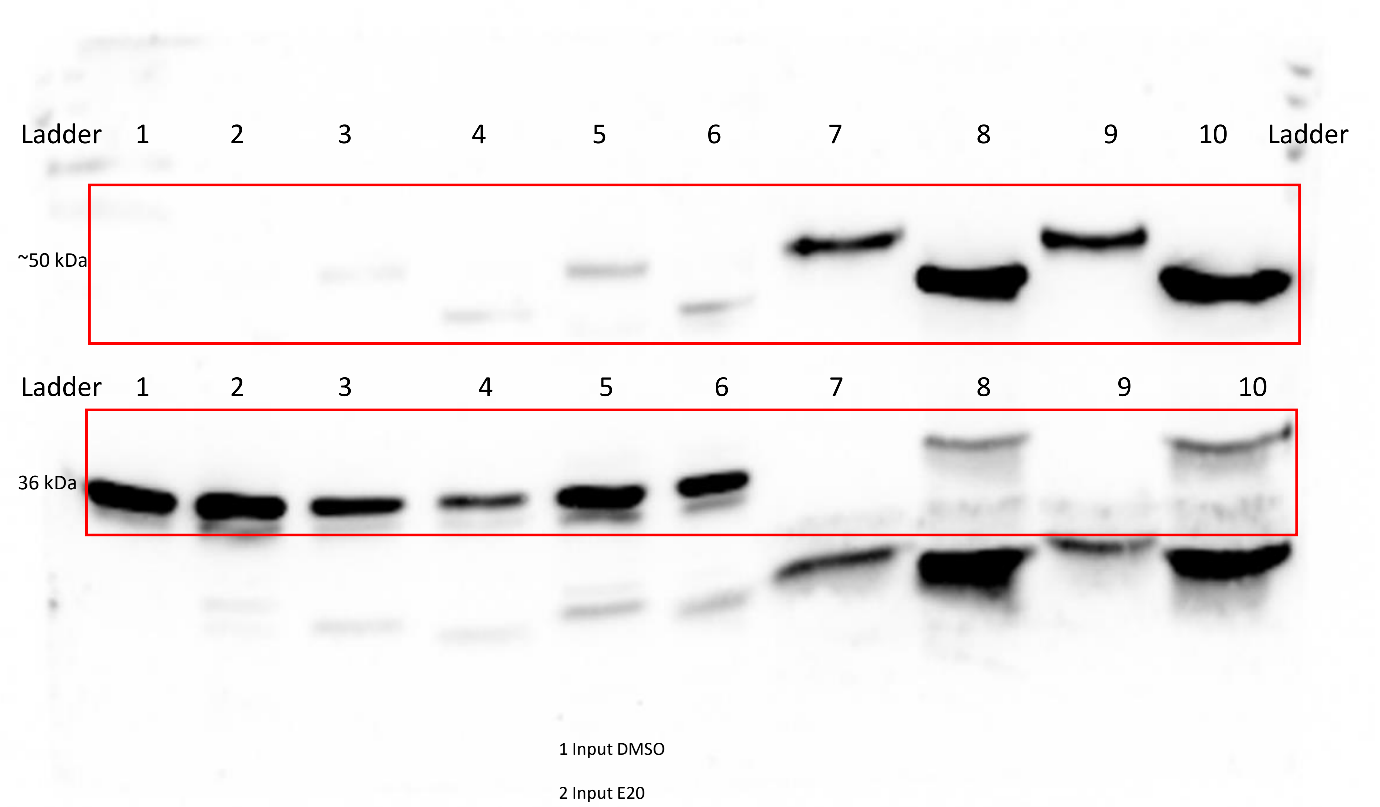

1 Input DMSO

2 Input E20

3 Flow-through DMSO IP IgG

4 Flow-through DMSO IP HuR

5 Flow-through E20 IP IgG

6 Flow-through E20 IP HuR

7 IP DMSO IP IgG

8 IP DMSO IP HuR

9 IP E20 IP IgG

10 IP E20 IP HuR

Additional file 1: Fig. 11A

IRP2

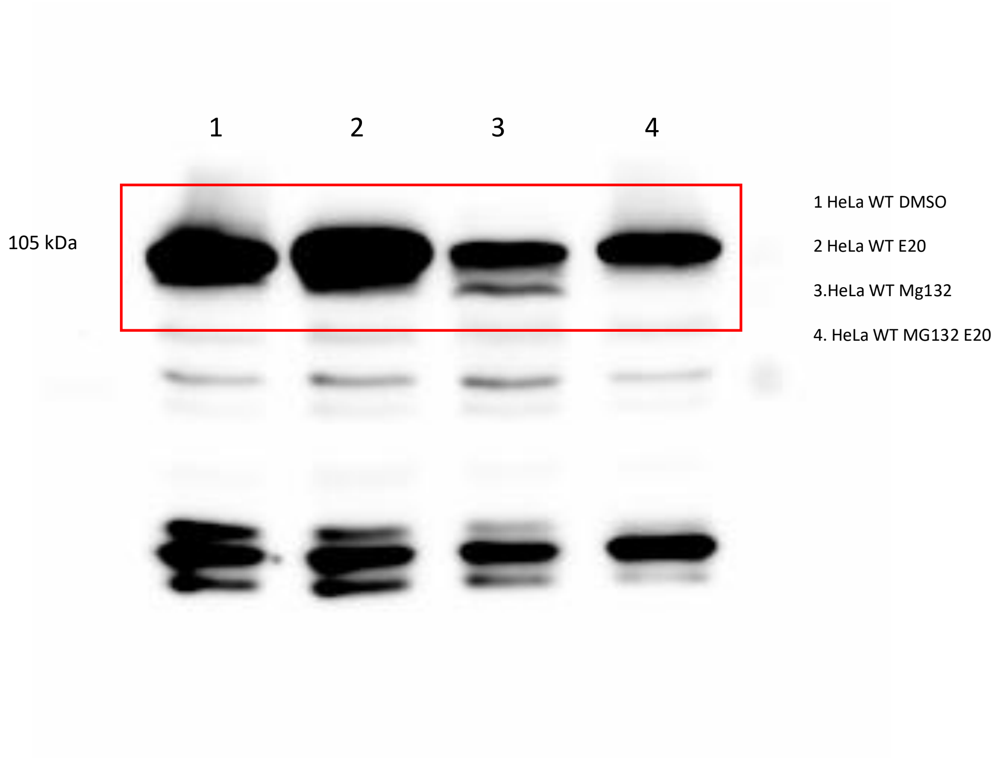

FTL

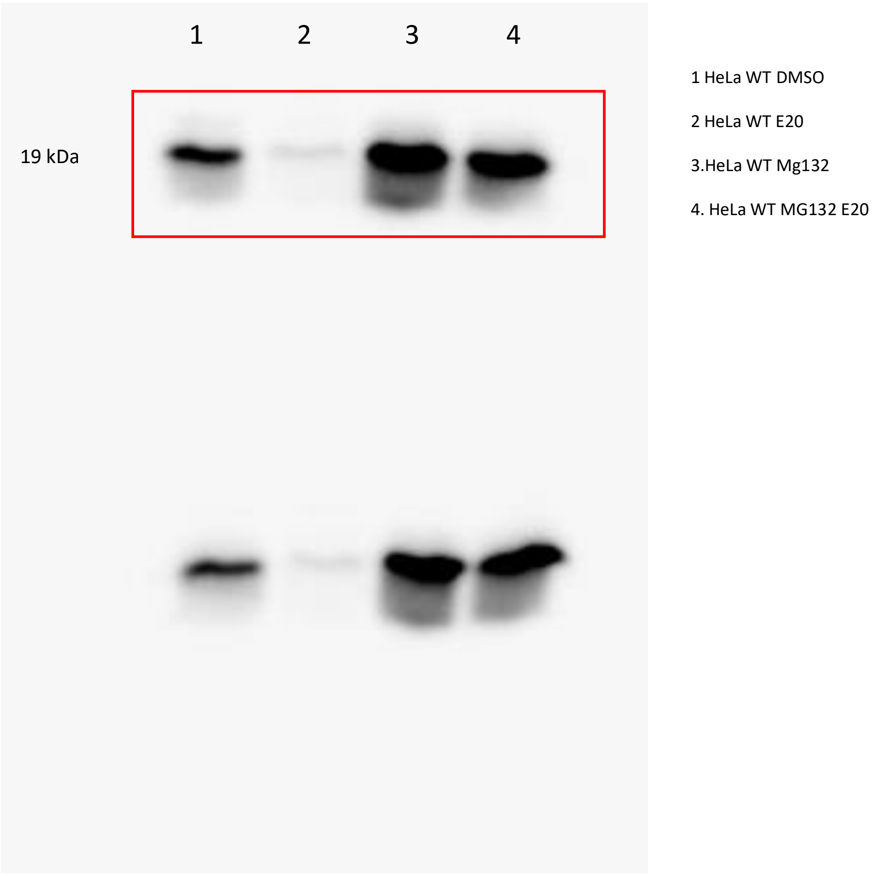

FTH

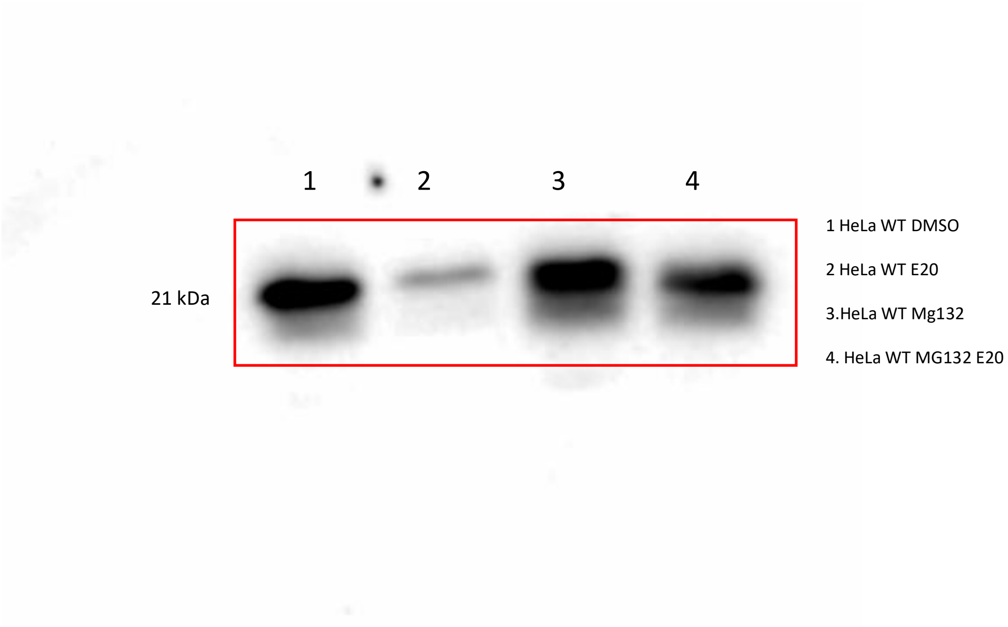
1
2
3
4
21 kDa
1 HeLa WT DMSO
2 HeLa WT E20
3.HeLa WT Mg132
4. HeLa WT MG132 E20

CGA

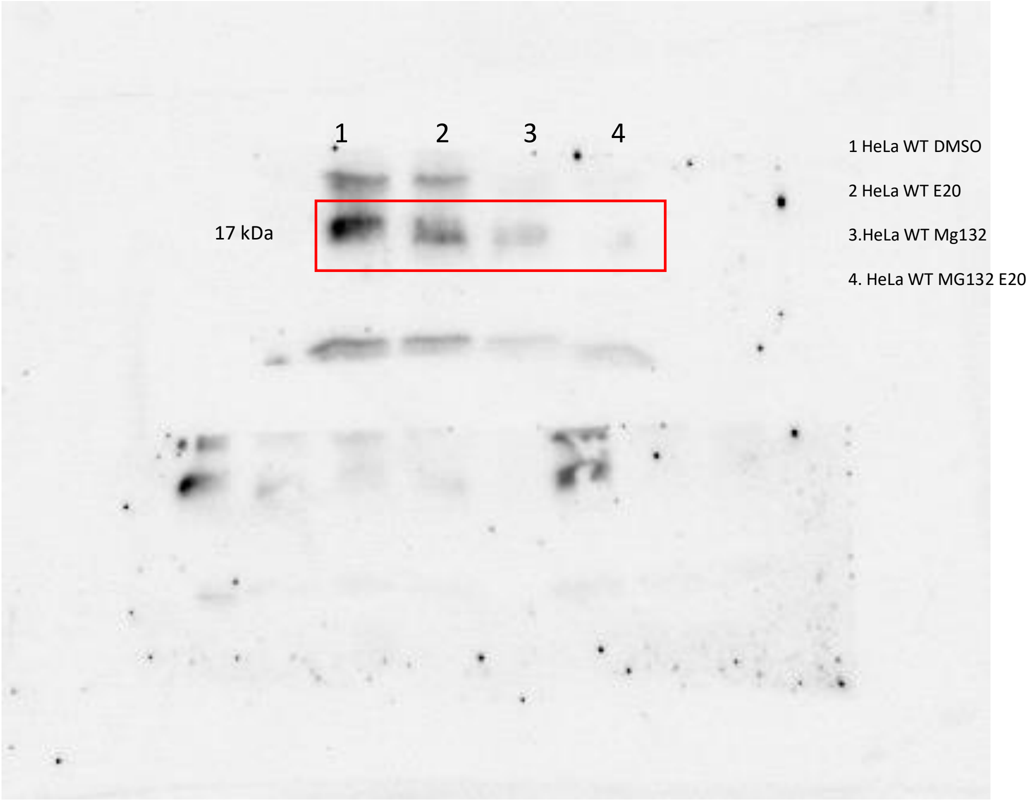
1
2
3
4
17 kDa
1 HeLa WT DMSO
2 HeLa WT E20
3.HeLa WT Mg132
4. HeLa WT MG132 E20

HuR

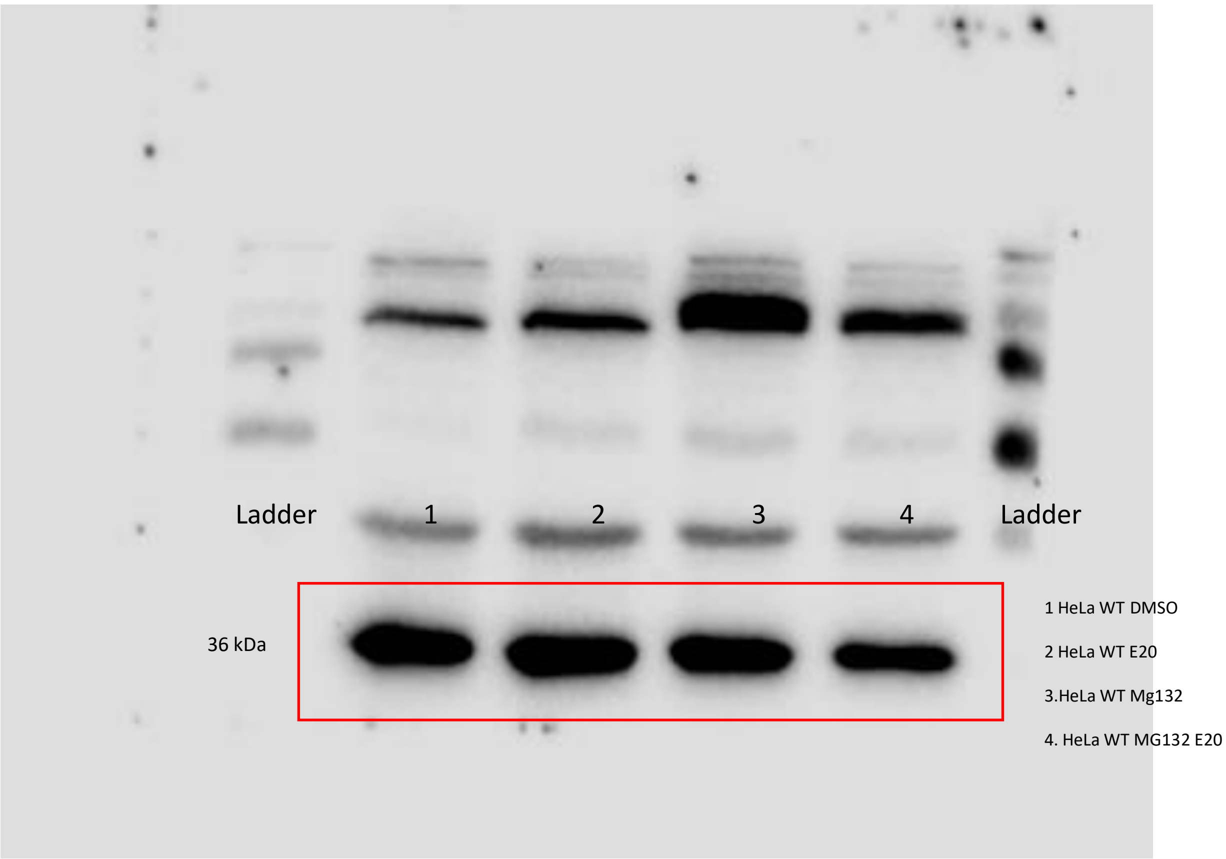
Ladder
1
2
3
4
Ladder
36 kDa
1 HeLa WT DMSO
2 HeLa WT E20
3.HeLa WT Mg132
4. HeLa WT MG132 E20

Tubulin

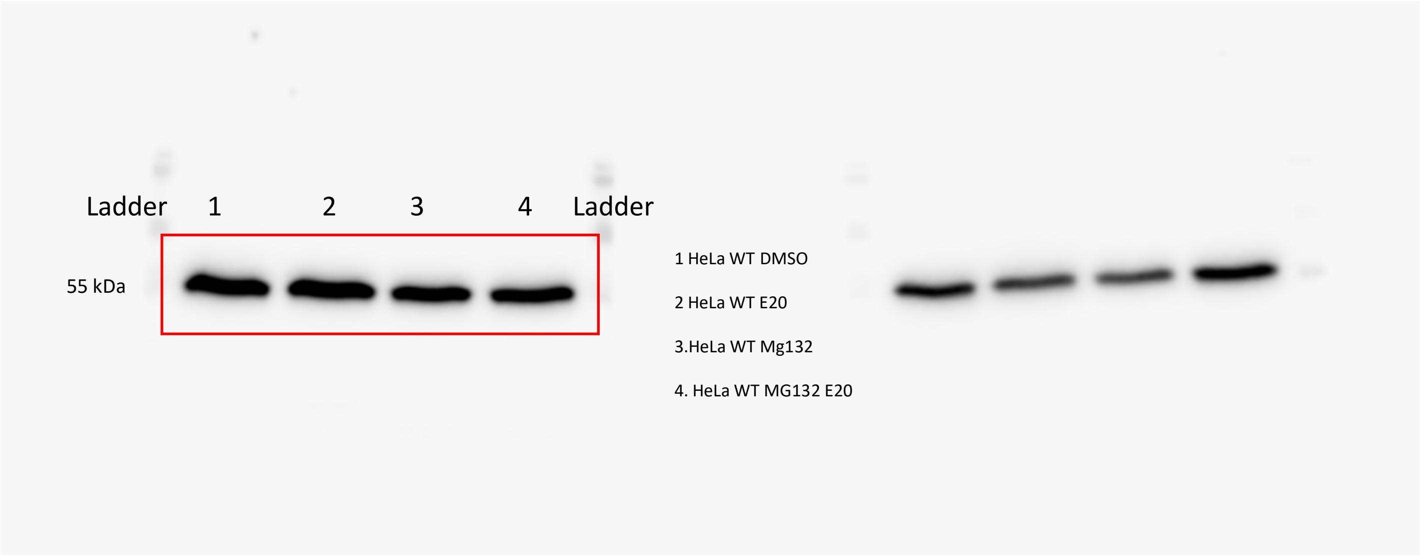
Ladder
1
2
3
4
Ladder
55 kDa
1 HeLa WT DMSO
2 HeLa WT E20
3.HeLa WT Mg132
4. HeLa WT MG132 E20

Ub

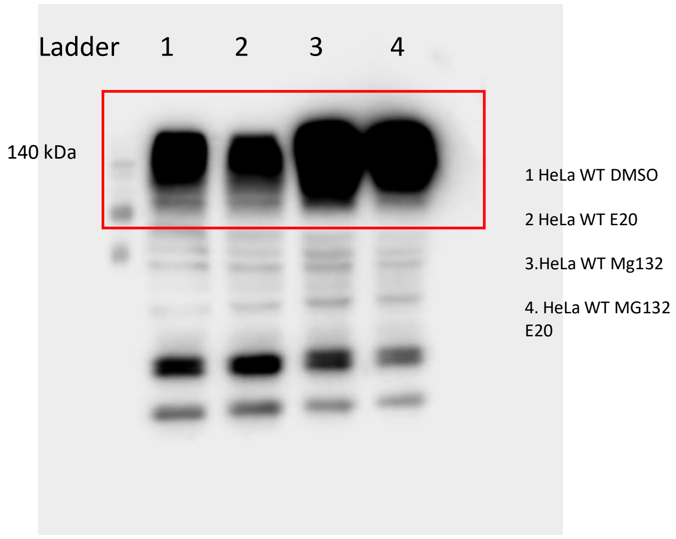
Ladder 1 2 3 4
140 kDa
1 HeLa WT DMSO
2 HeLa WT E20
3.HeLa WT Mg132
4. HeLa WT MG132 E20
